# Design, Synthesis, Biological Activity Evaluation, and Molecular Docking of 2-Aminopyrimidine-Based PKMYT1 Inhibitors

**DOI:** 10.3390/biomedicines14081876

**Published:** 2026-08-21

**Authors:** Shizhe Yuan, Chuanxu Su, Chenxi Zhang, Haoyu Zhang, Jinyu Yu, Nian Liu, Cunzheng Fan, Zixuan Gao, Zirui Luo, Yin Sun, Dongmei Zhao, Maosheng Cheng

**Affiliations:** Key Laboratory of Structure-Based Drug Design and Discovery, Ministry of Education, School of Pharmaceutical Engineering, Shenyang Pharmaceutical University, 103 Wenhua Road, Shenhe District, Shenyang 110016, China

**Keywords:** PKMYT1 inhibitors, *CCNE1*, CADD, cancer, kinase, selectivity

## Abstract

**Introduction:** PKMYT1 is a WEE-family G2/M cell cycle checkpoint kinase commonly overexpressed in a broad spectrum of human malignancies. WEE1 exclusively phosphorylates CDK1 at Tyr15, whereas PKMYT1 targets both Thr14 and Tyr15. Unlike WEE1 inhibition, PKMYT1 suppression triggers synthetic lethality with *CCNE1*. Nearly all disclosed PKMYT1 inhibitors so far fall into structural analogs originating from **RP-6306**, making the discovery of PKMYT1 inhibitors with chemotypes distinct from **RP-6306** crucial. **Methods:** The compounds were structurally optimized using CADD, synthesized, and characterized by ^1^H NMR, ^13^C NMR, HRMS, and HPLC. They were then assessed for kinase binding affinity via the LanthaScreen^TM^ Eu kinase binding assay, for cellular activity using the CCK-8 assay, and for cell-cycle distribution by flow cytometry, along with investigations into related mechanisms. **Results:** This study yielded 24 compounds of 2-aminopyrimidine through substituent derivatization of the pyrimidine scaffold. Among these derivatives, **MS13** exhibited potent kinase binding affinity against PKMYT1 (IC_50_ = 0.86 nM) and demonstrated strong anti-proliferative activity against *CCNE1* high-amplification OVCAR3 cells and HCC1569 cells (IC_50-OVCAR3_ = 1.52 μM, IC_50-HCC1569_ = 0.66 μM). Additionally, it showed some selectivity towards A549 and HEK293T cells, with SI values of 4.31 (A549/OVCAR3), 9.92 (A549/HCC1569), 2.04 (HEK293T/OVCAR3), and 4.70 (HEK293T/HCC1569). Compound **MS13** dose-dependently suppressed clonogenicity and triggered S-phase cell cycle blockade. Pharmacokinetic studies showed moderate hepatic microsomal stability (t_1/2_ = 32.2 min). Molecular dynamics simulations indicated a favorable binding mode between compound **MS13** and PKMYT1 (docking score: −9.322 kcal/mol). **Conclusions:** **MS13** is a promising highly potent tool compound that provides a clear direction for the future optimization of PKMYT1 inhibitors.

## 1. Introduction

Breast and ovarian cancers remain devastating malignancies in women, with profound impacts on patients’ well-being, reproductive capacity, and quality of life. Worldwide, breast cancer has the highest incidence among all cancers in women and is the second leading cause of cancer-related mortality [1]. Meanwhile, *CCNE1* amplification occurs in about 20% of ovarian tumors [2]. The occurrence of cancers, including breast and ovarian cancers, is closely related to the cell cycle, in which the G2/M checkpoint plays a crucial “gatekeeping” role in maintaining genomic stability [3,4]. Cells with DNA damage heavily rely on the time window provided by the G2/M phase for gene repair [5,6,7]. The G2/M checkpoint inhibits the activity of cyclin-dependent kinase 1 (CDK1), thereby preventing mitosis, a process primarily regulated by the WEE protein kinase family members WEE1 and PKMYT1 [8]. PKMYT1 is a membrane-associated tyrosine/threonine protein kinase, predominantly distributed in the endoplasmic reticulum and Golgi apparatus, capable of phosphorylating CDK1 at Thr14/Tyr15, thereby inhibiting the activity of the CDK1–cyclin B complex and preventing DNA-damaged cells from entering the M phase, thus completing the repair of DNA damage [9].

Research has shown a significant synthetic lethality effect between PKMYT1 and *CCNE1* amplification or *FBXW7* deletion [2,10]. Cells with high *CCNE1* amplification lead to increased DNA replication stress, resulting in the premature accumulation of the CDK1–cyclin B complex, which creates a high dependency on PKMYT1 to inhibit CDK1 [11,12,13]. This dependency creates a synthetic lethal vulnerability; when PKMYT1 is inhibited, CDK1 is prematurely activated, causing cells with DNA damage due to *CCNE1* amplification to directly enter mitosis, leading to chromosomal segregation abnormalities and catastrophic mitotic cell death [14,15,16]. Furthermore, PKMYT1 is non-essential for the survival of normal cells, and targeting PKMYT1 can significantly reduce off-target toxicity, providing greater selectivity for treatment [17,18].

Before 2021, PKMYT1 inhibitor research remained relatively limited. This scenario shifted with the debut of **RP-6306**, a first-in-class agent that entered Phase I studies in 2021 and ignited widespread interest in PKMYT1 as a therapeutic target [19]. The PKMYT1 inhibitor **RP-6306** (also known as **Lunresertib**) made its clinical debut in Phase I trials in April 2021 and progressed to Phase II by May 2023. Its primary indication is CDK4/6-resistant breast cancer. Notably, it was not evaluated as a monotherapy in Phase II but rather in combination with gemcitabine. **XL-495**, a selective PKMYT1 inhibitor structurally analogous to **RP-6306**, initiated Phase I trials in October 2024 for urological tumors, urothelial carcinoma, and advanced solid malignancies [20]. However, this clinical program was terminated in July 2025, as the early-phase human data failed to demonstrate a favorable risk–benefit profile, despite promising preclinical efficacy. **QLS-1209**, which differs from **RP-6306** by the incorporation of four additional fluorine atoms, entered Phase I trials in April 2025 for advanced solid tumors [21]. **ACR-2316** and **SGR-3515**, both dual inhibitors of PKMYT1 and WEE1, commenced Phase I studies in October 2024 and June 2024, respectively, with indications in advanced solid tumors [22,23]. Regrettably, the chemical structures of **QLS-1209**, **ACR-2316**, and **SGR-3515** remain undisclosed to date.

**Figure 1:**
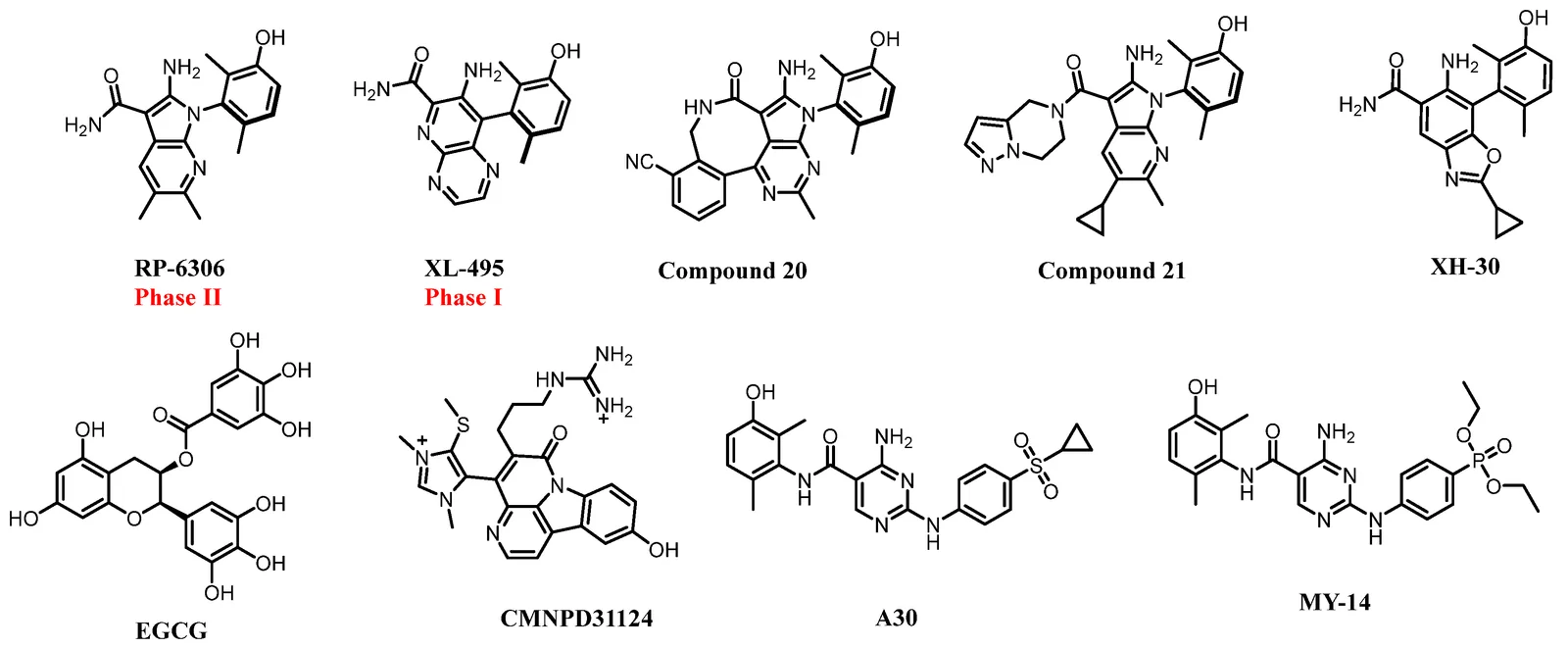
Representative PKMYT1 inhibitor chemical structures.

Moreover, most PKMYT1 inhibitors remain in preclinical research, with **RP-6306** derivatives representing the predominant class, such as Compound **20** developed by Simcere Pharmaceutical [24], Compound **21** developed by Insilico Medicine [25], and **XH-30** developed by China Pharmaceutical University [26]. In addition, certain natural products reported to exhibit PKMYT1 inhibitory activity are not amenable to synthetic modification, such as **EGCG** [27] and **CMNPD31124** [28] (as shown in Figure 1), further limiting the development of PKMYT1 inhibitors. Therefore, there is an urgent need to develop chemical scaffolds distinct from **RP-6306** with high selectivity to promote the widespread clinical application of this target. This study aims to rationally design and synthesize a series of 2-aminopyrimidine derivatives that differ from **RP-6306**. Through strategic modification of substituents and exploration of different binding modes, this study aims to provide a preliminary tool molecule that offers a basis for subsequent compound optimization.

## 2. Method

### 2.1. Materials and Instruments

All commercially available reagents and solvents were used as received without further purification. Reaction progress was monitored by thin-layer chromatography (TLC) on pre-coated silica gel GF254 plates, with visualization under UV light. ^1^H and ^13^C NMR spectral data were recorded in DMSO-*d_6_* on Bruker Avance spectrometers (Bruker BioSpin GmbH, Rheinstetten, Germany) at 400 or 600 MHz; tetramethylsilane was used as the internal standard. Low-resolution electrospray ionization mass spectra (ESI-MS) were acquired on an Agilent 1100 LC-MS system (Agilent Technologies, Inc., Santa Clara, CA, USA), whereas high-resolution mass spectra (HRMS) were obtained using an Agilent Q-TOF 6530 or 6550 instrument (Agilent Technologies, Inc., Santa Clara, CA, USA). Melting points were determined on BÜCHI B-540 melting point (BÜCHI Labortechnik AG, Flawil, Switzerland) apparatus using open capillary tubes. Compound purity (>95%, area %) was assessed by reversed-phase high-performance liquid chromatography (RP-HPLC) on a Hua Spectrum S6000PLUS UHPLC system (Acchrom‑Tech, Beijing, China) equipped with an Agilent ZORBAX Eclipse XDB-C18 column (4.6 × 150 mm, 5 μm), eluting with ACN/H_2_O (8:2, *v*/*v*) at a flow rate of 1.0 mL/min and a column temperature of 25 °C. The binding affinity for PKMYT1 was assessed using the LanthaScreen^TM^ (Thermo Fisher Scientific, Inc., Carlsbad, CA, USA) Eu kinase binding assay platform, comprising PKMYT1 kinase, Eu-labeled anti-GST antibody, and Tracer178, with fluorescence signals detected at 615 nm and 665 nm via a TR-FRET plate reader (Thermo Fisher Scientific). The cell lines employed included OVCAR3 (Procell, CL-0178), HCC1569 (BNCC, BNCC341698), A549 (Procell, CL-0016), and HEK293T (Procell, CL-0005). Cell proliferation was evaluated using the CCK-8 reagent in 96-well and 6-well plates, while colony formation was assessed using PBS, 4% paraformaldehyde, and crystal violet. For cell cycle analysis, cells were fixed and stained with trypsin (without EDTA), 70% ethanol, propidium iodide (PI), and RNase A (Beyotime kit), and then analyzed on a BD Accuri^TM^ C6 Plus flow cytometer (BD Biosciences, San Jose, CA, USA). The software used included GraphPad Prism 8 software, ImageJ 1.54 software, FlowJo v10 software, Schrödinger 2021-2 software, and PyMOL 3.0.

### 2.2. Synthesis and Analytical Characterization

Full spectroscopic and analytical data are provided in the Supplementary Materials.

2-chloro-4-(phenylamino)-*N*-(3-((tert-butyldimethylsilyl)oxy)-2,6-dimethylphenyl)pyrimidine-5-carboxamide (**MS1a**):

A solution of **11b** (120 mg, 0.28 mmol), aniline (27.9 mg, 0.30 mmol), and DIPEA (73.0 mg, 0.56 mmol) in *n*-BuOH (5 mL) was stirred at 100 °C for 2 h. After completion, the reaction mixture was concentrated under reduced pressure and purified by preparative TLC (large plate) to afford the product as a white solid (91.5 mg, 67.8% yield). ^1^H NMR (600 MHz, DMSO-*d*_6_) δ 10.94 (s, 1H), 10.03 (s, 1H), 8.80 (s, 1H), 7.44 − 7.40 (m, 2H), 7.19 (dd, *J* = 8.6, 7.4 Hz, 2H), 6.97 − 6.92 (m, 1H), 6.81 (d, *J* = 8.3 Hz, 1H), 6.55 (d, *J* = 8.2 Hz, 1H), 1.92 (s, 3H), 1.85 (s, 3H), 0.78 (s, 9H), −0.00 (s, 6H). HRMS (ESI) for C_25_H_31_ClN_4_O_2_Si [M-H]^−^: calcd, 481.1827, found, 481.1842.

2-amino-4-(phenylamino)-*N*-(3-hydroxy-2,6-dimethylphenyl)pyrimidine-5-carboxamide (**MS1**):

White solid; yield: 76.9%; mp: 133.8- 134.5 °C; ^1^H NMR (600 MHz, DMSO-*d*_6_) δ 11.29 (s, 1H), 9.62 (s, 1H), 9.23 (s, 1H), 8.83 (s, 1H), 7.76 − 7.74 (m, 1H), 7.30 (dd, *J* = 8.6, 7.3 Hz, 2H), 7.04 − 6.99 (m, 3H), 6.89 (d, *J* = 8.2 Hz, 1H), 6.72 (d, *J* = 8.2 Hz, 1H), 2.07 (s, 3H), 1.99 (s, 3H). ^13^C NMR (151 MHz, DMSO-*d*_6_) δ 166.51, 163.83, 160.30, 158.71, 154.19, 139.77, 136.00, 129.23, 127.32, 126.09, 122.92, 122.88, 120.61, 113.67, 99.06, 18.03, 11.62. HRMS (ESI) for C_19_H_19_N_5_O_2_ [M+Na]^+^: calcd, 372.1436, found, 372.1436. HPLC: 96.163%.

2-amino-4-((4-fluorophenyl)amino)-*N*-(3-hydroxy-2,6-dimethylphenyl)pyrimidine-5-carboxamide (**MS2**):

White solid; yield: 60.8%; mp: 263.2–264.0 °C; ^1^H NMR (600 MHz, DMSO-*d*_6_) δ 11.26 (s, 1H), 9.59 (s, 1H), 9.17 (s, 1H), 8.81 (s, 1H), 7.77 (dd, *J* = 9.1, 5.0 Hz, 2H), 7.13 (t, *J* = 8.9 Hz, 2H), 7.02 (s, 2H), 6.89 (d, *J* = 8.2 Hz, 1H), 6.70 (d, *J* = 8.2 Hz, 1H), 2.07 (s, 3H), 2.00 (s, 3H). ^13^C NMR (151 MHz, DMSO-*d*_6_) δ 166.47, 163.86, 160.21, 158.71, 158.13 (d, *J* = 239.4 Hz), 154.15, 136.12 (d, *J* = 2.5 Hz), 135.99, 127.35, 126.14, 122.90, 122.46 (d, *J* = 7.6 Hz), 115.70 (d, *J* = 22.1 Hz), 113.66, 98.91, 18.02, 11.62. HRMS (ESI) for C_19_H_18_FN_5_O_2_ [M-H]^−^: calcd, 366.1367, found, 366.1363. HPLC: 98.284%.

2-amino-4-((4-chlorophenyl)amino)-*N*-(3-hydroxy-2,6-dimethylphenyl)pyrimidine-5-carboxamide (**MS3**):

White solid; yield: 62.1%; mp: 301.5–302.3 °C; ^1^H NMR (600 MHz, DMSO-*d*_6_) δ 11.37 (d, *J* = 2.6 Hz, 1H), 9.62 (s, 1H), 9.18 (s, 1H), 8.83 (s, 1H), 7.80 (d, *J* = 8.9 Hz, 2H), 7.32 (d, *J* = 8.9 Hz, 2H), 7.09 (s, 2H), 6.89 (d, *J* = 8.1 Hz, 1H), 6.70 (d, *J* = 8.2 Hz, 1H), 2.07 (d, *J* = 2.0 Hz, 3H), 2.00 (d, *J* = 3.4 Hz, 3H). ^13^C NMR (151 MHz, DMSO) δ 166.40, 163.77, 160.18, 158.76, 154.16, 138.71, 135.93, 128.99, 127.36, 126.43, 126.12, 122.88, 122.20, 113.69, 99.11, 18.02, 11.62. HRMS (ESI) for C_19_H_18_ClN_5_O_2_ [M-H]^−^: calcd, 382.1071, found, 382.1070. HPLC: 95.082%.

2-amino-4-((4-(dimethylphosphoryl)phenyl)amino)-*N*-(3-hydroxy-2,6-dimethylphenyl)pyrimidine-5-carboxamide (**MS4**):

White solid; yield: 60.0%; mp: 297.3–298.0 °C; ^1^H NMR (600 MHz, DMSO-*d*_6_) δ 11.51 (s, 1H), 9.65 (s, 1H), 9.20 (s, 1H), 8.85 (s, 1H), 7.90 (dd, *J* = 8.7, 2.1 Hz, 2H), 7.67 (dd, *J* = 11.1, 8.5 Hz, 2H), 7.14 (s, 2H), 6.90 (d, *J* = 8.2 Hz, 1H), 6.71 (d, *J* = 8.2 Hz, 1H), 2.07 (s, 3H), 2.00 (s, 3H), 1.63 (d, *J* = 13.2 Hz, 6H). ^13^C NMR (151 MHz, DMSO-*d*_6_) δ 165.32, 162.75, 159.21, 157.84, 153.10, 141.30 (d, *J* = 2.8 Hz), 134.84, 129.92 (d, *J* = 10.4 Hz), 128.23 (d, *J* = 100.4 Hz), 126.31, 125.03, 121.80, 119.04 (d, *J* = 11.6 Hz), 112.63, 98.25, 17.29 (d, *J* = 70.8 Hz), 16.95, 10.55. HRMS (ESI) for C_21_H_24_N_5_O_3_P [M-H]^−^: calcd, 424.1539, found, 424.1540. HPLC: 98.402%.

2-amino-4-((4-(1,1-dioxidothiomorpholino)phenyl)amino)-*N*-(3-hydroxy-2,6-dimethylphenyl)pyrimidine-5-carboxamide (**MS5**):

Brown solid; yield: 35.9%; mp: 274.3–275.3 °C; ^1^H NMR (600 MHz, DMSO-*d*_6_) δ 11.07 (s, 1H), 9.55 (s, 1H), 9.16 (s, 1H), 8.76 (s, 1H), 7.59 (d, *J* = 8.4 Hz, 2H), 6.98 (d, *J* = 8.6 Hz, 2H), 6.94 (s, 2H), 6.89 (d, *J* = 8.2 Hz, 1H), 6.69 (d, *J* = 8.2 Hz, 1H), 3.77 − 3.60 (m, 4H), 3.21 − 3.07 (m, 4H), 2.07 (s, 3H), 1.99 (s, 3H). ^13^C NMR (151 MHz, DMSO) δ 166.49, 163.67, 160.11, 154.14, 144.01, 136.03, 132.17, 130.12, 127.34, 126.13, 122.89, 122.28, 117.04, 113.62, 98.89, 50.35, 47.91, 18.02, 11.62. HRMS (ESI) for C_23_H_26_N_6_O_4_S [M-H]^−^: calcd, 481.1658, found, 481.1658. HPLC: 97.542%.

2-amino-4-((4-(trifluoromethyl)phenyl)amino)-*N*-(3-hydroxy-2,6-dimethylphenyl)pyrimidine-5-carboxamide (**MS6**):

White solid; yield: 55.6%; mp: 287.9–288.9 °C; ^1^H NMR (600 MHz, DMSO-*d*_6_) δ 11.62 (s, 1H), 9.67 (s, 1H), 9.19 (s, 1H), 8.87 (s, 1H), 7.99 (d, *J* = 8.6 Hz, 2H), 7.62 (d, *J* = 8.6 Hz, 2H), 7.17 (s, 2H), 6.90 (d, *J* = 8.2 Hz, 1H), 6.71 (d, *J* = 8.2 Hz, 1H), 2.08 (s, 3H), 2.00 (s, 3H). ^13^C NMR (151 MHz, DMSO-*d*_6_) δ 165.26, 162.72, 159.21, 157.95, 153.10, 142.29, 134.79, 126.32, 125.26 (q, *J* = 3.8 Hz), 125.04, 123.90 (q, *J* = 271.0 Hz), 121.81, 121.59 (q, *J* = 32.0 Hz), 119.38, 112.66, 98.31, 16.95, 10.55. HRMS (ESI) for C_20_H_18_F_3_N_5_O_2_ [M-H]^−^: calcd, 416.1335, found, 416.1331. HPLC: 97.777%.

2-amino-4-((4-methoxyphenyl)amino)-*N*-(3-hydroxy-2,6-dimethylphenyl)pyrimidine-5-carboxamide (**MS7**):

White solid; yield: 56.7%; mp: 291.2–291.9 °C; ^1^H NMR (600 MHz, DMSO-*d*_6_) δ 11.07 (s, 1H), 9.54 (s, 1H), 9.16 (s, 1H), 8.77 (s, 1H), 7.63 (d, *J* = 9.0 Hz, 2H), 6.92 (s, 2H), 6.88 (t, *J* = 8.5 Hz, 3H), 6.69 (d, *J* = 8.2 Hz, 1H), 3.73 (s, 3H), 2.07 (s, 3H), 1.99 (s, 3H). ^13^C NMR (151 MHz, DMSO) δ 165.48, 162.81, 159.12, 157.43, 154.29, 153.06, 134.99, 131.73, 126.26, 125.07, 121.83, 121.34, 113.30, 112.54, 97.71, 54.56, 16.96, 10.55. HRMS (ESI) for C_20_H_21_N_5_O_3_ [M+Na]^+^: calcd, 402.1542, found, 402.1545. HPLC: 99.355%.

2-amino-4-((4-(methylsulfonyl)phenyl)amino)-*N*-(3-hydroxy-2,6-dimethylphenyl)pyrimidine-5-carboxamide (**MS8**):

White solid; yield: 63.9%; mp: 308.4–309.3 °C; ^1^H NMR (600 MHz, DMSO-*d*_6_) δ 11.71 (s, 1H), 9.69 (s, 1H), 9.19 (s, 1H), 8.88 (s, 1H), 8.04 (d, *J* = 8.9 Hz, 2H), 7.80 (d, *J* = 8.8 Hz, 2H), 7.22 (s, 2H), 6.90 (d, *J* = 8.2 Hz, 1H), 6.71 (d, *J* = 8.2 Hz, 1H), 3.18 (s, 3H), 2.07 (s, 3H), 1.99 (s, 3H). ^13^C NMR (151 MHz, DMSO) δ 165.19, 162.68, 159.11, 158.02, 153.09, 143.24, 134.72, 132.83, 127.42, 126.29, 124.98, 121.76, 119.13, 112.66, 98.40, 43.26, 16.92, 10.53. HRMS (ESI) for C_20_H_21_N_5_O_4_S [M-H]^−^: calcd, 426.1236, found, 426.1248. HPLC: 96.983%.

2-amino-4-((4-(ethylsulfonyl)phenyl)amino)-*N*-(3-hydroxy-2,6-dimethylphenyl)pyrimidine-5-carboxamide (**MS9**):

White solid; yield: 58.3%; mp: 287.5–288.1 °C; ^1^H NMR (600 MHz, DMSO-*d*_6_) δ 11.74 (s, 1H), 9.69 (s, 1H), 9.19 (s, 1H), 8.89 (s, 1H), 8.06 (d, *J* = 8.8 Hz, 2H), 7.75 (d, *J* = 8.8 Hz, 2H), 7.23 (s, 2H), 6.90 (d, *J* = 8.2 Hz, 1H), 6.71 (d, *J* = 8.2 Hz, 1H), 3.25 (q, *J* = 7.3 Hz, 2H), 2.08 (s, 3H), 2.00 (s, 3H), 1.10 (t, *J* = 7.3 Hz, 3H). ^13^C NMR (151 MHz, DMSO) δ 166.27, 163.77, 160.20, 159.11, 154.17, 144.49, 135.81, 131.33, 129.37, 127.40, 126.09, 122.86, 120.20, 113.75, 99.53, 49.92, 18.01, 11.62, 7.77. HRMS (ESI) for C_21_H_23_N_5_O_4_S [M-H]^−^: calcd, 440.1393, found, 440.1393. HPLC: 97.362%.

2-amino-4-((4-(isopropylsulfonyl)phenyl)amino)-*N*-(3-hydroxy-2,6-dimethylphenyl)pyrimidine-5-carboxamide (**MS10**):

White solid; yield: 55.8%; mp: 255.6–256.5 °C; ^1^H NMR (600 MHz, DMSO-*d*_6_) δ 11.75 (s, 1H), 9.69 (s, 1H), 9.19 (s, 1H), 8.89 (s, 1H), 8.07 (d, *J* = 8.9 Hz, 2H), 7.71 (d, *J* = 8.9 Hz, 2H), 7.23 (s, 2H), 6.90 (d, *J* = 8.2 Hz, 1H), 6.71 (d, *J* = 8.1 Hz, 1H), 3.38 − 3.32 (m, 1H), 2.08 (s, 3H), 2.00 (s, 3H), 1.15 (d, *J* = 6.8 Hz, 6H). ^13^C NMR (151 MHz, DMSO-*d*_6_) δ 166.27, 163.76, 160.20, 159.11, 154.17, 144.59, 135.80, 130.17, 129.46, 127.40, 126.09, 122.86, 120.08, 113.75, 99.54, 54.80, 18.01, 15.81, 11.63. HRMS (ESI) for C_22_H_25_N_5_O_4_S [M-H]^−^: calcd, 454.1549, found, 454.1547. HPLC: 95.089%.

2-amino-4-((4-(cyclopropylsulfonyl)phenyl)amino)-*N*-(3-hydroxy-2,6-dimethylphenyl)pyrimidine-5-carboxamide (**MS11**):

White solid; yield: 57.2%; mp: 260.3–261.3 °C; ^1^H NMR (600 MHz, DMSO-*d*_6_) δ 11.71 (s, 1H), 9.69 (s, 1H), 9.20 (s, 1H), 8.88 (s, 1H), 8.04 (d, *J* = 8.9 Hz, 2H), 7.76 (d, *J* = 8.8 Hz, 2H), 7.21 (s, 2H), 6.90 (d, *J* = 8.2 Hz, 1H), 6.71 (d, *J* = 8.1 Hz, 1H), 2.82 (tt, *J* = 7.9, 4.8 Hz, 1H), 2.07 (s, 3H), 1.99 (s, 3H), 1.11 − 0.90 (m, 4H). ^13^C NMR (151 MHz, DMSO) δ 165.20, 162.69, 159.12, 158.03, 153.10, 143.24, 134.73, 132.52, 127.70, 126.31, 125.00, 121.77, 119.25, 112.67, 98.43, 31.73, 16.93, 10.54, 4.74. HRMS (ESI) for C_22_H_23_N_5_O_4_S [M-H]^−^: calcd, 452.1393, found, 452.1395. HPLC: 96.615%.

2-amino-4-((4-(*N*,*N*-dimethylsulfamoyl)phenyl)amino)-*N*-(3-hydroxy-2,6-dimethylphenyl)pyrimidine-5-carboxamide (**MS12**):

White solid; yield: 59.6%; mp: 277.7–278.5 °C; ^1^H NMR (600 MHz, DMSO-*d*_6_) δ 11.72 (s, 1H), 9.69 (s, 1H), 9.20 (s, 1H), 8.88 (s, 1H), 8.06 (d, *J* = 8.9 Hz, 2H), 7.63 (d, *J* = 8.9 Hz, 2H), 7.21 (s, 2H), 6.90 (d, *J* = 8.2 Hz, 1H), 6.71 (d, *J* = 8.2 Hz, 1H), 2.59 (s, 6H), 2.08 (s, 3H), 2.00 (s, 3H). ^13^C NMR (151 MHz, DMSO) δ 166.28, 163.76, 160.18, 159.09, 154.17, 143.95, 135.81, 129.10, 127.51, 127.39, 126.09, 122.86, 120.11, 113.75, 99.49, 38.10, 18.00, 11.61. HRMS (ESI) for C_21_H_24_N_6_O_4_S [M+Na]^+^: calcd, 479.1477, found, 479.1503. HPLC: 98.265%.

2-amino-4-((4-nitrophenyl)amino)-*N*-(3-hydroxy-2,6-dimethylphenyl)pyrimidine-5-carboxamide (**MS13**):

Yellow solid; yield: 54.1%; mp: 292.2–293.3 °C; ^1^H NMR (600 MHz, DMSO-*d*_6_) δ 11.93 (s, 1H), 9.73 (s, 1H), 9.21 (s, 1H), 8.92 (s, 1H), 8.15 (d, *J* = 9.2 Hz, 2H), 8.07 (d, *J* = 9.3 Hz, 2H), 7.30 (s, 2H), 6.91 (d, *J* = 8.2 Hz, 1H), 6.72 (d, *J* = 8.2 Hz, 1H), 2.08 (s, 3H), 2.00 (s, 3H). ^13^C NMR (151 MHz, DMSO) δ 166.20, 163.74, 160.06, 159.26, 154.18, 146.14, 141.62, 135.74, 127.41, 126.07, 125.29, 122.85, 119.98, 113.78, 99.69, 18.00, 11.61. HRMS (ESI) for C_19_H_18_N_6_O_4_ [M-H]^−^: calcd, 393.1312, found, 393.1311. HPLC: 96.510%.

2-amino-4-((4-carbamoylphenyl)amino)-*N*-(3-hydroxy-2,6-dimethylphenyl)pyrimidine-5-carboxamide (**MS14**):

White solid; yield: 62.8%; mp: 267.9–268.8 °C; ^1^H NMR (600 MHz, DMSO-*d*_6_) δ 11.53 (s, 1H), 9.63 (s, 1H), 9.17 (s, 1H), 8.84 (s, 1H), 7.94 − 7.74 (m, 5H), 7.26 − 7.09 (m, 3H), 6.90 (d, *J* = 8.2 Hz, 1H), 6.70 (d, *J* = 8.2 Hz, 1H), 2.07 (s, 3H), 1.99 (s, 3H). ^13^C NMR (151 MHz, DMSO-*d*_6_) δ 167.85, 166.41, 163.83, 160.20, 158.89, 154.15, 142.46, 135.91, 128.90, 128.19, 127.38, 126.12, 122.88, 119.52, 113.70, 99.27, 18.01, 11.62. HRMS (ESI) for C_20_H_20_N_6_O_3_ [M-H]^−^: calcd, 391.1519, found, 391.1516. HPLC: 96.011%.

2-amino-4-((1-methyl-1*H*-pyrazol-4-yl)amino)-*N*-(3-hydroxy-2,6-dimethylphenyl)pyrimidine-5-carboxamide (**MS15**):

White solid; yield: 65.7%; mp: 323.8–324.8 °C; ^1^H NMR (600 MHz, DMSO-*d*_6_) δ 10.78 (s, 1H), 9.49 (s, 1H), 9.14 (s, 1H), 8.74 (s, 1H), 8.21 (s, 1H), 7.61 (s, 1H), 6.97 (s, 2H), 6.88 (d, *J* = 8.2 Hz, 1H), 6.68 (d, *J* = 8.2 Hz, 1H), 3.80 (s, 3H), 2.06 (s, 3H), 1.98 (s, 3H). ^13^C NMR (151 MHz, DMSO) δ 165.27, 163.00, 157.91, 156.99, 153.03, 134.99, 129.82, 126.21, 125.06, 121.80, 121.26, 121.17, 112.50, 97.69, 47.99, 16.95, 10.54. HRMS (ESI) for C_17_H_19_N_7_O_2_ [M-H]^−^: calcd, 352.1522, found, 352.1525. HPLC: 98.390%.

2-amino-4-((1-methyl-1*H*-pyrazol-5-yl)amino)-*N*-(3-hydroxy-2,6-dimethylphenyl)pyrimidine-5-carboxamide (**MS16**):

White solid; yield: 60.9%; mp: 282.9–283.8 °C; ^1^H NMR (600 MHz, DMSO-*d*_6_) δ 11.48 (d, *J* = 3.1 Hz, 1H), 9.66 (d, *J* = 4.1 Hz, 1H), 9.20 (d, *J* = 4.8 Hz, 1H), 8.86 (d, *J* = 5.2 Hz, 1H), 7.31 (t, *J* = 2.4 Hz, 1H), 7.12 (s, 2H), 6.89 (d, *J* = 8.1 Hz, 1H), 6.71 (dd, *J* = 8.2, 4.2 Hz, 1H), 6.62 (t, *J* = 2.4 Hz, 1H), 3.65 (d, *J* = 3.3 Hz, 3H), 2.08 (d, *J* = 3.1 Hz, 3H), 2.00 (d, *J* = 4.2 Hz, 3H). ^13^C NMR (151 MHz, DMSO) δ 165.34, 163.05, 158.26, 157.59, 153.10, 136.76, 136.43, 134.77, 126.34, 125.07, 121.85, 112.68, 97.90, 96.61, 34.49, 16.95, 10.55. HRMS (ESI) for C_17_H_19_N_7_O_2_ [M-H]^−^: calcd, 352.1522, found, 352.1523. HPLC: 99.329%.

2-amino-4-((4-nitrophenyl)amino)-*N*-(4-fluoro-3-hydroxy-2,6-dimethylphenyl)pyrimidine-5-carboxamide (**MS17**):

Yellow solid; yield: 58.0%; mp: 279.6–280.5 °C; ^1^H NMR (600 MHz, DMSO-*d*_6_) δ 11.86 (s, 1H), 9.72 (s, 1H), 9.27 (s, 1H), 8.90 (s, 1H), 8.15 (d, *J* = 9.3 Hz, 2H), 8.07 (d, *J* = 9.3 Hz, 2H), 7.31 (s, 2H), 6.95 (d, *J* = 11.4 Hz, 1H), 2.09 (s, 3H), 2.05 (s, 3H). ^13^C NMR (151 MHz, DMSO-*d*_6_) δ 165.31, 162.70, 158.97, 158.23, 149.42 (d, *J* = 237.3 Hz), 145.03, 140.59, 139.72 (d, *J* = 14.3 Hz), 130.11 (d, *J* = 2.6 Hz), 126.17 (d, *J* = 7.1 Hz), 125.59 (d, *J* = 3.5 Hz), 124.21, 118.96, 112.95 (d, *J* = 19.1 Hz), 98.49, 16.77, 10.97 (d, *J* = 2.8 Hz). HRMS (ESI) for C_19_H_17_FN_6_O_4_ [M-H]^−^: calcd, 411.1217, found, 411.1215. HPLC: 95.239%.

2-amino-4-((4-nitrophenyl)amino)-*N*-(3-fluoro-5-hydroxy-2,6-dimethylphenyl)pyrimidine-5-carboxamide (**MS18**):

Yellow solid; yield: 49.4%; mp: 301.1–302.0 °C; ^1^H NMR (600 MHz, DMSO-*d*_6_) δ 11.81 (s, 1H), 9.82 (s, 1H), 9.70 (s, 1H), 8.91 (s, 1H), 8.15 (d, *J* = 9.3 Hz, 2H), 8.07 (d, *J* = 9.3 Hz, 2H), 7.33 (s, 2H), 6.61 (d, *J* = 11.1 Hz, 1H), 1.98 (d, *J* = 1.8 Hz, 3H), 1.97 (s, 3H). ^13^C NMR (151 MHz, DMSO-*d*_6_) δ 165.27, 162.69, 158.98, 158.31, 157.86 (d, *J* = 237.8 Hz), 153.27 (d, *J* = 13.0 Hz), 145.00, 140.62, 135.81 (d, *J* = 9.0 Hz), 124.21, 119.00, 118.06 (d, *J* = 3.3 Hz), 111.91 (d, *J* = 17.2 Hz), 100.08 (d, *J* = 25.2 Hz), 98.41, 10.28, 8.98 (d, *J* = 3.6 Hz). HRMS (ESI) for C_19_H_17_FN_6_O_4_ [M-H]^−^: calcd, 411.1217, found, 411.1210. HPLC: 97.171%.

2-(methylamino)-4-((4-fluorophenyl)amino)-*N*-(3-hydroxy-2,6-dimethylphenyl)pyrimidine-5-carboxamide (**MS19**):

White solid; yield: 74.8%; mp: 271.1–271.9 °C; ^1^H NMR (600 MHz, DMSO-*d*_6_) δ 11.39 (s, 0.6H), 11.20 (s, 0.4H), 9.60 (s, 0.4H), 9.56 (s, 0.6H), 9.17 (s, 1H), 8.87 (s, 0.4H), 8.81 (s, 0.6H), 7.77 (dd, *J* = 8.9, 4.9 Hz, 2H), 7.55 − 7.52 (m, 0.6H), 7.45 − 7.40 (m, 0.4H), 7.21 − 7.07 (m, 2H), 6.89 (d, *J* = 8.2 Hz, 1H), 6.70 (d, *J* = 8.2 Hz, 1H), 2.86 (d, *J* = 4.7 Hz, 3H), 2.07 (s, 3H), 1.99 (s, 3H). ^13^C NMR (151 MHz, DMSO) δ 165.49, 165.43, 162.05, 161.62, 158.98, 158.55, 157.83, 157.36, 156.24, 153.09, 135.07, 134.95, 126.28, 125.08, 121.84, 121.29, 121.24, 114.76, 114.61, 112.58, 98.30, 96.81, 27.41, 27.14, 16.95, 10.55. HRMS (ESI) for C_20_H_20_FN_5_O_2_ [M-H]^−^: calcd, 380.1523, found, 380.1515. HPLC: 99.361%.

2-(methylamino)-4-((4-(*N*,*N*-dimethylsulfamoyl)phenyl)amino)-*N*-(3-hydroxy-2,6-dimethylphenyl)pyrimidine-5-carboxamide (**MS20**):

White solid; yield: 52.1%; mp: 258.5–259.4 °C; ^1^H NMR (600 MHz, DMSO-*d*_6_) δ 11.85 (s, 0.6H), 11.67 (s, 0.4H), 9.70 (s, 0.4H), 9.66 (s, 0.6H), 9.20 − 9.18 (m, 1H), 8.94 (s, 0.4H), 8.87 (s, 0.6H), 8.04 (d, *J* = 8.9 Hz, 2H), 7.73 − 7.67 (m, 2H), 7.63 (d, *J* = 8.0 Hz, 1H), 6.90 (d, *J* = 8.2 Hz, 1H), 6.71 (d, *J* = 8.2 Hz, 1H), 2.96 − 2.86 (m, 3H), 2.59 (s, 6H), 2.08 (s, 3H), 2.00 (s, 3H). ^13^C NMR (151 MHz, DMSO) δ 165.33, 165.25, 162.02, 161.49, 159.08, 158.53, 157.69, 153.10, 142.88, 142.76, 134.78, 134.71, 128.23, 128.02, 126.60, 126.44, 126.33, 125.04, 121.81, 119.02, 112.71, 112.67, 98.80, 97.40, 37.04, 27.57, 27.15, 16.93, 10.54. HRMS (ESI) for C_22_H_26_N_6_O_4_S [M-H]^−^: calcd, 469.1658, found, 469.1652. HPLC: 95.598%.

2-(methylamino)-4-((4-nitrophenyl)amino)-*N*-(3-hydroxy-2,6-dimethylphenyl)pyrimidine-5-carboxamide (**MS21**):

Yellow solid; yield: 63.2%; mp: 309.2–310.1 °C; ^1^H NMR (600 MHz, DMSO-*d*_6_) δ 12.03 (s, 0.6H), 11.89 (s, 0.4H), 9.74 (s, 0.4H), 9.71 (s, 0.6H), 9.22 − 9.18 (m, 1H), 8.98 (s, 0.4H), 8.91 (s, 0.6H), 8.24 − 8.12 (m, 2H), 8.08 − 8.00 (m, 2H), 7.82 − 7.70 (m, 1H), 6.91 (d, *J* = 8.2 Hz, 1H), 6.71 (d, *J* = 8.3 Hz, 1H), 2.95 − 2.90 (m, 3H), 2.08 (s, 3H), 2.00 (s, 3H). ^13^C NMR (151 MHz, DMSO) δ 165.23, 165.16, 161.99, 161.44, 158.97, 158.40, 157.85, 153.11, 145.07, 144.91, 140.63, 140.55, 134.71, 134.64, 126.35, 125.02, 124.42, 124.22, 121.80, 118.98, 118.83, 112.75, 112.70, 98.96, 97.61, 27.59, 27.14, 16.93, 10.54. HRMS (ESI) for C_20_H_20_N_6_O_4_ [M+Na]^+^: calcd, 431.1444, found, 431.1441. HPLC: 99.404%.

2-(methylamino)-4-((4-methoxyphenyl)amino)-*N*-(3-hydroxy-2,6-dimethylphenyl)pyrimidine-5-carboxamide (**MS22**):

White solid; yield: 61.8%; mp: 265.0–266.1 °C; ^1^H NMR (600 MHz, DMSO-*d*_6_) δ 11.23 (s, 0.6H), 11.02 (s, 0.4H), 9.56 (s, 0.4H), 9.52 (s, 0.6H), 9.16 (s, 1H), 8.84 (s, 0.4H), 8.77 (s, 0.6H), 7.69 − 7.60 (m, 2H), 7.47 − 7.42 (m, 0.6H), 7.31 (s, 0.4H), 6.93 − 6.85 (m, 3H), 6.70 (d, *J* = 8.2 Hz, 1H), 3.73 (s, 3H), 2.86 (d, *J* = 4.7 Hz, 3H), 2.07 (s, 3H), 1.99 (s, 3H). ^13^C NMR (151 MHz, DMSO-*d*_6_) δ 165.33, 165.25, 162.02, 161.49, 159.08, 158.53, 157.69, 153.10, 142.88, 142.76, 134.78, 134.71, 128.23, 128.02, 126.60, 126.44, 126.33, 125.04, 121.81, 119.02, 118.99, 112.71, 112.67, 98.80, 97.40, 37.04, 27.57, 27.15, 16.93, 10.54. HRMS (ESI) for C_21_H_23_N_5_O_3_ [M-H]^−^: calcd, 392.1723, found, 392.1722. HPLC: 99.772%.

2-(ethylamino)-4-((4-fluorophenyl)amino)-*N*-(3-hydroxy-2,6-dimethylphenyl)pyrimidine-5-carboxamide (**MS23**):

White solid; yield: 73.2%; mp: 267.1–268.5 °C; ^1^H NMR (600 MHz, DMSO-*d*_6_) δ 11.37 (s, 0.6H), 11.21 (s, 0.4H), 9.59 (s, 0.4H), 9.56 (s, 0.6H), 9.17 (s, 1H), 8.85 (s, 0.4H), 8.81 (s, 0.6H), 7.81 − 7.70 (m, 2H), 7.63 − 7.60 (m, 0.6H), 7.51 − 7.48 (m, 0.4H), 7.20 − 7.10 (m, 2H), 6.90 (d, *J* = 8.2 Hz, 1H), 6.70 (d, *J* = 8.2 Hz, 1H), 3.34 − 3.29 (m, 2H), 2.07 (s, 3H), 2.00 (s, 3H), 1.19 − 1.12 (m, 3H). ^13^C NMR (151 MHz, DMSO-*d*_6_) δ 165.46, 161.35, 161.05, 158.98, 158.65, 157.85, 157.41, 157.33, 156.26, 153.09, 135.10, 134.93, 126.29, 125.09, 121.85, 121.29, 121.24, 114.75, 114.60, 112.60, 98.23, 96.93, 35.11, 34.76, 16.95, 14.33, 13.88, 10.55. HRMS (ESI) for C_21_H_22_FN_5_O_2_ [M-H]^−^: calcd, 394.1680, found, 394.1675. HPLC: 99.860%.

2-(ethylamino)-4-((4-(*N*,*N*-dimethylsulfamoyl)phenyl)amino)-*N*-(3-hydroxy-2,6-dimethylphenyl)pyrimidine-5-carboxamide (**MS24**):

White solid; yield: 57.0%; mp: 306.1–307.0 °C; ^1^H NMR (600 MHz, DMSO-*d*_6_) δ 11.84 (s, 0.6H), 11.68 (s, 0.4H), 9.69 (s, 0.4H), 9.66 (s, 0.6H), 9.18 (s, 1H), 8.92 (s, 0.4H), 8.88 (s, 0.6H), 8.06 (d, *J* = 8.5 Hz, 0.8H), 8.01 (d, *J* = 8.4 Hz, 1.2H), 7.79 (t, *J* = 5.7 Hz, 1H), 7.69 (d, *J* = 8.5 Hz, 1.2H), 7.63 (d, *J* = 8.4 Hz, 0.8H), 6.90 (d, *J* = 8.2 Hz, 1H), 6.71 (d, *J* = 8.2 Hz, 1H), 3.40 (p, *J* = 7.0 Hz, 2H), 2.59 (s, 6H), 2.08 (s, 3H), 2.00 (s, 3H), 1.25 − 1.15 (m, 3H). ^13^C NMR (151 MHz, DMSO-*d*_6_) δ 165.31, 165.23, 161.32, 160.92, 159.06, 158.62, 157.74, 153.10, 142.89, 142.76, 134.79, 134.73, 128.21, 128.01, 126.60, 126.39, 126.33, 125.05, 121.81, 118.98, 118.93, 112.70, 112.67, 98.73, 97.49, 37.04, 35.31, 34.81, 16.93, 14.25, 13.80, 10.53. HRMS (ESI) for C_23_H_28_N_6_O_4_S [M-H]^−^: calcd, 483.1815, found, 483.1805. HPLC: 96.935%.

### 2.3. PKMYT1 Kinase Binding Affinity Assay

The inhibitory activity against PKMYT1 was determined using the LanthaScreen^TM^ Eu kinase binding assay platform from Thermo Fisher Scientific (Waltham, MA, USA). Test compounds were first dissolved in DMSO to prepare 4 mM stock solutions and then serially diluted with 1× assay buffer to the desired concentrations. In a 384-well white microplate, 4 μL of diluted compound solution and 8 μL of kinase reaction mixture (containing 2.5 nM PKMYT1 kinase and 2 nM Eu-labeled anti-GST antibody) were added sequentially, and the plate was centrifuged at 1000 rpm for 1 min. Subsequently, 4 μL of Tracer178 (final concentration 2.5 nM) was added to initiate the competitive binding reaction. After the final addition, the plate was centrifuged again at 1000 rpm for 1 min and incubated at 25 °C in the dark for 1 h to allow binding to reach equilibrium. Fluorescence signals were recorded at 665 nm and 615 nm in time-resolved fluorescence resonance energy transfer (TR-FRET) mode. All dose–response curves and IC_50_ values were fitted using GraphPad Prism 8 software.

### 2.4. Cell Anti-Proliferative Assay

OVCAR3 (from Procell, CL-0178) and HCC1569 (from BNCC, BNCC341698) cells in the logarithmic growth phase were plated into 96-well plates at an initial density of 2000 cells per well. Twenty-four hours post-seeding, the cells were treated with various concentrations of the test compounds and incubated at 37 °C in a 5% CO_2_ atmosphere for 5 days. The final DMSO concentration was maintained at <0.1% (*v*/*v*) and kept constant across all treatment groups. Then, CCK-8 reagent was added at 10% of the well volume, and incubation was continued for an additional 1–2 h. Absorption signals were recorded at 450 nm, and data were generated by GraphPad Prism 8.

### 2.5. Cell Selectivity Assay Procedures

A549 (from Procell, CL-0016) and HEK293T (from Procell, CL-0005) cells in the logarithmic growth phase were plated into 96-well plates at an initial density of 1500 and 2000 cells per well, respectively. Twenty-four hours post-seeding, the cells were treated with various concentrations of the test compounds and incubated at 37 °C in a 5% CO_2_ atmosphere for 5 days. The final DMSO concentration was maintained at <0.1% (*v*/*v*) and kept constant across all treatment groups. Then, CCK-8 reagent was added at 10% of the well volume, and incubation was continued for an additional 1–2 h. Absorption signals were recorded at 450 nm, and data were generated by GraphPad Prism 8.

### 2.6. Human Liver Microsomal Stability Assay

Metabolic stability was evaluated exclusively using human liver microsomes. The final substrate concentration was 1 μM, and the microsomal protein concentration was adjusted to 0.5 mg/mL. For LC-MS/MS separation, mobile phase A consisted of 0.1% formic acid in water, and mobile phase B was acetonitrile.

Liver microsomes were thawed by pre-incubation in a 37 °C water-bath shaker for 5 min. A 2 mM NADPH solution was prepared by dissolving NADPH in an appropriate volume of magnesium chloride solution. An incubation mixture containing liver microsomes and substrate was prepared and dispensed into tubes (180 μL per tube). For the 0 min samples, 30 μL of the microsome–substrate mixture was taken from the test compound and positive control groups, and immediately mixed with 300 μL of internal standard precipitant followed by 30 μL of NADPH solution. For the test compound group, the reaction was initiated by the addition of 150 μL of NADPH solution (the negative control group received 50 μL of magnesium chloride solution instead), and the mixtures were incubated at 37 °C in a water bath. At time points of 5, 15, 30, and 60 min (negative control: 60 min only), 60 μL aliquots were withdrawn and added to 300 μL of precipitant containing internal standard. The positive control group was initially supplemented with 90 μL of NADPH solution and, after incubation for 5 and 15 min, quenched in the same manner. A neat sample was prepared by mixing 297 μL of water, 1500 μL of internal standard working solution, and 3 μL of test compound working solution. All samples were vortexed and centrifuged, and 150 μL of each supernatant was diluted with 150 μL of water, vortex-mixed, and subjected to LC-MS/MS analysis.

### 2.7. Cell Cloning Assay

OVCAR3 and HCC1569 in the logarithmic growth phase were plated into 6-well plates at an initial density of 5000 and 20,000 cells per well, respectively. Twenty-four hours post-seeding, the cells were treated with various concentrations of the test compounds and incubated at 37 °C in a 5% CO_2_ atmosphere for 14 days. No additional compounds were added or medium refreshment performed throughout the 14-day treatment period. The final DMSO concentration was maintained at <0.1% (*v*/*v*) and kept constant across all treatment groups. Cells were rinsed with PBS once following medium aspiration, then immobilized in 4% paraformaldehyde solution at ambient temperature over a 1 h period. The fixation liquid was aspirated off, and another PBS rinse was carried out. Samples were stained with crystal violet working solution; the stain was drained after 10 min, and the wells underwent three sequential PBS washes. Microscopic images were captured, cell quantification was completed via ImageJ software, and all statistical analyses were processed using GraphPad Prism 8.

### 2.8. Cell Cycle Assay Procedures

HCC1569 cells in the logarithmic growth phase were plated into 6-well plates at an initial density of 200,000 cells per well. Twenty-four hours post-seeding, the cells were treated with various concentrations of the test compounds and incubated at 37 °C in a 5% CO_2_ atmosphere for 5 days. The final DMSO concentration was maintained at <0.1% (*v*/*v*) and kept constant across all treatment groups. The culture medium was collected, and the adherent cells were detached using trypsin without EDTA, followed by another collection. After centrifugation, the cells were washed once with PBS, and the PBS was discarded. The cells were then resuspended in 70% ethanol and incubated at 4 °C for 1 d. After centrifugation, the supernatant was removed, PBS was added for washing, and following another centrifugation, the PBS was discarded. Propidium iodide (PI) and RNase A were added according to the Beyotime kit protocol, and the mixture was incubated at 37 °C for 0.5 h in the dark. Flow cytometry analysis was then performed using a BD Accuri^TM^ C6 Plus Flow Cytometer (BD Biosciences, San Jose, CA, USA), and data were analyzed with FlowJo software. All statistical analyses were carried out using Prism 8.

### 2.9. Molecular Docking Procedures

Molecular docking was performed using the Glide module following a standardized, rigorously validated workflow. The crystal structure of the target protein (PDB ID: 8D6E) was retrieved from the RCSB Protein Data Bank (https://www.rcsb.org); its co-crystallized ligand is **RP-6306**. The docking procedures comprised five sequential, methodologically coherent steps: (1) Protein preparation—Conducted via the Protein Preparation WorkFlow, this involved removal of co-crystallized ligands and all water molecules, addition of missing hydrogen atoms and side-chain conformations using the OPLS4 force field, and subsequent energy minimization with an RMSD convergence threshold of 0.30 Å. (2) Ligand preparation—Energy-minimized three-dimensional conformations were generated using the LigPrep tool; Epik was configured to produce only a single, most significant conformation at physiological pH 7.4. (3) Grid generation—This centered on the native ligand-binding site defined by the co-crystallized ligand in 8D6E, with grid center coordinates (X, Y, Z) of 19.94, 10.23, and 7.42 Å, and a box size of 22.73 Å, to ensure biologically relevant sampling. (4) Docking execution—Glide was employed in Standard Precision (SP) mode with flexible docking, default constraint settings, a van der Waals radius scaling factor of 0.80, and a partial charge cutoff of 0.15 [29]. (5) Structural analysis and visualization—This was conducted in PyMOL 3.0.

### 2.10. Molecular Dynamics Simulation Procedures 

Molecular dynamics simulations were conducted using the Desmond module of Schrödinger. The system employed the OPLS4 force field together with the SPC explicit water model. The solute was placed in a periodic orthogonal water box, with the shortest distance from any solute atom to the box boundary set to 10 Å × 10 Å × 10 Å. A suitable number of Na^+^ and Cl^−^ ions were added to neutralize the net charge of the system while maintaining the NaCl concentration at 0.15 M to simulate near-physiological ionic strength. Following the default relaxation and equilibration protocols of Desmond, a pre-equilibrium process lasting 0.1 ns was performed on the system, and a 300 ns production run was performed in the NPT ensemble. The integration time step was set to 2 fs. The temperature was maintained at 300 K, and the pressure was held at 1.01325 bar [30]. The current geometric criteria for protein–ligand H-bonds are: a donor–acceptor distance (D⋯A) ≤ 2.5 Å; a donor angle (D—H⋯A) ≥ 120°; and an acceptor angle (H⋯A—X) ≥ 90°, where X is the atom bonded to the acceptor. The repeated simulations were performed using different random seeds for the initial velocities.

### 2.11. Computational Alanine Scanning Mutagenesis Procedures

Computational alanine scanning was performed via the built-in Residue Scanning Calculations module in Schrödinger Maestro. All calculations were performed using only the final snapshot extracted from the equilibrium phase of a single representative 300 ns molecular dynamics trajectory, without averaging over the full conformational ensemble of that trajectory. The entire computational workflow strictly adopted the factory default parameters of the Residue Scanning module, including its internal force field, solvent dielectric parameters, and restrained energy minimization protocol. Each amino acid residue within the binding pocket was automatically mutated to alanine one by one. The relative binding free energy difference ΔΔG between mutant and wild-type complexes was exported to evaluate the interaction strength between each residue and the ligand.

### 2.12. MM-GBSA Binding Energy Calculations

Snapshots extracted every 0.3 ns from the MD trajectories were processed using the thermal_mmgbsa.py script, and the resulting data were output and visualized with Prism 8.

### 2.13. Statistical Analysis

All statistical analyses were performed using GraphPad Prism 8. Kinase activity data were fitted to a log(inhibitor) vs. response–variable slope (four-parameter) model, while cellular activity data were fitted to a log(inhibitor) vs. normalized response–variable slope model. For colony formation and cell cycle distribution analyses, data normality was first assessed using the Shapiro–Wilk test. As the data met the normality criteria, statistical significance between the control and experimental groups was subsequently evaluated using one-way ANOVA.

## 3. Results

### 3.1. Chemistry

**Figure 2:**
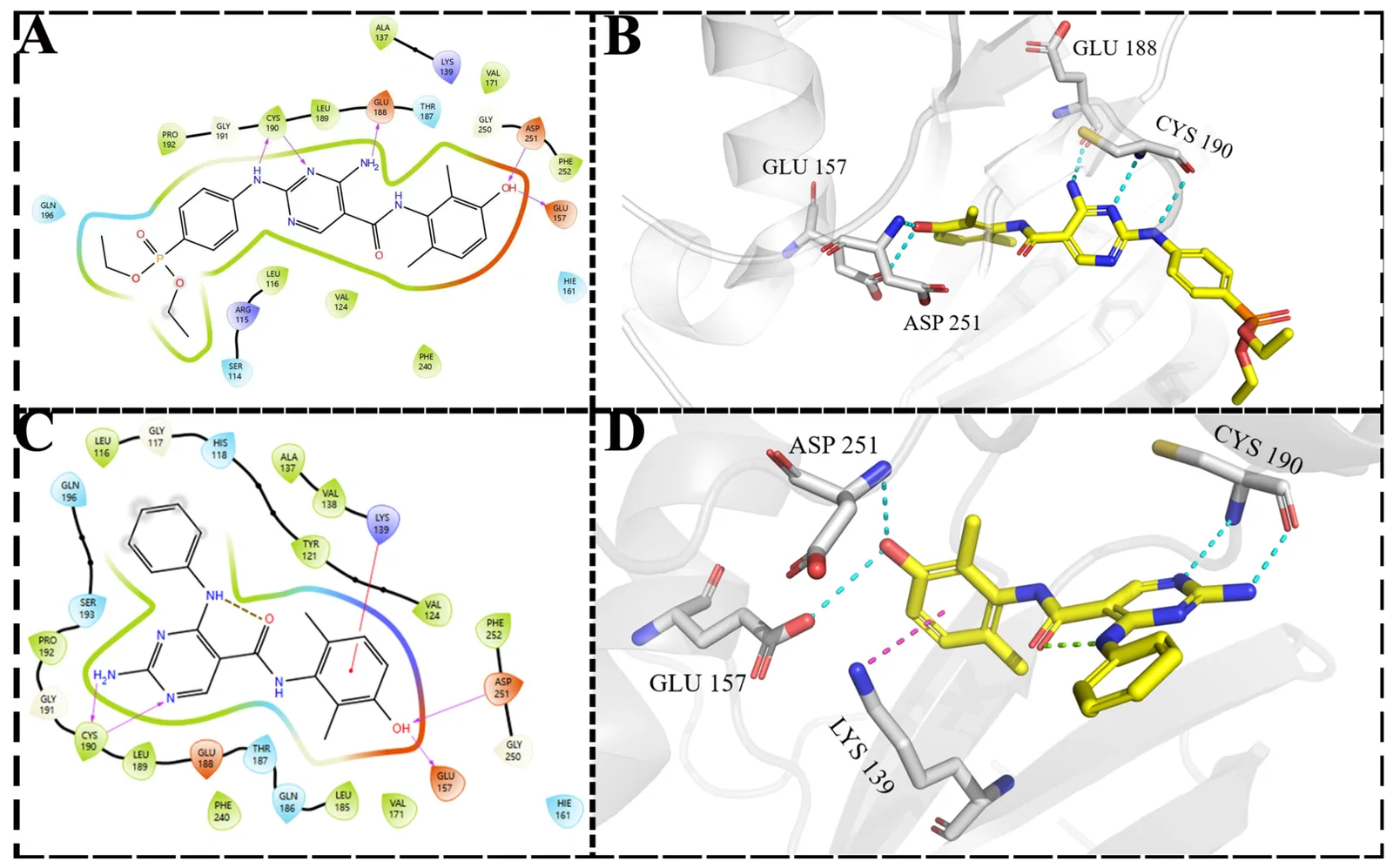
Binding modes of **MY-14** and **MS1** in complex with the PKMYT1 protein (PDB ID: 8D6E). 2D (**A**) and 3D (**B**) diagrams of compound **MY-14**’s interaction with PKMYT1. 2D (**C**) and 3D (**D**) diagrams of compound **MS1**’s interaction with PKMYT1.

In previous studies, our research group identified a highly active compound, **MY-14** [31]. Through molecular dynamics (MD) simulations, we found that the preferred conformation of this structure did not align with our expectations; specifically, the carbonyl group in the amide did not form an intramolecular hydrogen bond with the primary amine at the 4-position of the pyrimidine. We considered that the lack of intramolecular hydrogen bonds could have significant implications for the metabolism of the amide in vivo. This issue was already observed in a prior study (**A30**) [32]. Therefore, we attempted to dissect the molecular binding mode of **MY-14**. The amino group at the 2-position in the hinge region and the nitrogen atom of the pyrimidine could interact with CYS190, while the primary amine at the 4-position could form a hydrogen bond with GLU188. However, this interaction would disrupt the low-energy conformation of the molecule, placing the amide carbonyl and the pyrimidine 4-position primary amine on opposite sides of the scaffold center, as shown in Figure 2, thereby preventing the formation of the intramolecular hydrogen bond. Consequently, we employed a pharmacophore migration strategy, transferring the substituent from the 2-position amino group to the 4-position to promote the formation of the intramolecular hydrogen bond. At the same time, the exposed primary amine at the 2-position would facilitate hydrogen bonding interactions with CYS190. Ultimately, we designed the compound **MS1**.

**Scheme 1:**
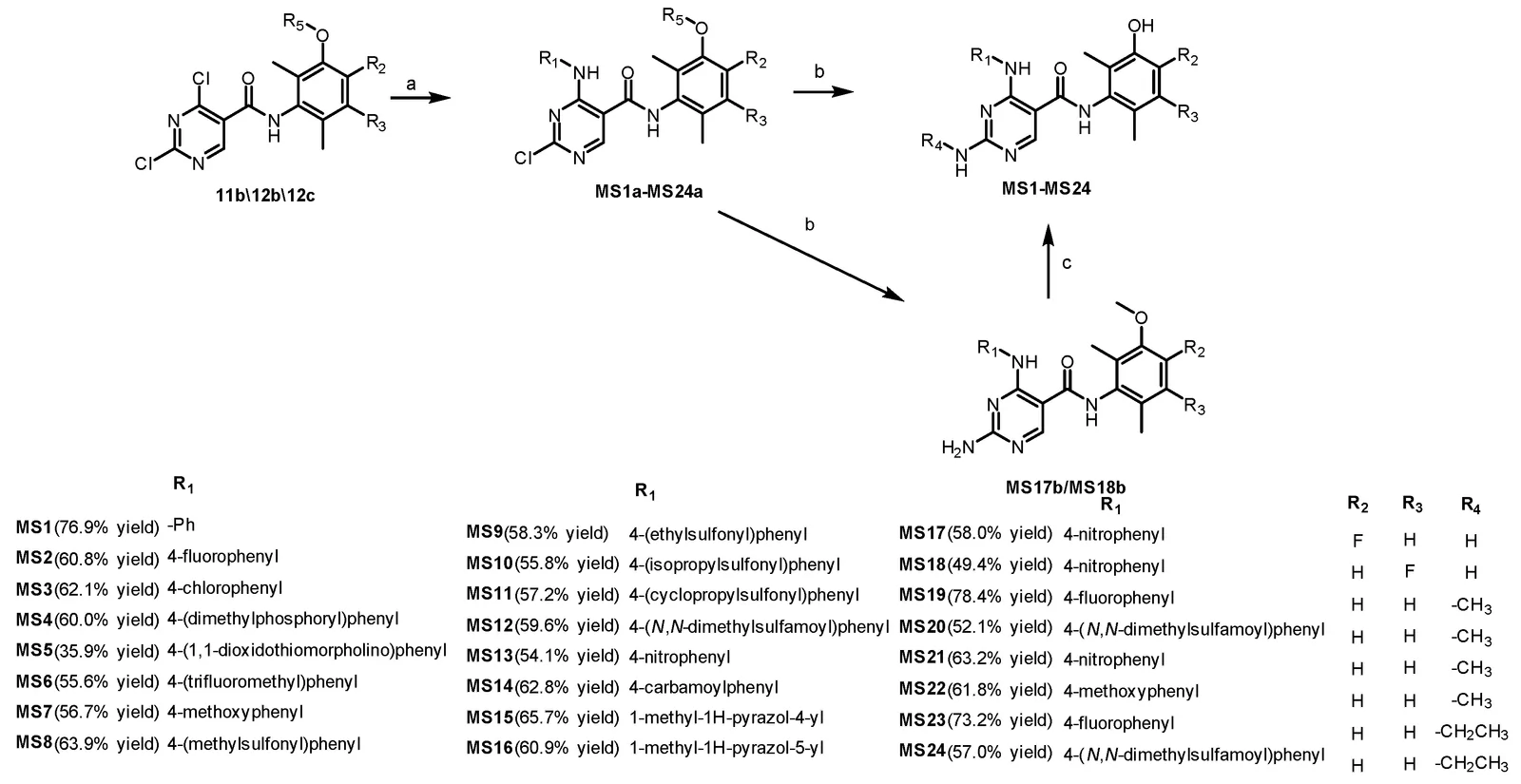
Reagents and conditions: (a) substituted aniline, DIPEA, *n*-BuOH, 100 °C, 4 h, 50.5–87.3%; (b) 25% aq NH_3_, MeNH_2_, or EtNH_2_, *i*-PrOH, 120 °C, 3 h, 80.4–92.3%; (c) 2 M BBr_3_, DCM (dry), −40 °C, 3 h, 35.9–76.9%.

The synthesis of key intermediates **11b**, **12b**, and **12c** has been previously reported by Zhang et al. [32]. Building upon this foundation, we further modified this structure. The synthetic route for compounds **MS1**–**MS24** is outlined in Scheme 1. The substitution reactions of **11b**, **12b**, and **12c** with various aniline derivatives yielded **MS1a**–**MS24a**. When R_5_ is TBS, the reaction with ammonia, methylamine, and ethylamine leads to the final products **MS1**–**MS16** and **MS19**–**MS24**. When R_5_ is a methyl group, the products **MS17b**–**MS18b** are obtained, which require an additional demethylation step to synthesize the final products **MS17**–**MS18**.

### 3.2. SAR of PKMYT1 Inhibitors

The preliminary exploration of structural optimization will focus on modifying the R_1_ group exposed in the solvent region, as shown in Table 1. Initially, halogen substitution at the 4-position of the phenyl ring was conducted. The introduction of fluorine resulted in a further increase in the kinase binding affinity of compound **MS2** (IC_50_ = 3.01 nM), whereas the introduction of chlorine led to a mild reduction in the kinase binding affinity of compound **MS3** (IC_50_ = 5.96 nM). This may indicate that the binding pocket is extremely sensitive to steric hindrance, and the strong electron-withdrawing effect or specific polar interactions may be key to enhancing activity. To test this inference, we introduced large sterically hindered groups such as phosphonyl **MS4** (IC_50_ = 10.07 nM), thio-morpholine-1,1-dioxide-4-yl **MS5** (IC_50_ = 10.66 nM), and trifluoromethyl **MS6** (IC_50_ = 13.26 nM), all of which markedly reduced kinase binding affinity. Subsequently, we investigated the introduction of the electron-donating methoxy group, which also resulted in a slight reduction in kinase binding affinity. These results may indicate that the introduction of strong electron-withdrawing groups is important for enhancing kinase binding affinity during compound modification.

To enhance kinase binding affinity, we introduced strong electron-withdrawing groups for investigation and designed and synthesized compounds **MS8**–**MS14**, as shown in Table 1. We first examined the sulfonyl group and its fragments, among which the cyclopropyl compound **MS11** (IC_50_ = 2.51 nM) exhibited superior overall activity compared to linear alkyl groups. This may be attributed to the unique rigid and planar structure of the cyclopropyl group, which can effectively fit into the binding pocket. Building on this, we introduced lower-volume electron-withdrawing groups such as nitro and formamide groups that can restrict conformation. The nitro-substituted compound **MS13** (IC_50_ = 0.86 nM) showed a significant increase in kinase binding affinity, comparable to the positive control **RP-6306** (IC_50_ = 0.74 nM), possibly indicating the crucial role of the strong electron-withdrawing effect of the nitro group.

To compare the effects of different heterocyclic ring structures, the original side-chain phenyl ring was replaced with two isomeric fragments, 1-methyl-5-pyrazole and 1-methyl-4-pyrazole, leading to the design and synthesis of the regioisomers **MS15** and **MS16**. As shown in Table 1, both exhibited varying degrees of reduced kinase binding affinity. Therefore, subsequent structural modifications will retain the characteristics of the phenyl ring.

To further enhance stability, we anticipate introducing fluorine atoms into the R_2_ and R_3_ regions of the phenol structure, designing and synthesizing compounds **MS17**–**MS18**, as shown in Table 2. A striking decrease in kinase binding affinity was observed when fluorine occupied the ortho position relative to the hydroxyl group. In contrast, the meta-substituted **MS18** displayed slightly better activity than **MS17**; however, its potency remained nearly eightfold lower than that of **MS13** (IC50 = 0.86 nM).

To investigate the structure–activity relationship of the amino substitution pattern at the 2-position of pyrimidine, we introduced methyl and ethyl alkane fragments to examine spatial size. Compounds **MS19**–**MS24** were synthesized, as shown in Table 3. When a methyl group was introduced, the kinase binding affinity of the compounds was not maintained. However, with the increase in the alkane chain, a marked reduction in kinase binding affinity was observed upon the introduction of the ethyl substitution. This may be due to the introduction of these substituents causing collisions with the protein cavity, altering the binding mode of the compounds with amino acid residues, leading to a decline in kinase binding affinity. Therefore, we retained the amino group at the 2-position of pyrimidine and continued testing.

### 3.3. Cell Anti-Proliferation Activity Assay

Based on the initial screening that identified compounds with kinase binding affinity less than 10 nM, we further evaluated their anti-proliferative activity against the *CCNE1*-amplified ovarian cancer cell line OVCAR3. The test results are shown in Table 4. Compounds **MS2**, **MS7**, **MS8**, **MS12**, **MS13**, **MS16**, **MS17**, **MS18**, **MS20**, and **MS22** exhibited superior activity and were further assessed for their anti-proliferative effects on the *CCNE1*-amplified breast cancer cell line HCC1569, as shown in Table 5. **MS13** demonstrated potent anti-proliferative activity against both OVCAR3 and HCC1569 cells, with IC_50_ values of 1.52 μM and 0.66 μM, respectively.

### 3.4. Cell Selectivity Assays

Given that the nitro group is the preferred compound due to its optimal use of kinase binding affinity and cellular activity, assessing its toxicity is crucial. Therefore, we evaluated the cytotoxicity of **MS13** on A549 lung cancer cells with low expression of *CCNE1* and HEK293T human embryonic kidney cells. As shown in Table 6, the IC_50_ values of **MS13** for A549 and HEK293T are 6.55 μM and 3.10 μM, respectively. **MS13** exhibited preferential activity in selected *CCNE1*-amplified cell lines compared to control cells. However, under the tested conditions, its cell efficacy and selectivity are still inferior to those of **RP-6306**.

### 3.5. Human Liver Microsomal Stability Assays

In vitro metabolic stability is a fundamental prerequisite for achieving and maintaining the therapeutic effects of drugs in vitro. We performed a preliminary human liver microsomal stability assay to characterize the metabolic behavior of **MS13**. As shown in Table 7, only 28.7% of the **MS13** remained after 60 min of incubation. The compound exhibited low intrinsic clearance (CL_int_ = 0.043 mL/min/mg protein), reflecting favorable metabolic stability. Linear regression of the substrate depletion curve yielded an R^2^ of 0.9821, confirming the high reliability of the half-life and intrinsic clearance values calculated for **MS13**. In previous studies, the core scaffold compounds of the **MY-14** class generally suffered from poor stability, with the best compound, **A30**, exhibiting a metabolic half-life of 19.6 min [32]. The compound **MS13** exhibits moderate metabolic stability in human liver microsomes, with an in vitro half-life (t_1/2_) of 32.2 min, which is an improvement compared to **A30**. The improved stability may be attributed to the formation of intramolecular hydrogen bonds, which reduces the metabolic problem of the amide.

### 3.6. Impact of ***MS13*** on Clonogenic Survival

**Figure 3:**
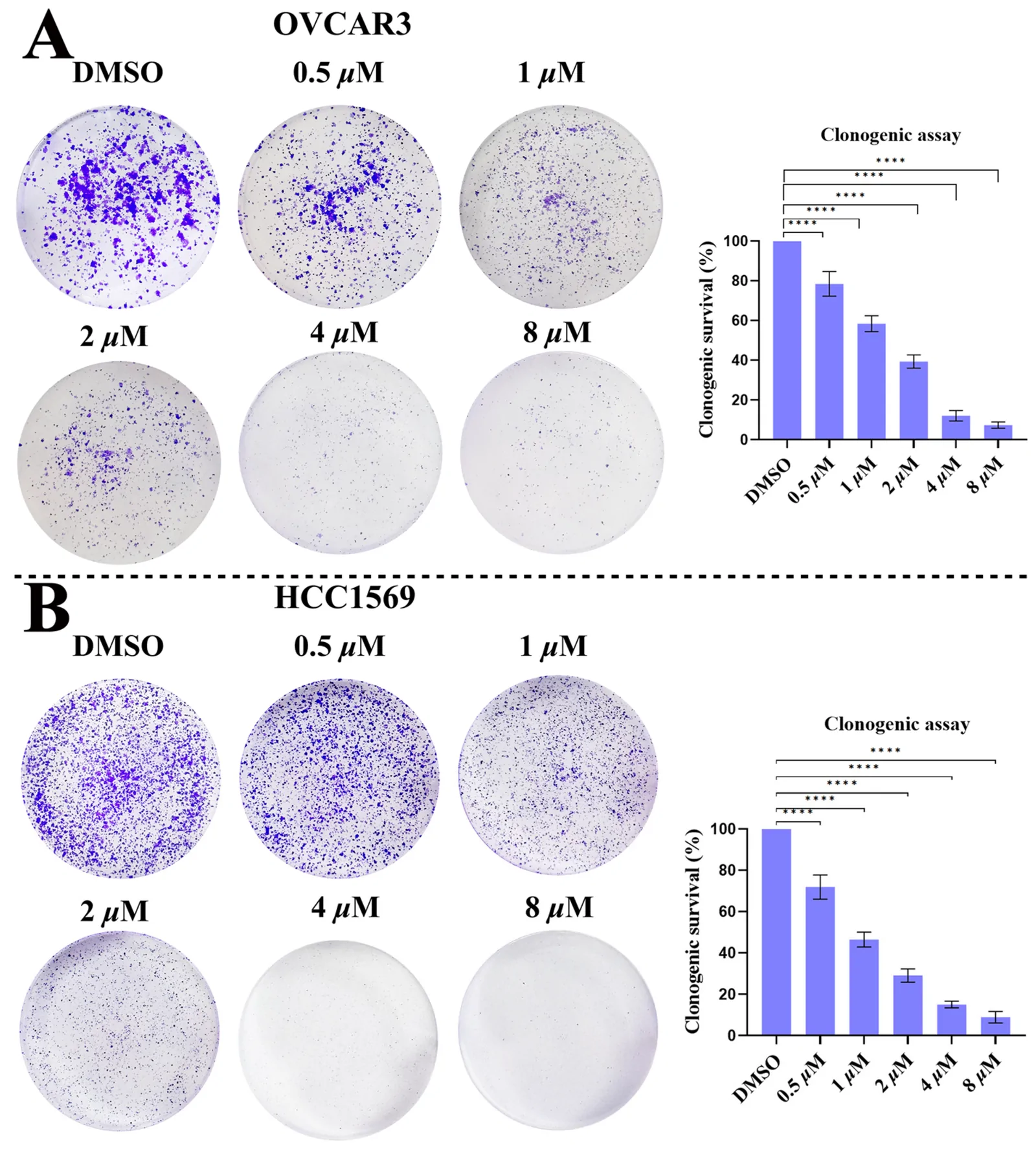
Clonogenic assays were performed to evaluate the clonogenic capacity of *CCNE1*-amplified cells treated with vehicle DMSO and a series of concentrations of **MS13**. (**A**) OVCAR3 cells; (**B**) HCC1569 cells. All experiments were independently repeated at least three times. Error bars represent the mean ± SD. Statistical significance markers are annotated on plots, with comparisons conducted using a one-way ANOVA test. **** *p* < 0.0001.

To deeply assess the effect of **MS13** on the viability of the *CCNE1*- amplified cell lines OVCAR3 and HCC1569, we performed colony formation assays using a series of concentrations of the compound, 8 μM, 4 μM, 2 μM, 1 μM, and 0.5 μM, as shown in Figure 3. The results may indicate that **MS13** inhibits the colony formation of *CCNE1*-amplified cells in a concentration-dependent manner, potentially through the inhibition of PKMYT1.

### 3.7. Cell Cycle Assays

**Figure 4:**
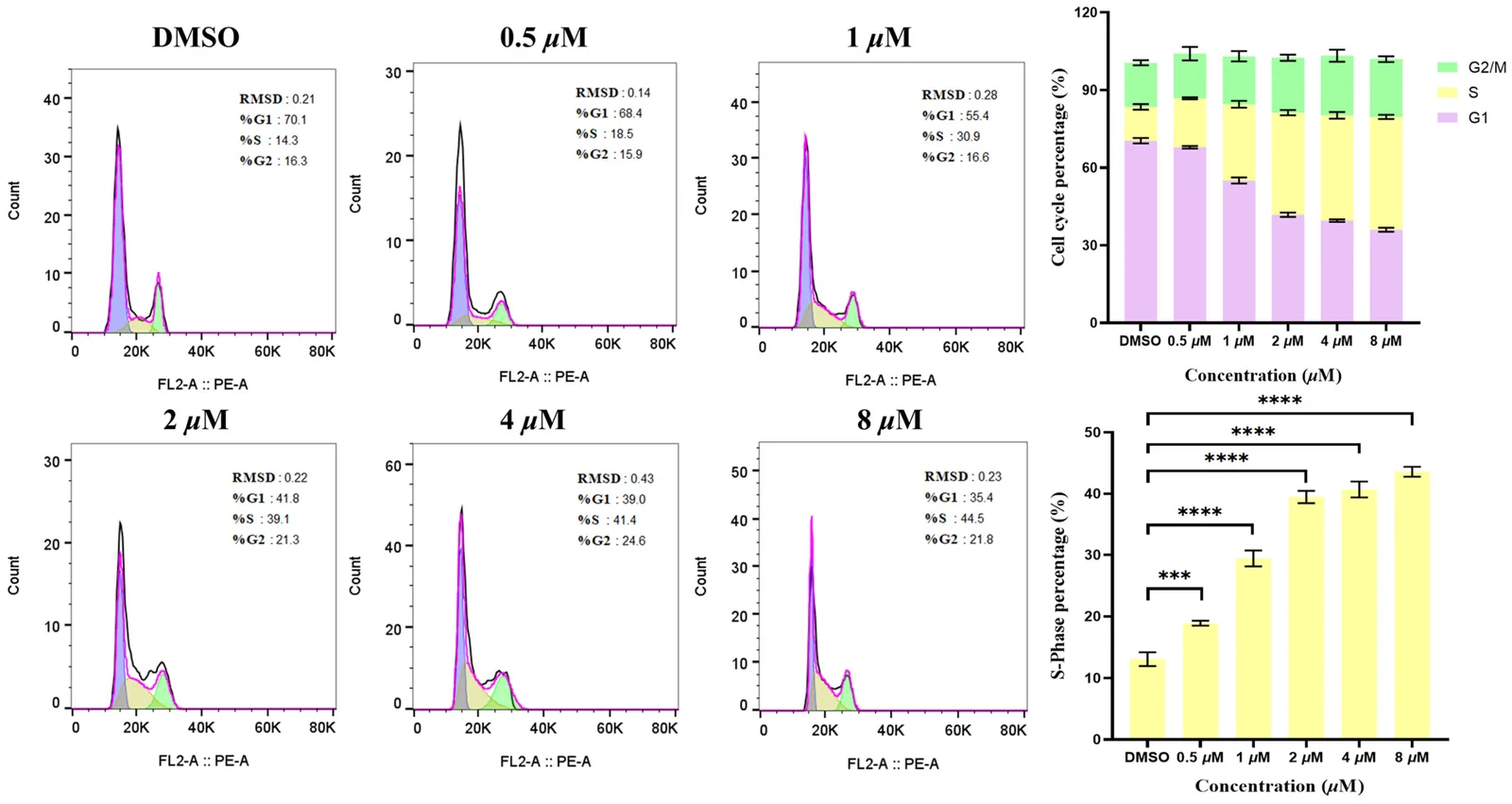
Cell-cycle distribution of **MS13** in HCC1569. Histograms on the left show representative profiles from one of three independent experiments. The stacked bar chart on the top right displays quantitative data (mean ± SD) from three independent biological replicates. The bar chart on the bottom right shows the proportion of S-phase cells at different drug concentrations. Statistical significance markers are annotated on plots, with comparisons conducted using a one-way ANOVA. *** *p* < 0.001, **** *p* < 0.0001.

The impact of compound **MS13** on cell cycle arrest in HCC1569 cells was investigated using flow cytometry, as shown in Figure 4. A concentration-dependent rise in the S-phase cell population was elicited by **MS13**, concomitant with a marked decline in cells residing in the G1 phase. The flow cytometry results suggest that compound **MS13** may mediate S-phase cell cycle arrest through a synthetic lethality effect, likely by targeting the inhibition of PKMYT1, thereby regulating the synthetic lethality partner *CCNE1*. Data are representative of at least three independent experiments.

### 3.8. Molecular Docking

**Figure 5:**
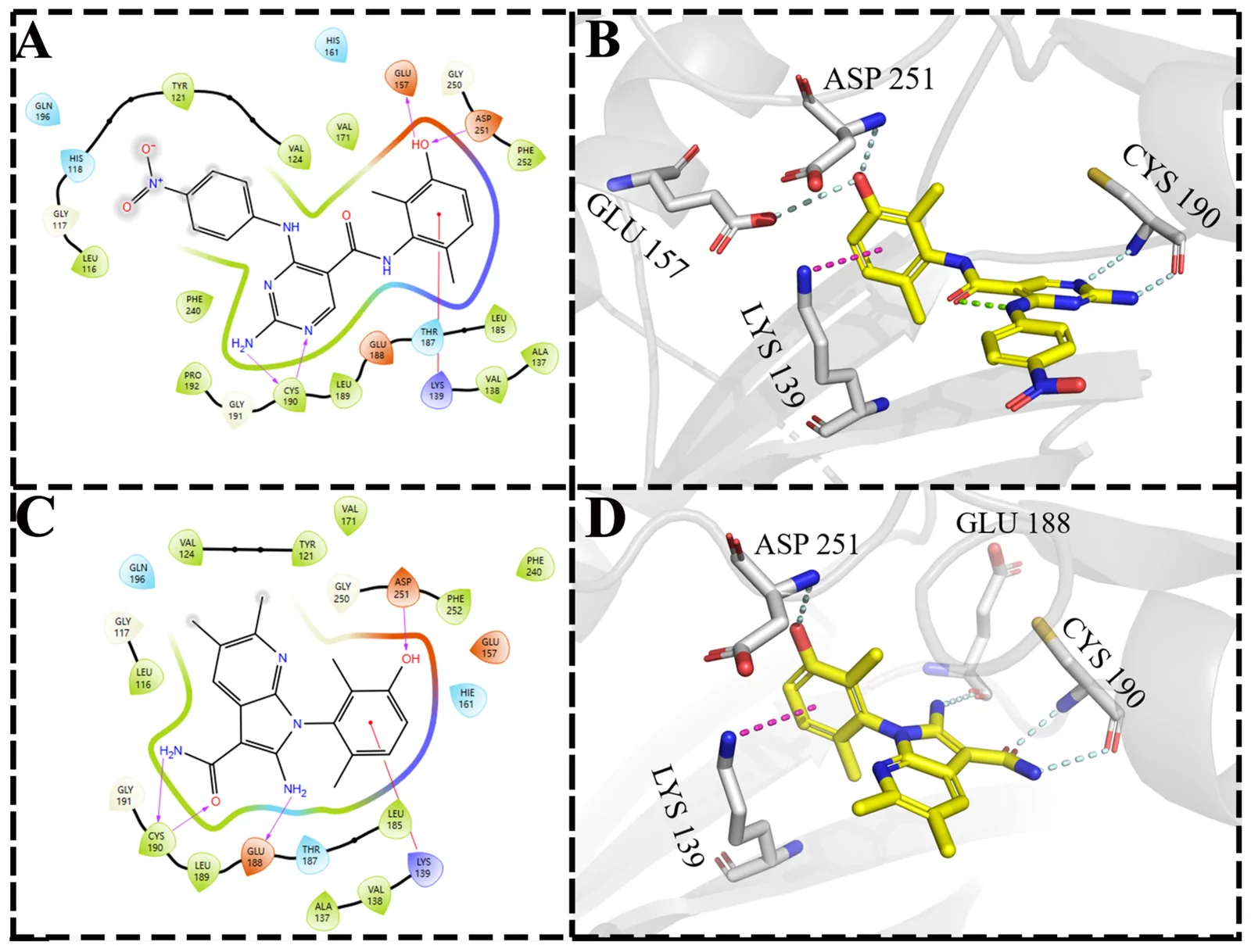
Binding modes of **MS13** and **RP-6306** in complex with the PKMYT1 protein (PDB ID: 8D6E). 2D (**A**) and 3D (**B**) diagrams of compound **MS13**’s interaction with PKMYT1. 2D (**C**) and 3D (**D**) diagrams of compound **RP-6306**’s interaction with PKMYT1.

To analyze the binding mode of **MS13** with PKMYT1 and the role of key residues, we first performed molecular docking, as shown in Figure 5. **MS13** forms hydrogen bond interactions with ASP251 and GLU157 in the DFG motif, and a π–cation interaction with LYS139. In the hinge region, it forms two sets of hydrogen bonds with CYS190. Compared to **RP-6306**, the trisubstituted phenol enhances the anchoring of GLU157 in the DFG region. However, there are important conserved water molecules within the catalytic pocket of the PKMYT1 protein, which molecular docking cannot accurately indicate in terms of its binding mode.

### 3.9. Molecular Dynamics Simulations

**Figure 6:**
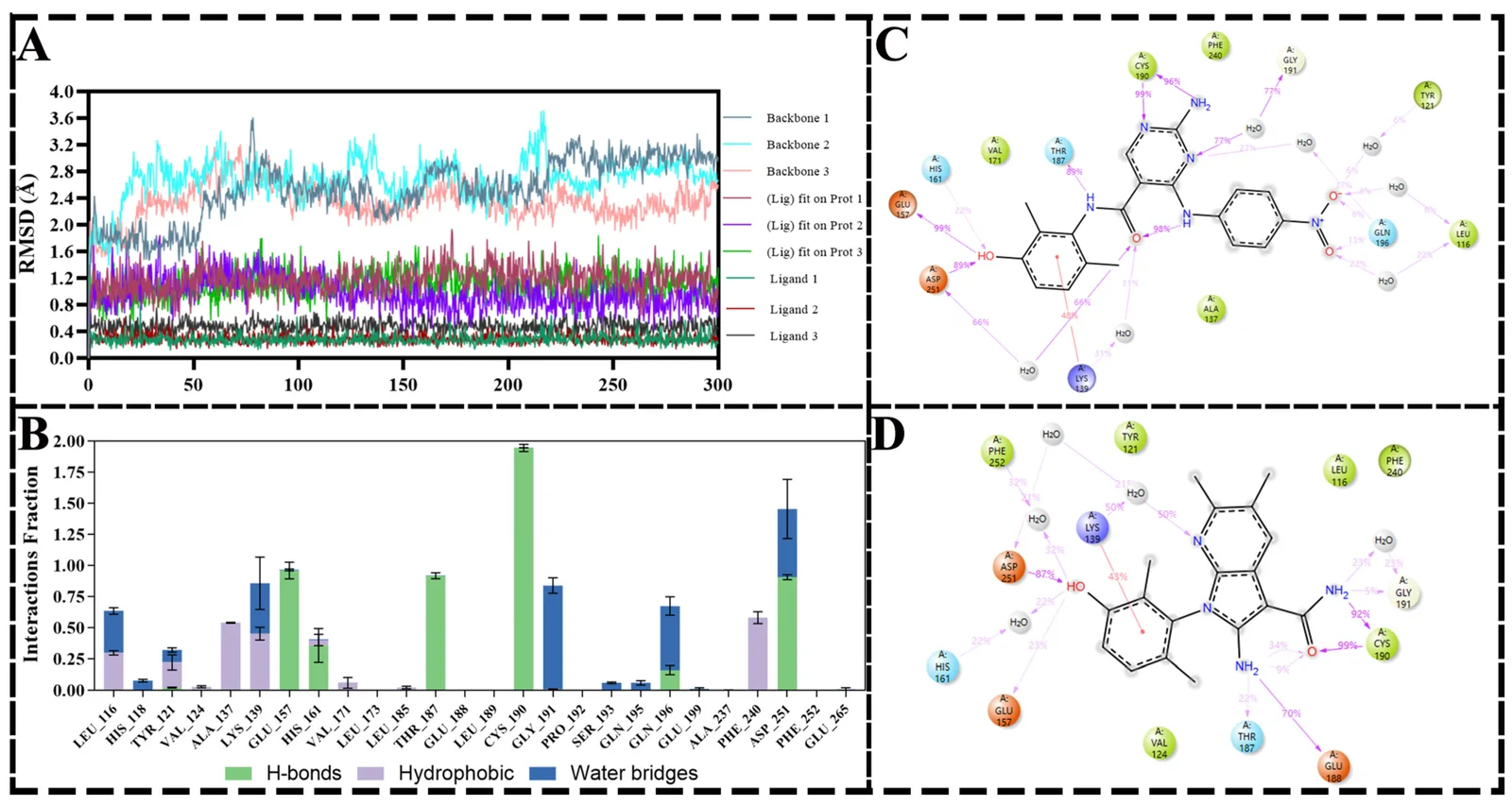
MD simulation of **MS13** over 300 ns. (**A**) RMSD trajectories of the protein–ligand system: from top to bottom, backbone RMSD from three simulations, complex RMSD from three simulations, and ligand RMSD from three simulations. (**B**) Residue interaction profile of **MS13** from three repeated simulations. Data are presented as mean ± standard deviation. (**C**) Two-dimensional interaction contact map of **MS13** from one representative MD simulation. (**D**) Two-dimensional interaction contact map of **RP-6306** from one representative MD simulation. Interactions with a duration exceeding 5% of the simulation time are shown.

To avoid potential false positive results in molecular docking due to the rigidity of proteins and the singular conformation of molecules, we further evaluated the binding mode between **MS13** and the PKMYT1 protein through molecular dynamics simulations. To ensure the reliability of the binding mode, we performed at least three independent molecular dynamics simulations on the **MS13** system. As shown in Figure 6A, the RMSD profiles of the protein–ligand complex were generally consistent across the three simulations, remaining below 2 Å throughout with only minor fluctuations and no significant drift, indicating that the trajectories converged well and the simulation results are valid. **RP-6306** is the co-crystallized ligand of PKMYT1 (PDB ID: 8D6E) studied in this work, and its structure has already been experimentally resolved. Therefore, we performed only a single 300 ns molecular dynamics simulation for this complex, which served to validate the reliability of the computational protocol. As shown in Figure 6D, all key residue interactions observed in the crystal structure were well maintained throughout the simulation.

**Figure 7:**
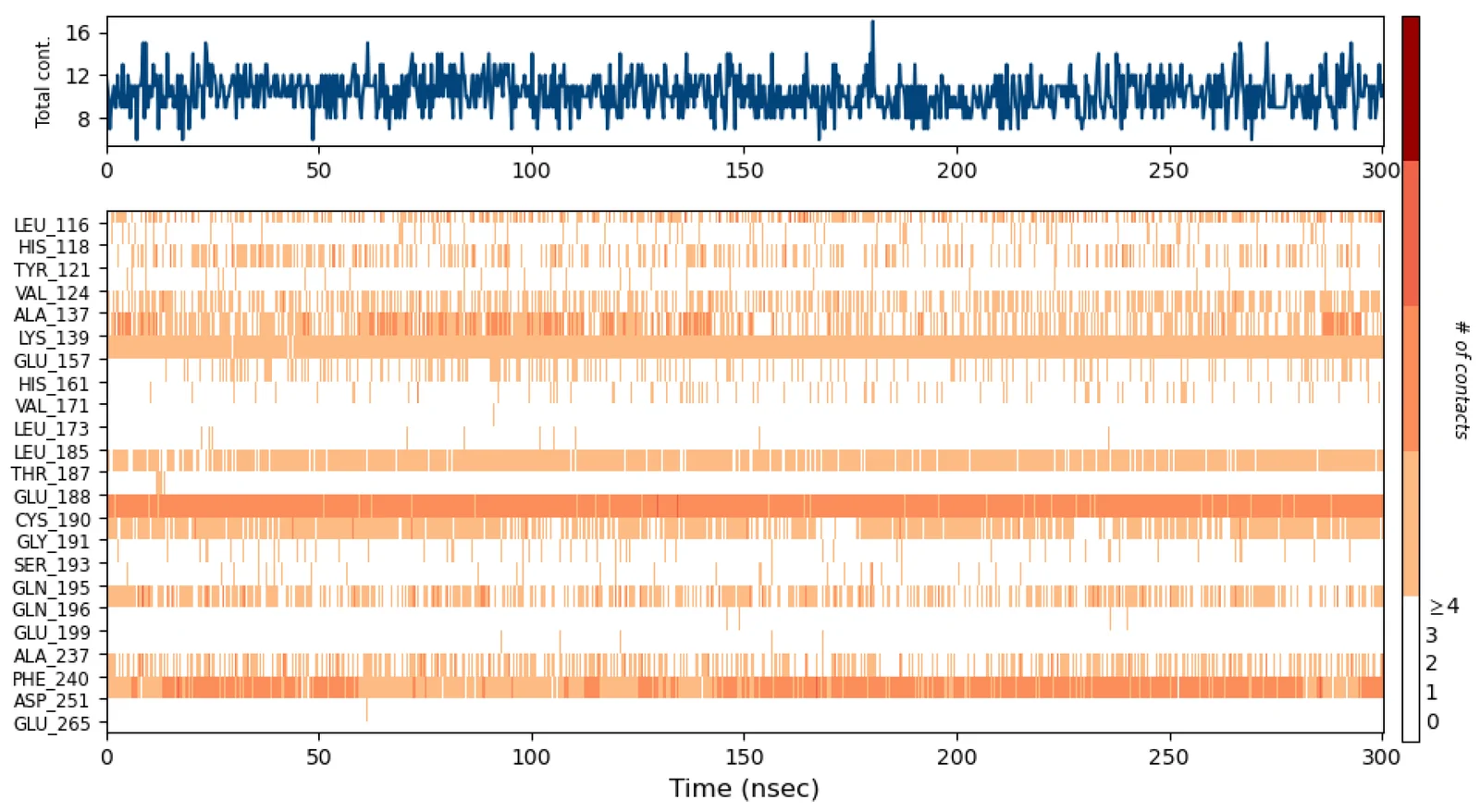
Timeline of interactions between **MS13** and residues from a single representative 300 ns simulation.

As shown in Figure 6B,C, the interaction analysis of the simulated trajectories and the visualization of the interaction timeline in Figure 7 indicate that compound **MS13** forms a stable and persistent hydrogen bond network with key residues ASP251, GLU157, CYS190, and THR187, with hydrogen bond occupancy maintained at over 85% throughout the entire trajectory. As shown in Figure 6B, the hydrogen bond interactions involving ASP251, GLU157, CYS190, and THR187 exhibit consistently low standard deviations with minimal fluctuations across the three independent simulations. This high reproducibility rules out random or transient contacts, underscoring a robust binding mode in which these residues act as critical, stably maintained anchors. Additionally, stable hydrophobic interactions are observed with ALA137, LYS139, and PHE240. The hydrogen bond network mediated by conserved water molecules is indispensable for the binding stability of PKMYT1, with GLY191 and GLN196 forming hydrogen bonds with the nitrogen atom at the 3-position of pyrimidine through water mediation. Furthermore, the hydrogen bonds formed between ASP251, LYS139, and the carbonyl oxygen of the amide bond, mediated by water, enhance the anchoring. The nitro group forms a rich hydrogen bond network with GLN196 and LEU116—these interactions may be key factors in the enhancement of its activity. Notably, the carbonyl oxygen of the amide forms a strong intramolecular hydrogen bond with the amino group at the 4-position of pyrimidine, and **RP-6306** similarly forms a comparable intramolecular hydrogen bond (Figure 6D), demonstrating that this interaction is crucial for stabilizing the molecule’s preferred conformation. Electrostatic surface analysis and cross-sectional analysis, as shown in Figure S1, indicate that compound **MS13** highly matches the binding pocket of PKMYT1.

### 3.10. Computational Alanine Scanning Mutagenesis

**Figure 8:**
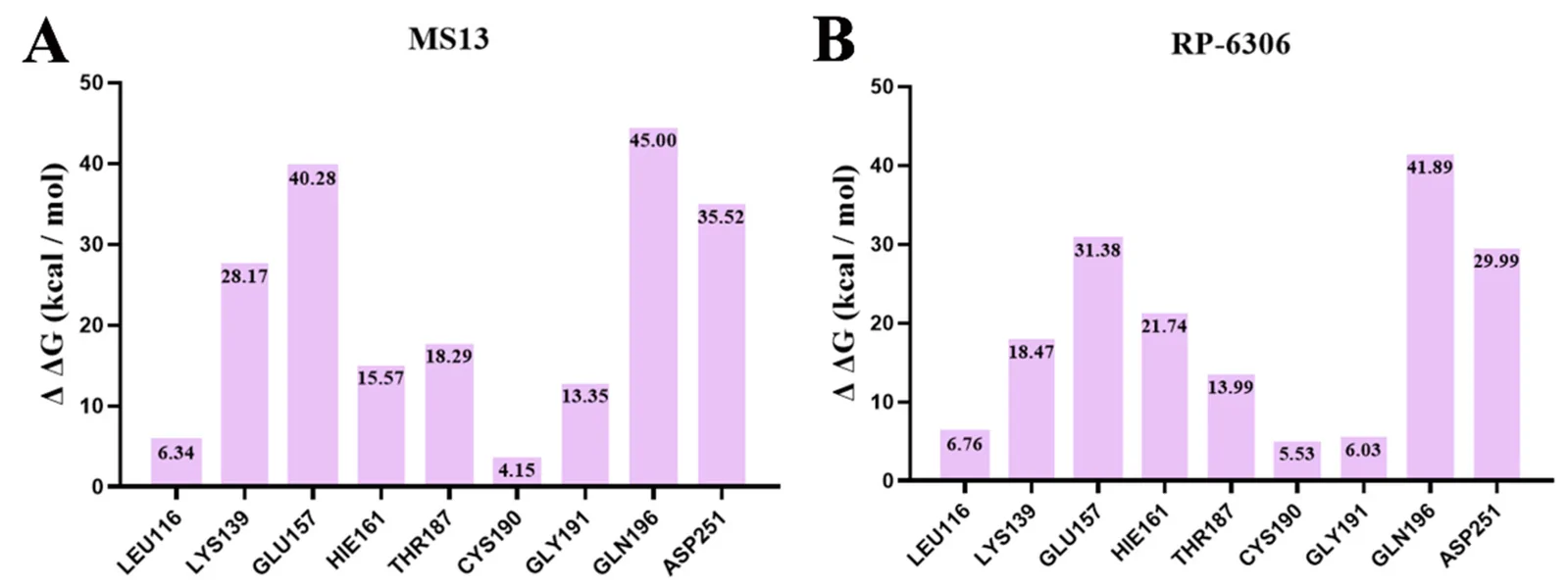
Alanine scanning mutagenesis analysis of the PKMYT1 (PDB ID: 8D6E) complex from the last frame of a single representative MD simulation. The ligands of the complex are (**A**) **MS13** and (**B**) **RP-6306**.

To identify key amino acid residues and validate the binding sites of the complex, the last frame from a single representative 300 ns molecular dynamics simulation was selected to build the reference model. Alanine scanning was performed on nine key amino acids with prolonged action duration to evaluate the energy differences before and after mutation. A positive value indicates that the residue contributes significantly to the binding of the complex, while a negative value indicates the opposite, as shown in Figure 8. All nine amino acids exhibited positive values, suggesting possible residue contributions. **MS13** showed higher positive values on most of the measured residues compared to **RP-6306**, suggesting that **MS13** may possess higher binding specificity. In the DFG region, GLU157 demonstrated a higher contribution value than ASP251, while HIS161 also exhibited a notable contribution. It is noteworthy that the key residue GLN196 has a significant impact on ligand binding, which may explain why **MS13** targets GLN196, exhibiting kinase binding affinity comparable to that of **RP-6306**.

### 3.11. MM-GBSA Binding Energy Analysis

**Figure 9:**
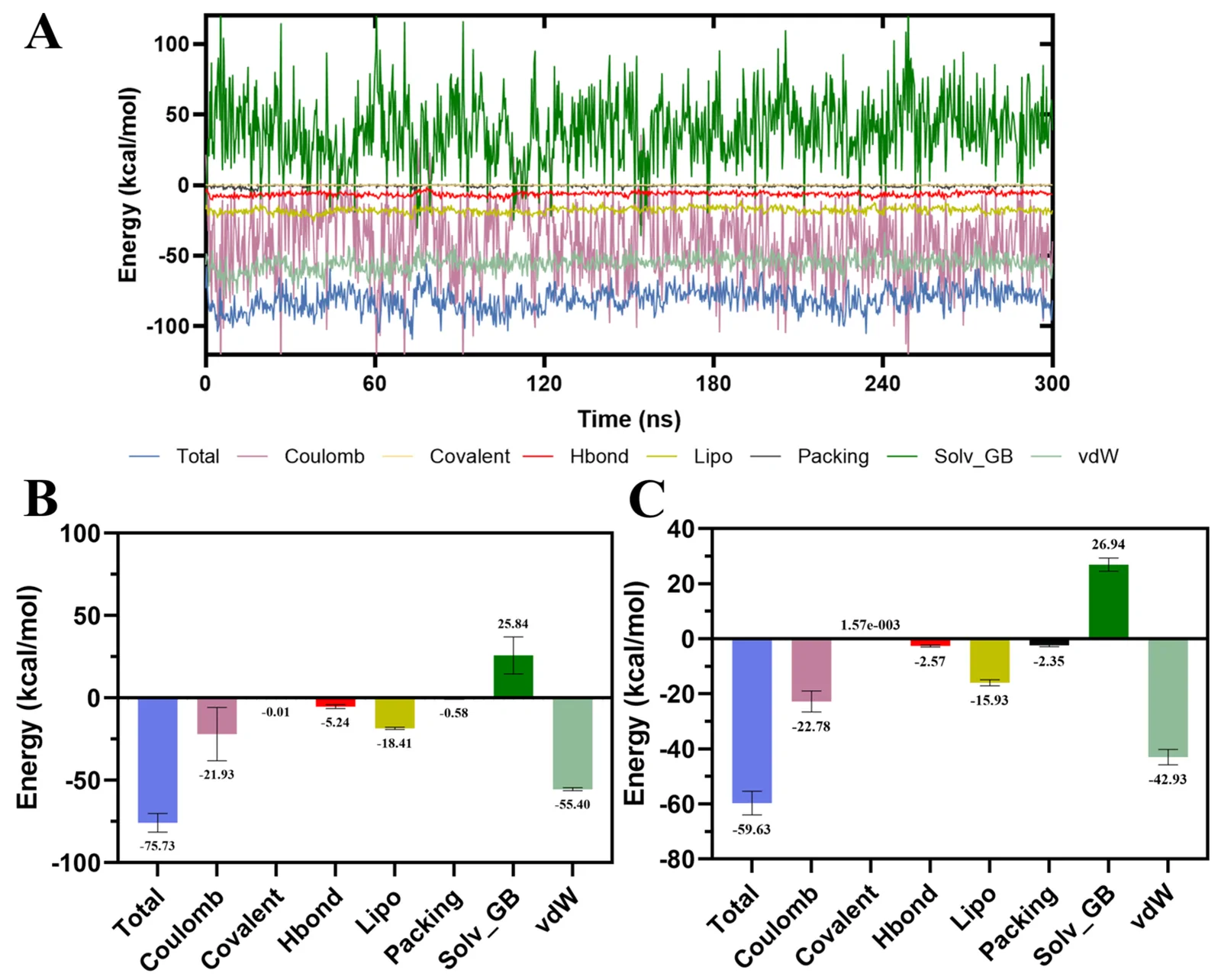
MM-GBSA binding energy analysis. Frames were extracted every 0.3 ns for energy calculation. (**A**) Time evolution of each MM-GBSA energy component of the **MS13**-PKMYT1 complex during a single representative 300 ns MD simulation. (**B**) Mean ± SD of each MM-GBSA energy component for the **MS13**-PKMYT1 complex, calculated from all 3000 snapshots across the three independent 300 ns simulations. The error bar represents the standard deviation across the three replicates, fully accounting for run-to-run variability. (**C**) Mean ± SD of each MM-GBSA energy component for the **RP-6306**-PKMYT1 complex, calculated from all 1000 snapshots across the one independent 300 ns simulation. The error bars represent the standard deviation across all frames within this simulation.

For **MS13**, based on the MD trajectories obtained from at least three independent 300 ns molecular dynamics simulations, snapshots were extracted every 0.3 ns to calculate the MM-GBSA binding energy. Figure 9A,B show the time evolution of the binding energy of the **MS13**-PKMYT1 complex and a bar chart of the average energy components, respectively. For the control group **RP-6306**, the time evolution of its binding energy is shown in Figure S2, and a bar chart of the average energy components is presented in Figure 9C. Given that **RP-6306** is the co-crystallized ligand of PKMYT1 (PDB ID: 8D6E) and its binding mode has been experimentally resolved, we performed only a single 300 ns molecular dynamics simulation and subsequently calculated the binding energy. The binding free energy of **MS13** is −75.73 kcal/mol, which is more negative than that of **RP-6306** (−59.63 kcal/mol), indicating slightly stronger binding affinity. Energy decomposition analysis indicates that van der Waals interactions, electrostatic interactions, and the Lipo term are all negative and remain consistently favorable throughout the simulation, serving as the primary driving forces for binding. The binding free energy difference between **MS13** and **RP-6306** is mainly attributed to van der Waals interactions, raising the possibility that **MS13** might occupy the hydrophobic space within the binding pocket somewhat more fully than **RP-6306**, which could contribute to improved shape complementarity.

## 4. Discussion

As a master kinase governing the G2/M cell cycle checkpoint, PKMYT1 constitutes a therapeutically exploitable vulnerability across multiple malignancies, particularly in CCNE1-amplified tumors, in which its pharmacological blockade triggers mitotic catastrophe. The clinical landscape of PKMYT1 inhibition has evolved rapidly over the past five years. To date, five compounds have entered clinical trials, and a growing number of pharmaceutical companies are pursuing programs in pursuit of this target, as reflected by a surge in patent filings for both PKMYT1 inhibitors and degraders since 2021. Of the four compounds currently in Phase I clinical trials other than **RP-6306**, three (**XL-495**, **QLS-1209**, and **ACR-2316**) share similar structural frameworks or functional groups with **RP-6306**. However, **XL-495** has been discontinued from Phase I trials due to an unfavorable risk–benefit profile. PKMYT1 inhibitors exemplified by **RP-6306** predominantly act as Type I ATP-competitive binders. Recent research has yielded breakthroughs in PKMYT1 inhibitor development, highlighted by the discovery of the allosteric modulator **P29** [33]. **P29** drives a distinctive conformational rearrangement of the PKMYT1 P-loop, unambiguously validating a previously unrecognized allosteric regulatory pocket; this structural insight lays a solid foundation for the rational design of next-generation, highly selective allosteric PKMYT1 inhibitors and corresponding PROTAC degraders. The structural evolution from compound **6** to compound **24** also provides a feasible scaffold-hopping strategy to overcome the therapeutic bottleneck of single-agent PKMYT1 inhibition by simultaneously targeting both WEE1 and PKMYT1 [34].

However, several factors merit careful consideration. First, among the currently reported PKMYT1 inhibitors, **RP-6306** derivatives are predominant, with most programs still relying on this common scaffold rather than exploring new chemical architectures, raising concerns about insufficient chemical diversity. Second, the clinical setback of **XL-495** (a structurally analogous inhibitor that entered Phase I trials but was discontinued due to an unfavorable risk–benefit profile) highlights the challenges of translating potent enzymatic inhibition into a clinically acceptable therapeutic index with a favorable safety profile. Collectively, these considerations underscore the urgent need for structurally novel PKMYT1 inhibitors with improved safety profiles.

The present study reports a chemotype with strategically modified substituents and distinct binding modes. This series exhibits potent inhibitory activity, and conformational restriction is adopted to enhance the molecular stability of target compounds, representing a classic rational drug design strategy. Our findings provide a direction for the subsequent modification of the compound.

This study has limitations. **MS13** is currently a preliminary tool compound; while it displays moderate cellular selectivity and improved stability, it has not yet been evaluated in vivo, its broader kinase selectivity remains to be determined, and direct cellular mechanistic verification is lacking. Compared with clinical-stage candidates such as **RP-6306**, **MS13** still exhibits a significant gap in potency and overall profile, and thus is best considered primarily as a tool compound for PKMYT1. Nevertheless, it is worth noting that in our previous work, compound **A30**, which shares a similar core scaffold to the compounds in the present study, exhibited excellent selectivity for PKMYT1. Given the similar scaffold between our current molecules and **A30**, we hold a cautiously optimistic expectation regarding their kinase selectivity. In future work, after further optimization of this series to achieve desirable potency and physicochemical properties, we will dedicate resources to systematic selectivity profiling of the most promising compounds, so as to rigorously validate their target specificity.

In this PKMYT1 inhibitor series, the 2,6-dimethylphenol moiety confers axial chirality to most analogs. Notably, pronounced atropisomerism was observed in the ^1^H NMR spectra of **MS19**-**MS24**, giving rise to two distinct sets of proton signals with an approximate 6:4 ratio. Although this atropisomerism generally does not compromise enzyme inhibitory activity, it can lead to a marked decrease in cellular potency. Compound **MS13** is currently only a preliminary tool molecule at this stage. Although no obvious atropisomerism was observed in its ^1^H NMR spectra, it may still possess axial chirality. However, the presence of the chiral axis does not affect the direction of subsequent structural modification and optimization.

**MS13**, as the most promising compound in this series, bears an aromatic nitro moiety in its chemical structure, which warrants particular attention from a medicinal chemistry safety perspective. Although nitro-containing drugs have a long history of clinical use (e.g., metronidazole), aromatic nitro groups have long been recognized as typical structural alerts. Upon metabolic activation by CYP450 enzymes and nitroreductases, they undergo sequential reduction to generate reactive intermediates such as nitroso and hydroxylamine species. These electrophilic intermediates are capable of covalently modifying DNA and proteins, thereby leading to mutagenesis and cytotoxicity. To circumvent the inherent toxicological liabilities associated with the nitro group, our team has initiated systematic medicinal chemistry efforts to explore bioisosteric replacements, including cyano (-CN) and *ortho*-difluoro disubstitution. This strategy aims to preserve molecular polarity and hydrogen-bonding capacity to maintain target binding affinity for PKMYT1, while effectively eliminating the reductive metabolic pathway that generates toxic intermediates, ultimately enabling the identification of candidate molecules with an improved safety window.

## 5. Conclusions

In summary, to overcome the limitations of the chemical framework primarily based on **RP-6306**, we systematically investigated structural modifications and structure–activity relationships, designing and synthesizing a series of new 2-aminopyrimidine compounds. Among them, **MS13** exhibited the best overall performance, with kinase binding affinity comparable to that of **RP-6306**. In *CCNE1*-amplified OVCAR3 and HCC1569 cells, **MS13** also demonstrated strong anti-proliferative effects, while showing lower toxicity in A549 and HEK293T cells with low *CCNE1* expression, indicating a certain selective safety window. Further experiments suggested that **MS13** concentration-dependently inhibited cell colony formation and induced S-phase cell cycle arrest. Metabolic evaluation revealed that **MS13** exhibits moderate stability in human liver microsomes, with an extended half-life compared to the analog **A30**, providing room for further pharmacokinetic optimization. Molecular dynamics simulations showed that **MS13** formed a stable hydrogen bond network and hydrophobic interactions with the PKMYT1 protein, and key residues were computationally suggested through alanine scanning, particularly the multiple hydrogen bonds between the nitro group and residues GLN196, LEU116, and TYR121, as well as the intramolecular hydrogen bonds contributing to conformational stability, which may be the structural basis for its high activity. **MS13** is a promising, highly potent tool compound that provides a clear direction for the future optimization of PKMYT1 inhibitors and warrants further investigation.

## Figures and Tables

**Table 1:** In vitro kinase binding affinity of compounds **MS1**–**MS16**.

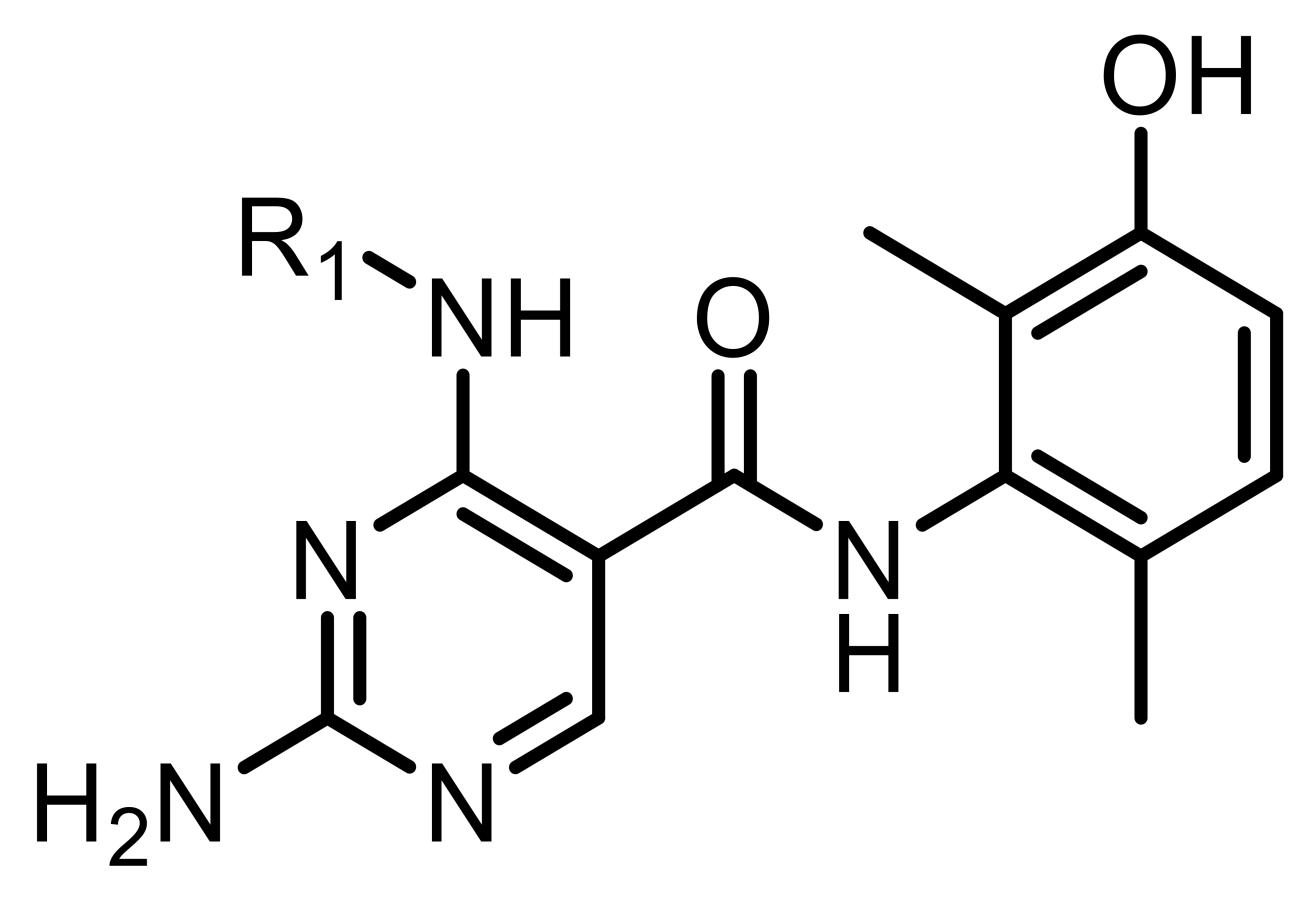					
Compd.	R_1_	PKMYT1 IC_50_ (nM) *^a^*	Compd.	R_1_	PKMYT1 IC_50_ (nM) *^a^*
**MS1**	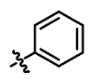	4.56 ± 0.06	**MS10**	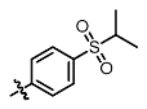	6.62 ± 0.19
**MS2**	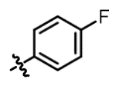	3.01 ± 0.07	**MS11**	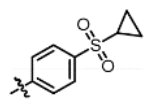	2.51 ± 0.09
**MS3**	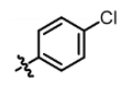	5.96 ± 0.04	**MS12**	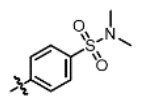	3.59 ± 0.22
**MS4**	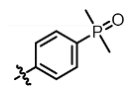	10.07 ± 0.62	**MS13**	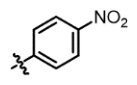	0.86 ± 0.44
**MS5**	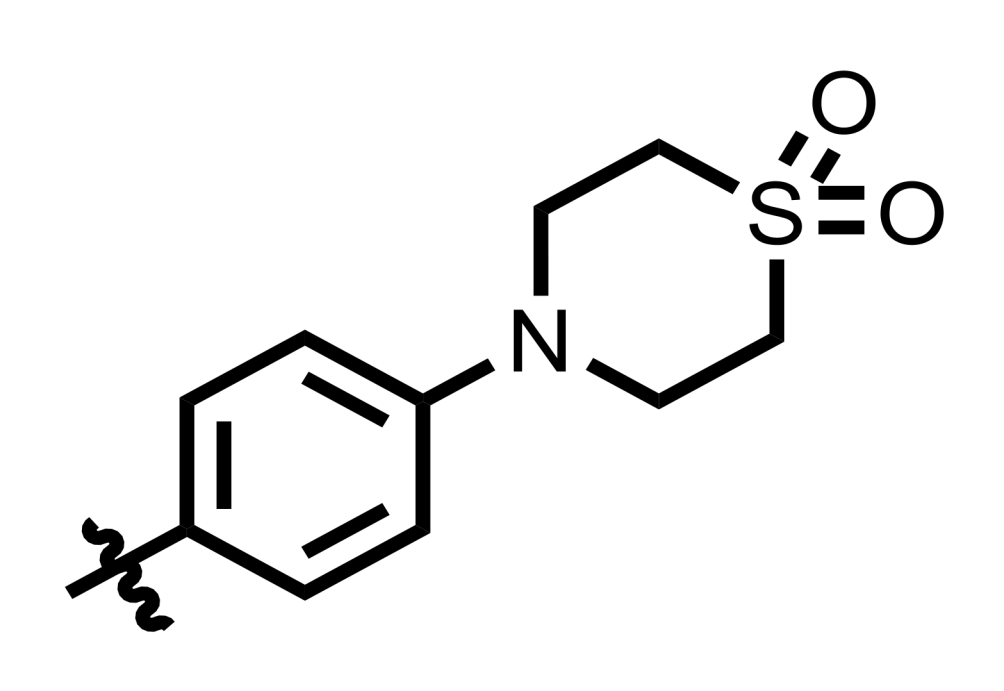	10.66 ± 0.23	**MS14**	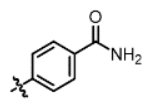	2.57 ± 0.12
**MS6**	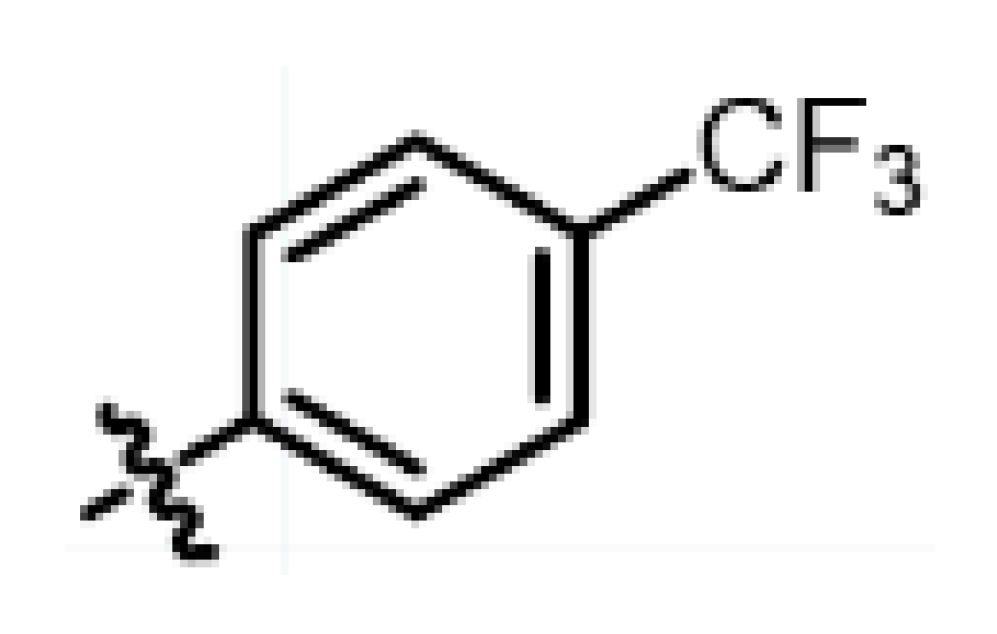	13.26 ± 1.80	**MS15**	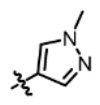	6.14 ± 0.08
**MS7**	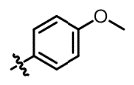	6.31 ± 0.09	**MS16**	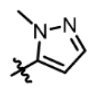	8.74 ± 0.39
**MS8**	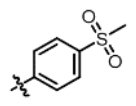	7.32 ± 0.31	**RP-6306**	-	0.74 ± 0.04
**MS9**	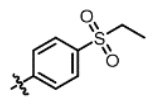	6.71 ± 0.24			

*^a^* IC_50_ values are shown as the mean (nM) ± SD of three separate experiments.

**Table 2:** In vitro kinase binding affinity of compounds **MS17**–**MS18**.

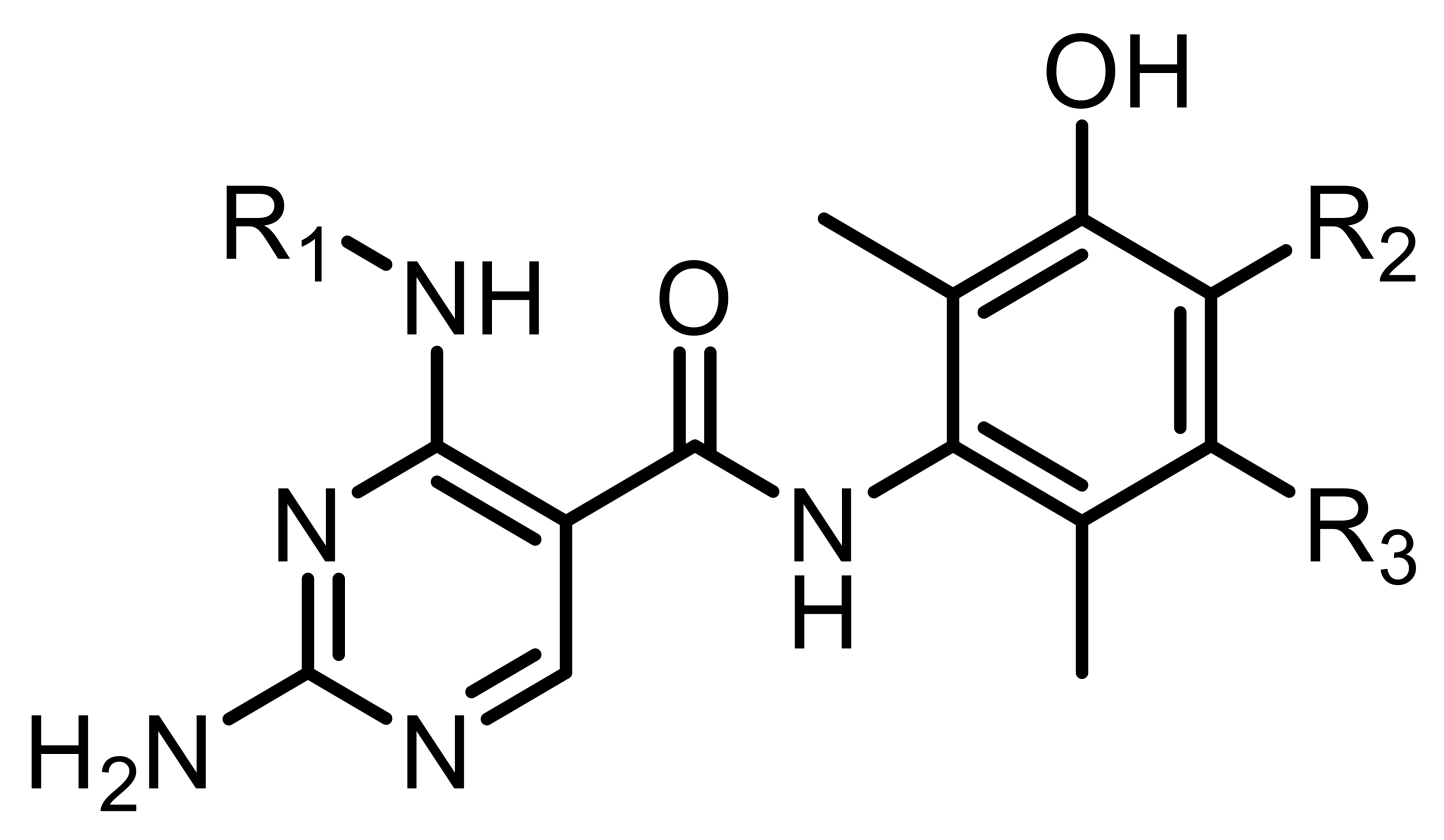							
Compd.	R_1_	R_2_	PKMYT1 IC_50_ (nM) *^a^*	Compd.	R_1_	R_3_	PKMYT1 IC_50_ (nM) *^a^*
**MS17**	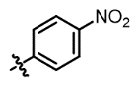	F	27.91 ± 1.05	**MS18**	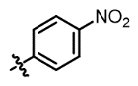	F	7.15 ± 1.01

*^a^* IC_50_ values are shown as the mean (nM) ± SD of three separate experiments.

**Table 3:** In vitro kinase binding affinity of compounds **MS19**–**MS24**.

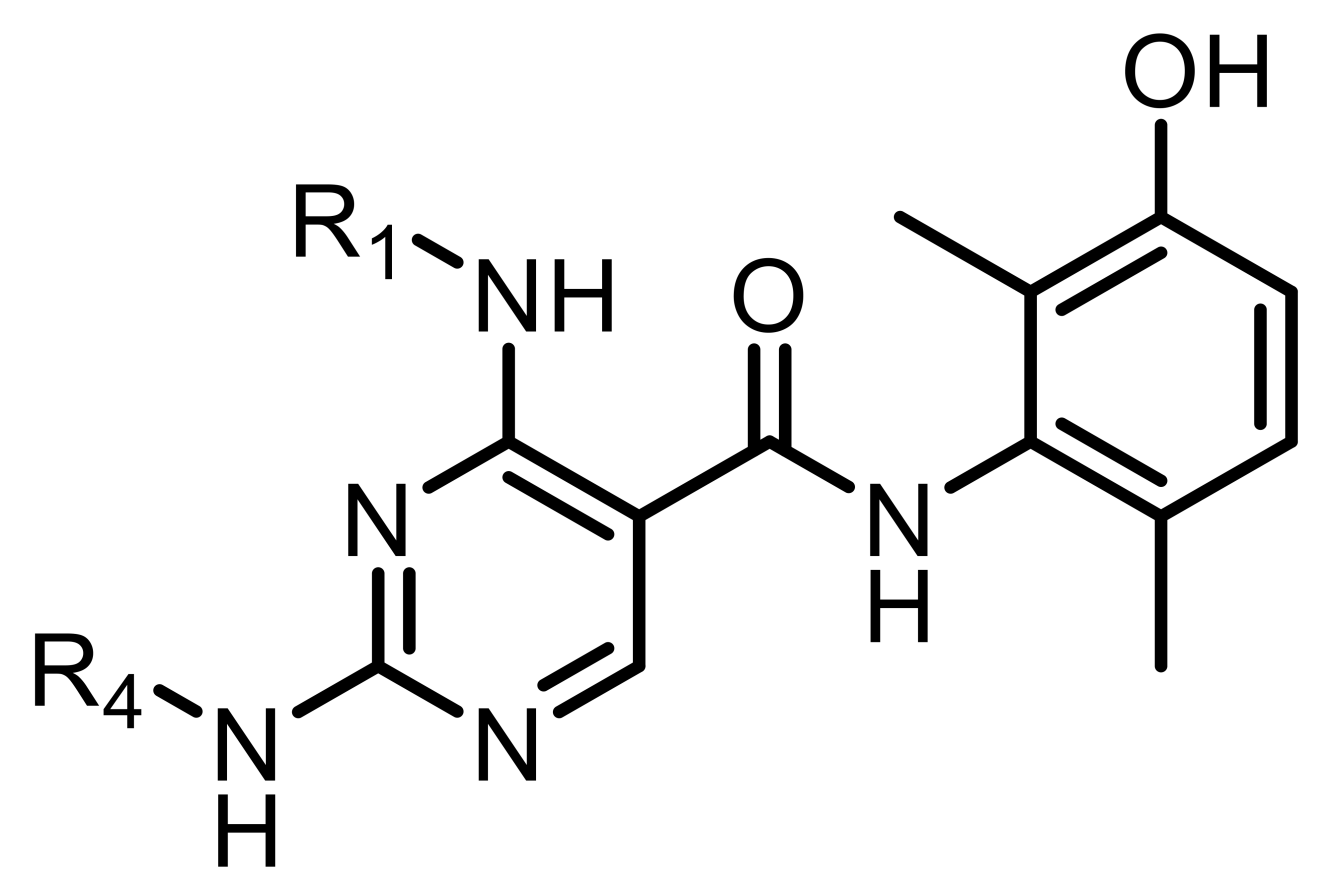							
Compd.	R_1_	R_4_	PKMYT1 IC_50_ (nM) *^a^*	Compd.	R_1_	R_4_	PKMYT1 IC_50_ (nM) *^a^*
**MS19**	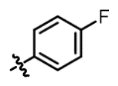	-CH_3_	4.24 ± 0.23	**MS23**	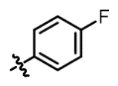	-CH_2_CH_3_	34.03 ± 1.26
**MS20**	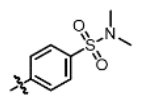	-CH_3_	7.87 ± 0.55	**MS24**	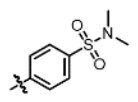	-CH_2_CH_3_	9.20 ± 0.25
**MS21**	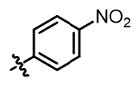	-CH_3_	2.83 ± 0.08	**RP-6306**	-	-	0.74 ± 0.04
**MS22**	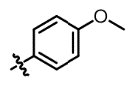	-CH_3_	5.64 ± 0.16				

*^a^* IC_50_ values are shown as the mean (nM) ± SD of three separate experiments.

**Table 4:** In vitro kinase binding affinity and antiproliferative activities of selected compounds against OVCAR3 cells.

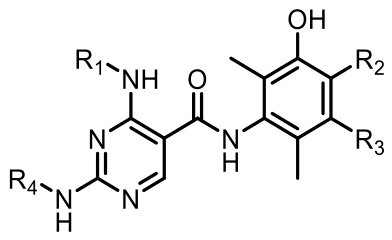						
Compd.	R_1_	R_2_	R_3_	R_4_	PKMYT1 IC_50_ (nM) *^a^*	OVCAR3 IC_50_ (μM) *^b^*
**MS1**	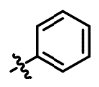	-	-	-	4.56 ± 0.06	1.85 ± 0.31
**MS2**	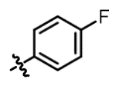	-	-	-	3.01 ± 0.07	1.45 ± 0.35
**MS3**	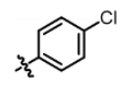	-	-	-	5.96 ± 0.04	4.48 ± 0.99
**MS4**	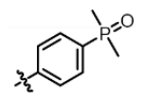	-	-	-	10.07 ± 0.62	>8
**MS7**	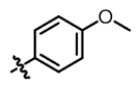	-	-	-	6.31 ± 0.09	1.91 ± 0.43
**MS8**	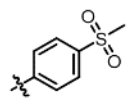	-	-	-	7.32 ± 0.31	1.53 ± 0.14
**MS9**	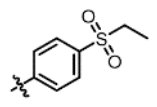	-	-	-	6.71 ± 0.24	1.93 ± 0.63
**MS10**	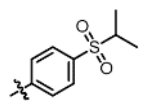	-	-	-	6.62 ± 0.19	1.80 ± 0.31
**MS11**	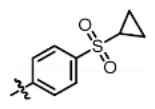	-	-	-	2.51 ± 0.09	2.00 ± 0.77
**MS12**	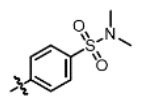	-	-	-	3.59 ± 0.22	1.67 ± 0.22
**MS13**	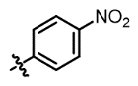	-	-	-	0.86 ± 0.44	1.52 ± 0.38
**MS14**	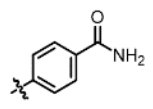	-	-	-	2.57 ± 0.12	6.15 ± 1.40
**MS15**	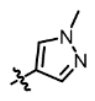	-	-	-	6.14 ± 0.08	3.42 ± 0.53
**MS16**	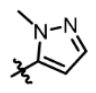	-	-	-	8.74 ± 0.39	1.50 ± 0.34
**MS17**	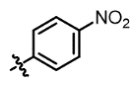	F	-	-	27.91 ± 1.05	3.40 ± 1.41
**MS18**	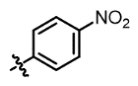	-	F	-	7.15 ± 1.01	1.52 ± 0.19
**MS19**	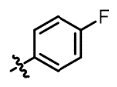	-	-	-CH_3_	4.24 ± 0.23	4.76 ± 0.38
**MS20**	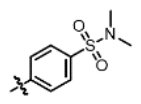	-	-	-CH_3_	7.87 ± 0.55	2.03 ± 0.65
**MS21**	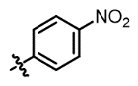	-	-	-CH_3_	2.83 ± 0.08	6.21 ± 0.67
**MS22**	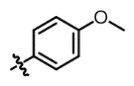	-	-	-CH_3_	5.64 ± 0.16	2.54 ± 0.40
**RP-6306**	-	-	-	-	0.74 ± 0.04	0.20 ± 0.04

*^a^* IC_50_ values are shown as the mean (nM) ± SD of three separate experiments. *^b^* *CCNE1*-amplified cell lines. Data are expressed as the mean (μM) ± SD of three separate experiments.

**Table 5:** In vitro kinase binding affinity and antiproliferative activities of selected compounds against HCC1569 cells.

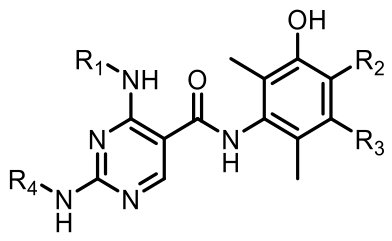						
Compd.	R_1_	R_2_	R_3_	R_4_	PKMYT1 IC_50_ (nM) *^a^*	HCC1569 IC_50_ (μM) *^b^*
**MS2**	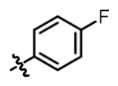	-	-	-	3.01 ± 0.07	3.97 ± 0.99
**MS7**	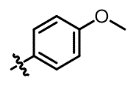	-	-	-	6.31 ± 0.09	2.77 ± 0.61
**MS8**	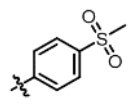	-	-	-	7.32 ± 0.31	5.62 ± 0.58
**MS12**	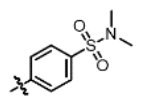	-	-	-	3.59 ± 0.22	4.13 ± 0.91
**MS13**	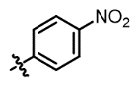	-	-	-	0.86 ± 0.44	0.66 ± 0.14
**MS16**	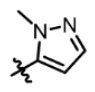	-	-	-	8.74 ± 0.39	3.66 ± 0.21
**MS17**	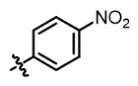	F	-	-	27.91 ± 1.05	1.19 ± 0.26
**MS18**	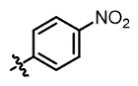	-	F	-	7.15 ± 1.01	1.36 ± 0.22
**MS20**	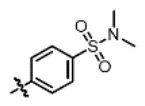	-	-	-CH_3_	7.87 ± 0.55	1.09 ± 0.24
**MS22**	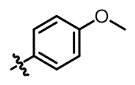	-	-	-CH_3_	5.64 ± 0.16	0.75 ± 0.08
**RP-6306**	-	-	-	-	0.74 ± 0.04	0.10 ± 0.01

*^a^* IC_50_ values are shown as the mean (nM) ± SD of three separate experiments. *^b^* *CCNE1*-amplified cell lines. Data are expressed as the mean (μM) ± SD of three separate experiments.

**Table 6:** The antiproliferative activity of **MS13** in *CCNE1*-amplified cells and low-*CCNE1*-expressing cells.

Compd.	OVCAR3 IC_50_ (μM) ^*a*^	HCC1569 IC_50_ (μM) ^*a*^	A549 IC_50_ (μM) ^*b*^	HEK293T IC_50_ (μM) ^*b*^	A549/ OVCAR3 *^c^*	HEK293T/ OVCAR3 *^c^*	A549/ HCC1569 *^c^*	HEK293T/ HCC1569 *^c^*
**MS13**	1.52 ± 0.38	0.66 ± 0.14	6.55 ± 0.05	3.10 ± 0.09	4.31	2.04	9.92	4.70
**RP-6306**	0.20 ± 0.04	0.10 ± 0.01	1.63 ± 0.05	0.63 ± 0.06	8.15	3.15	16.30	6.30

*^a^* *CCNE1*-amplified cell lines. Data are expressed as the mean (μM) ± SD of three separate experiments. *^b^* Low-*CCNE1*-expressing cell line. Data are expressed as the mean (μM) ± SD of three separate experiments. *^c^* Selectivity indices.

**Table 7:** Liver microsomal stability of compound **MS13**.

	Species	Human			
Compound		Final Remaining (%)	R^2^	T_1/2_ (min)	In Vitro CL_int_ (mL/min/mg)
**Midazolam**		11.1	0.9985	4.76	0.728
**MS13**		28.7	0.9821	32.2	0.0430

Final Remaining: the percentage of remaining compounds at the end of the incubation period; R^2^: linear correlation coefficient; T_1/2_: the half-life of the compound; CL_int_: intrinsic clearance. **Midazolam** and **MS13** were determined at the 15 min and 60 min time points, respectively.

## Data Availability

The datasets presented in this article are not readily available because the data are part of an ongoing study. Requests to access the datasets should be directed to the corresponding author.

## Supplementary Materials

The following supporting information can be downloaded at: https://www.mdpi.com/article/10.3390/biomedicines14081876/s1.
