# Supplementary material for: Design, Synthesis, Biological Activity Evaluation, and Molecular Docking of 2-Aminopyrimidine-Based PKMYT1 Inhibitors
Source: Biomedicines. 2026 Aug 21;14(8):1876. doi: 10.3390/biomedicines14081876 (PMC13509279; doi:10.3390/biomedicines14081876)
Supplement: Supplementary file 1 [file biomedicines-14-01876-s001.zip › biomedicines-4513794-supplementary.pdf]

Design, Synthesis, and Biological Activity Evaluation of 2-Aminopyrimidine-Based PKMYT1 Inhibitors

Shizhe Yuan[a], Chuanxu Su[a], Chenxi Zhang[a], Haoyu Zhang[a], Jinyu Yu[a], Nian Liu[a], Cunzheng Fan[a], Zixuan Gao[a], Zirui Luo[a], Yin Sun[a], Dongmei Zhao[a]*, Maosheng Cheng[a]

[a] *Key Laboratory of Structure-Based Drug Design and Discovery, Ministry of Education, School of Pharmaceutical Engineering, Shenyang Pharmaceutical University, 103 Wenhua Road, Shenhe District, Shenyang 110016, PR China*

*Corresponding author: Prof. Dongmei Zhao, medchemzhao@163.com.

S1. Dose-response curve of representative compounds in the in vitro enzyme binding affinity assay

Compound MS13

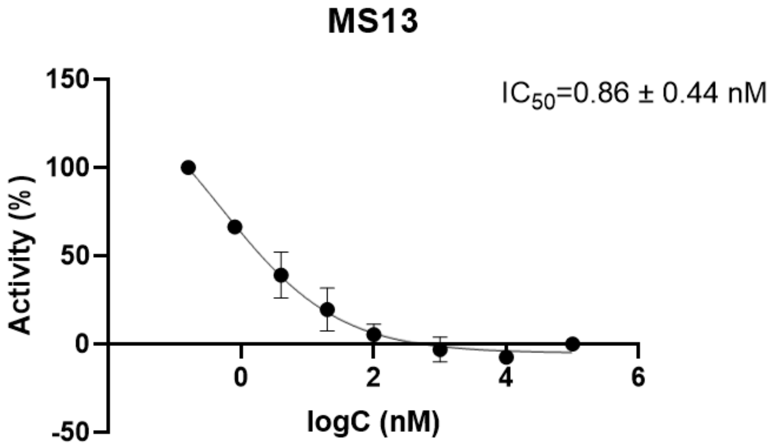

Compound MS2

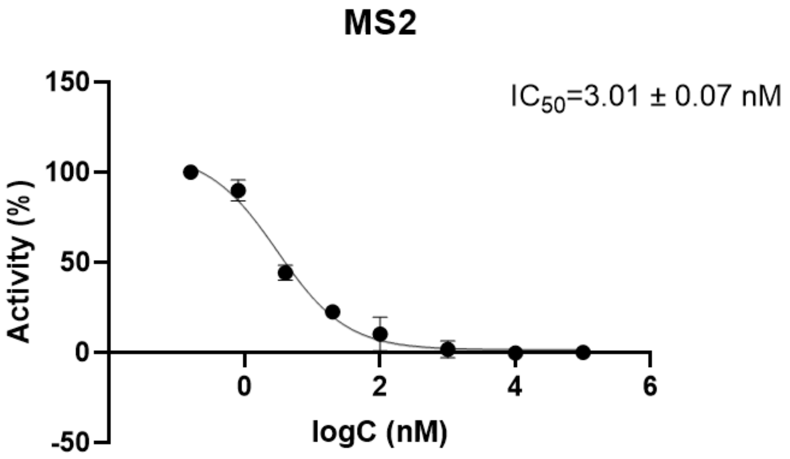

S2. Dose-response curve of representative compounds in the OVCAR3.

Compound MS13

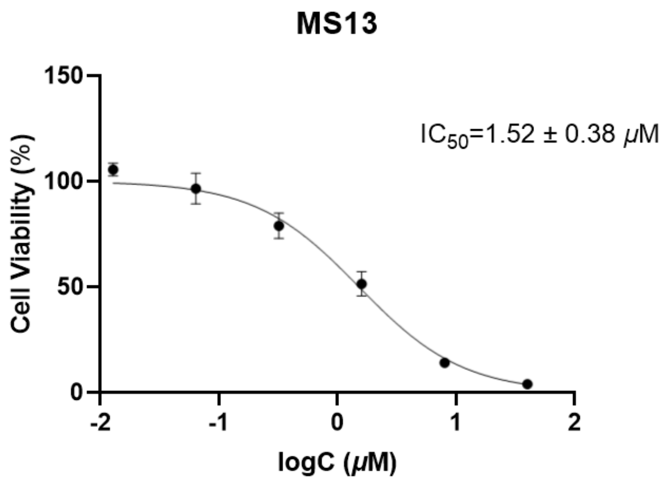

Compound MS2

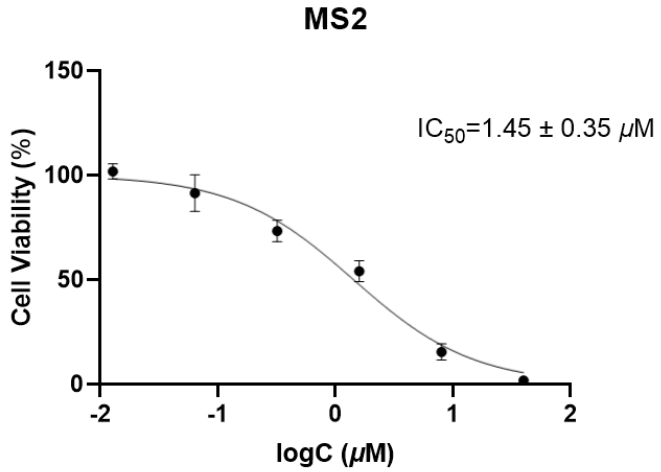

S3. Dose-response curve of representative compounds in the HCC1569.

Compound MS13

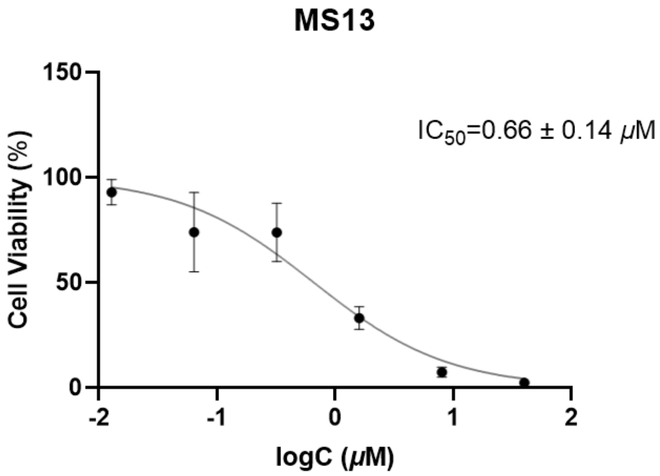

Compound MS2

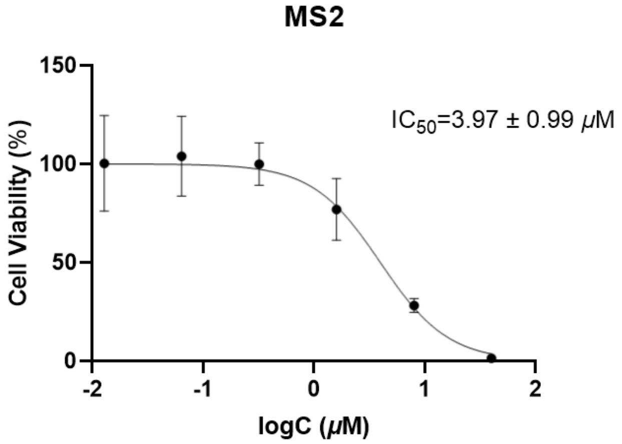

S4. Figure

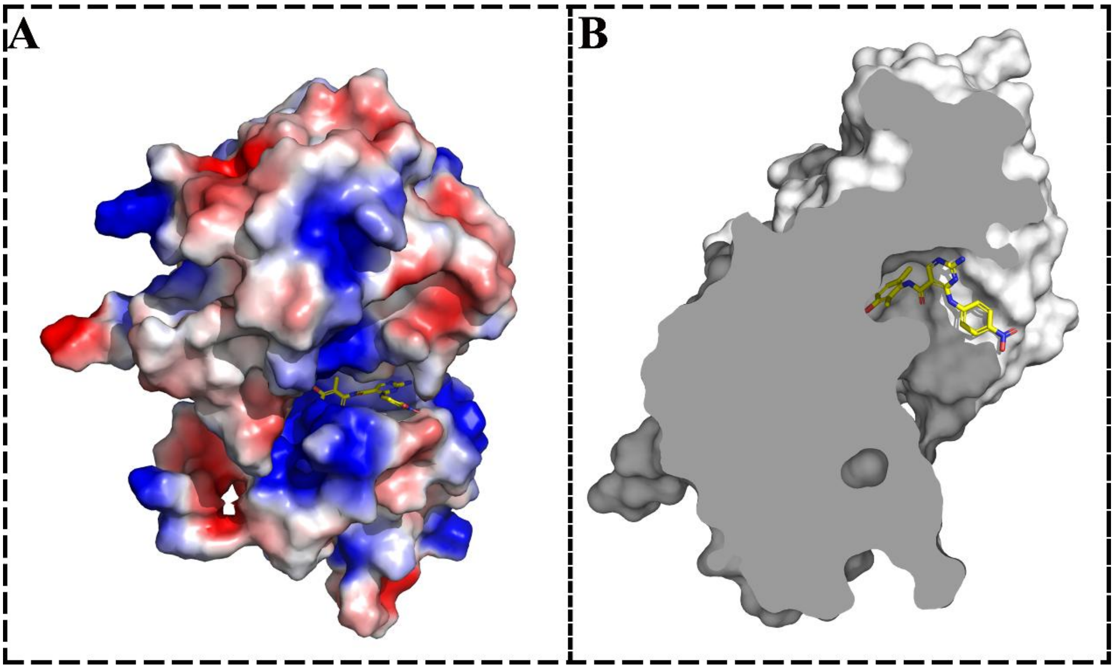

Figure S1. (A) Charge distribution cloud diagram of MS13 (B) Cutaway view of MS13 occupying PKMYT1 catalytic cavity.

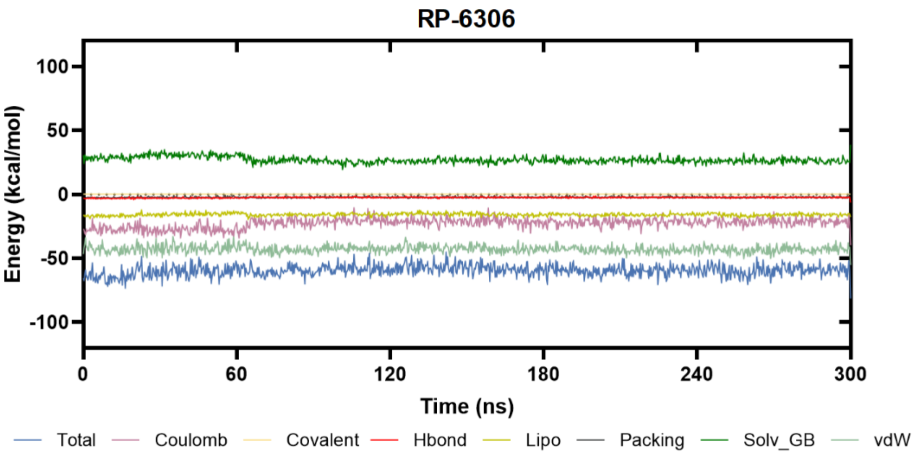

Figure S2. MM-GBSA binding energy analysis. Frames were extracted every 0.3 ns for energy calculation. Time evolution of each MM-GBSA energy component of the RP-6306-PKMYT1 complex during a single representative 300 ns MD simulation.

S5. Chromatogram of the compound

^{1}H NMR and HRMS spectra of Compound MS1a

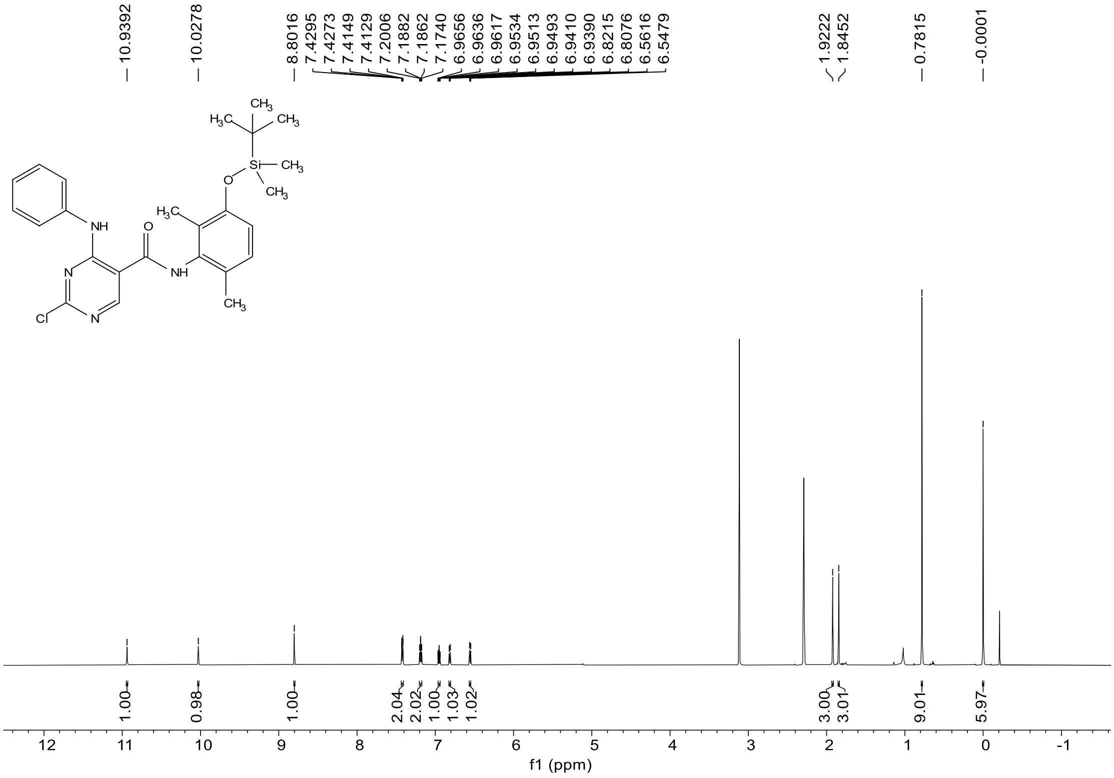

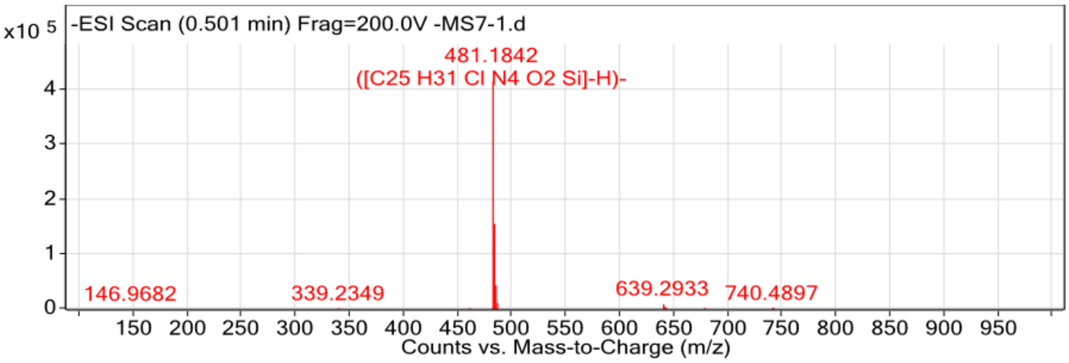

Peak List

m/z	z	Abund	Formula	Ion
481.1842	1	415844.5	C25 H31 Cl N4 O2 Si	(M-H)-
482.1871	1	128909.59	C25 H31 Cl N4 O2 Si	(M-H)-
483.1823	1	156477.69	C25 H31 Cl N4 O2 Si	(M-H)-
484.1839	1	44998.95	C25 H31 Cl N4 O2 Si	(M-H)-
485.1827	1	11619.07	C25 H31 Cl N4 O2 Si	(M-H)-
639.2933	1	10549.4		
640.2964	1	5876.78		
641.2925	1	5500.43		
642.2958	1	2249		
740.4897	1	2397		

^{1}H NMR, ^{13}C NMR, HRMS and HPLC spectra of Compound MS1

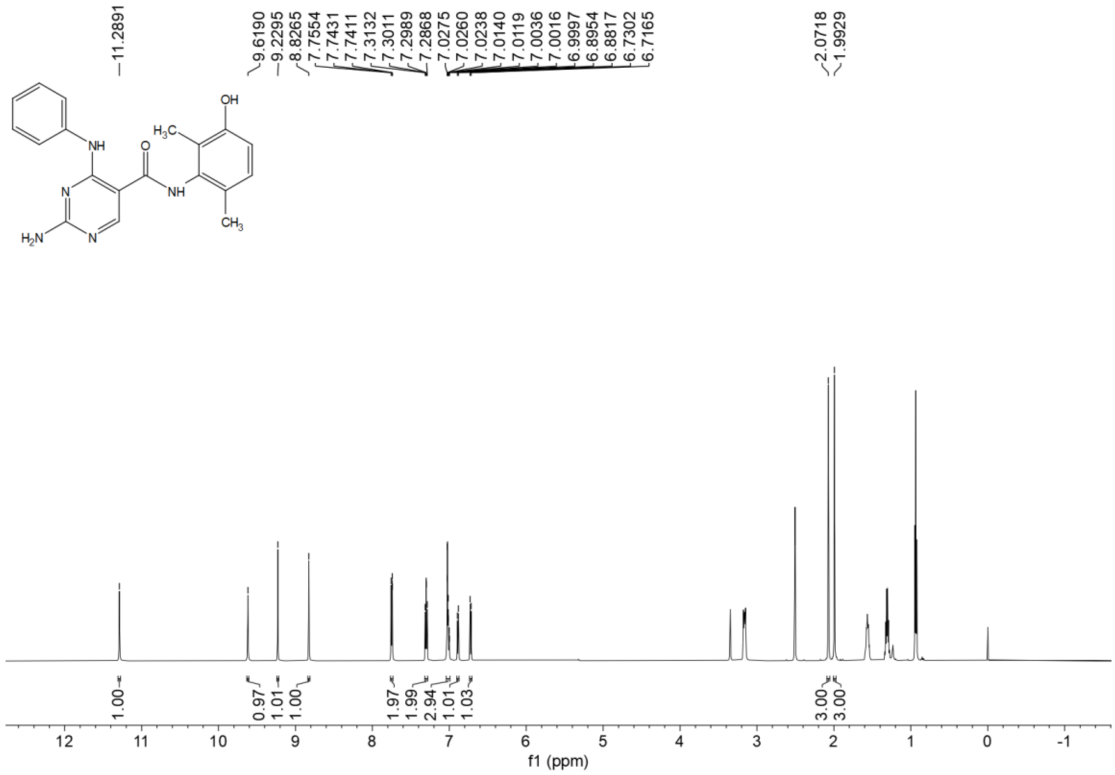

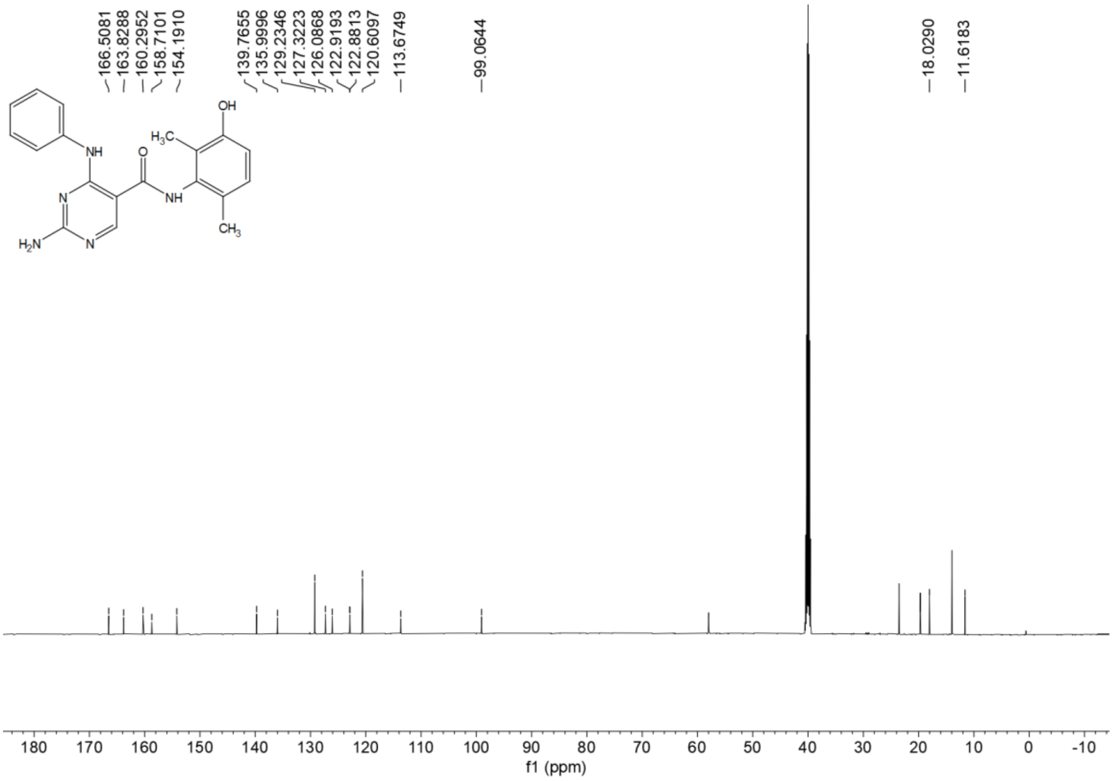

Fragmentor Voltage	Collision Energy	Ionization Mode
200	0	ESI

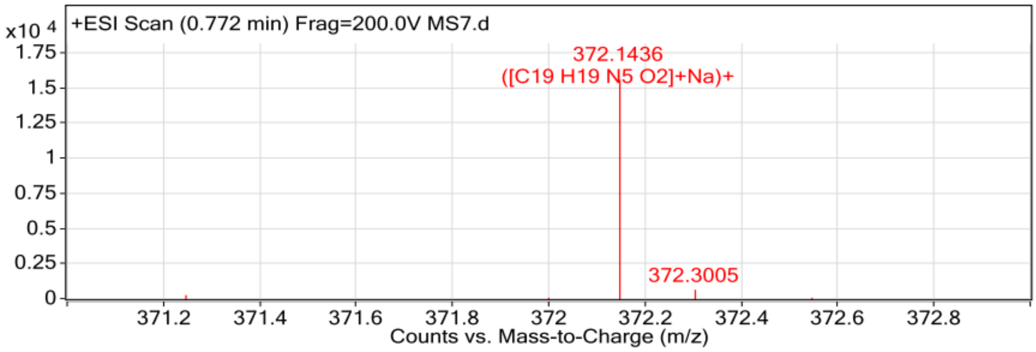

Peak List

m/z	z	Abund	Formula	Ion
239.0232		4774.96		
242.2841	1	21467.69		
301.1419	1	29271.67		
302.1451	1	5221.76		
360.3249	1	6629.01		
372.1436	1	15658.04	C19 H19 N5 O2	(M+Na)+
437.1946	1	5321.84		
472.1193	1	16763.29		
473.1254	1	4180.42		
721.2968	1	5665.73		

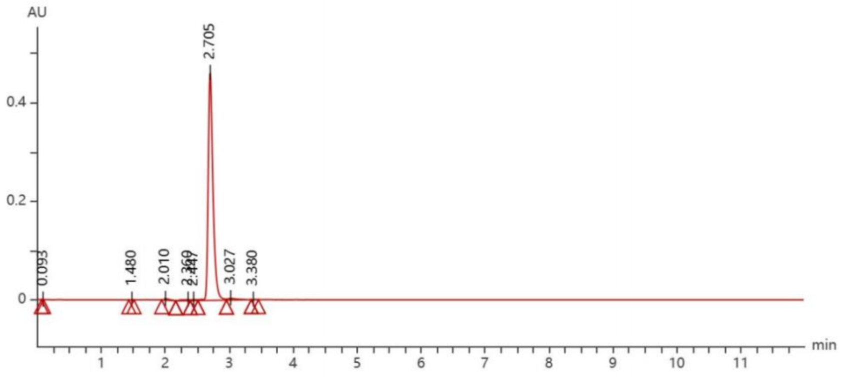

Object List

#	RT (min)	Area (µV*s)	% Area	Height (µV)	% Height
1	0.093	88.512	0.004	147.300	0.031
2	1.480	379.200	0.015	178.500	0.038
3	2.010	19835.899	0.796	3263.046	0.688
4	2.360	22871.671	0.917	2120.746	0.447
5	2.447	13847.536	0.555	2133.624	0.450
6	2.705	2397251.052	96.163	462470.291	97.547
7	3.027	37140.242	1.490	3457.112	0.729
8	3.380	1498.724	0.060	327.036	0.069

^{1}H NMR, ^{13}C NMR, HRMS and HPLC spectra of Compound MS2

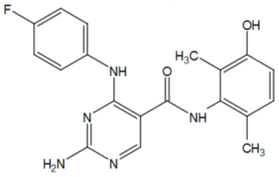

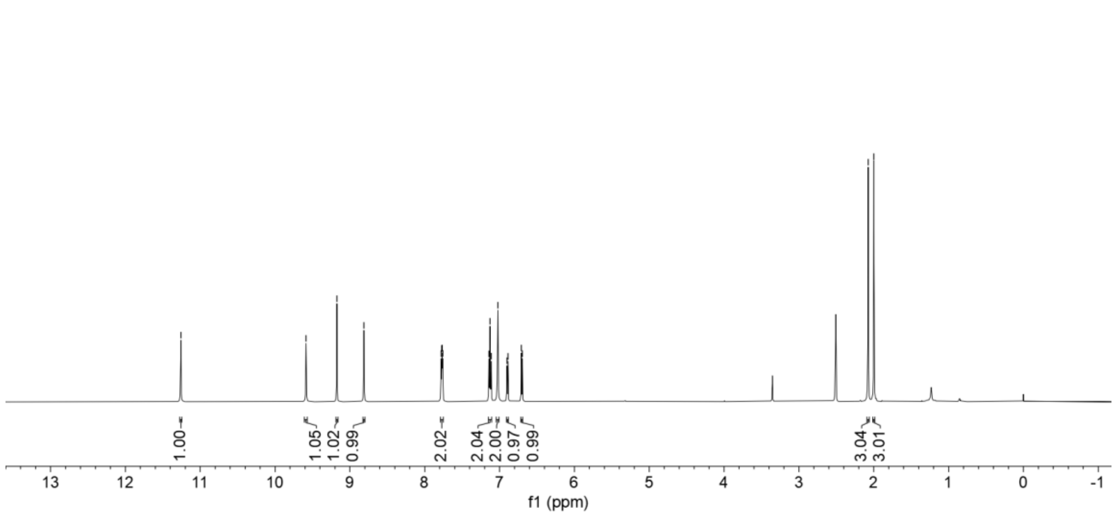

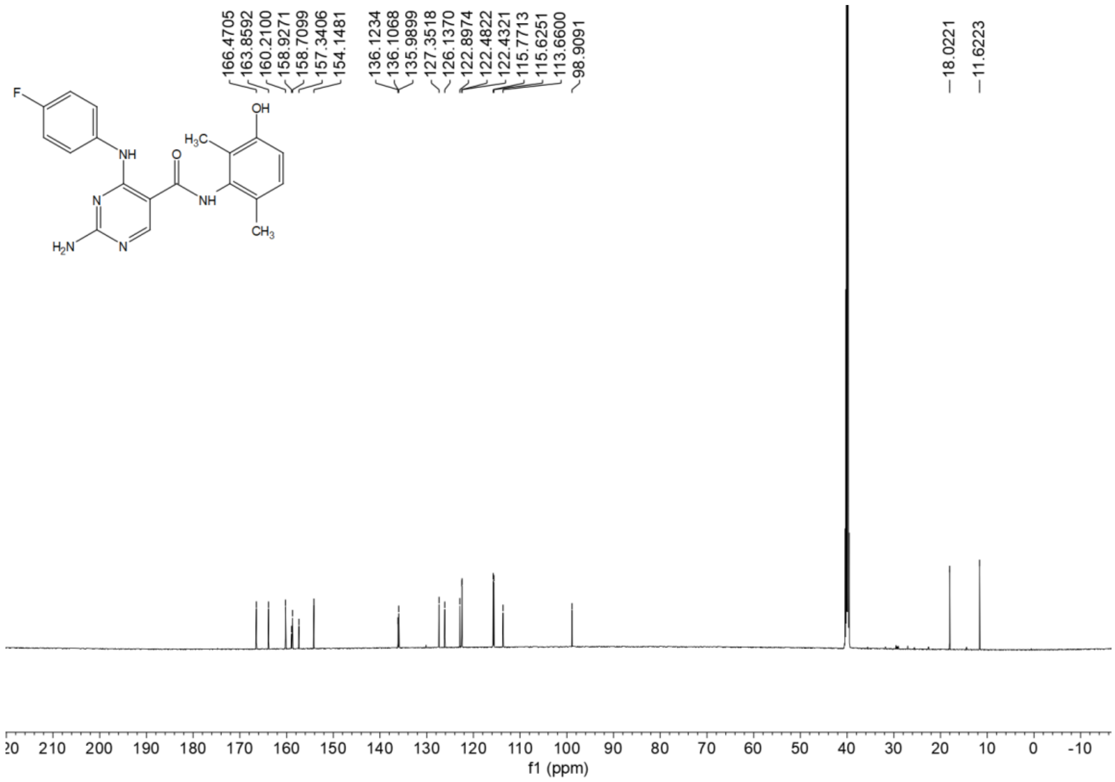

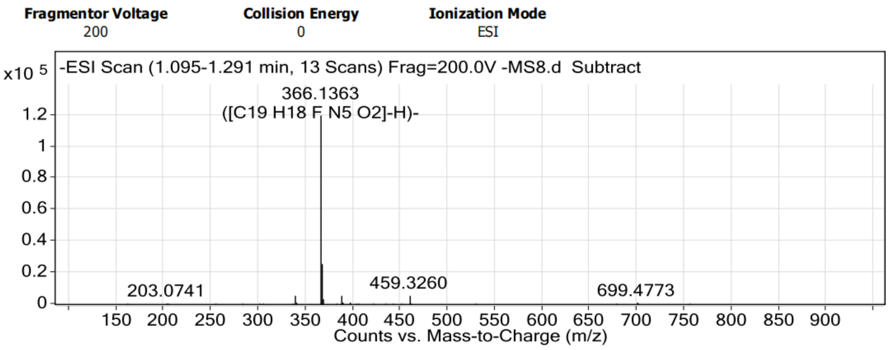

Peak List

m/z	z	Abund	Formula	Ion
339.232	1	5884.43		
340.2351	1	1481.52		
366.1363	1	119940.62	C19 H18 F N5 O2	(M-H)-
367.139	1	25701.23	C19 H18 F N5 O2	(M-H)-
368.1412	1	3530.91	C19 H18 F N5 O2	(M-H)-
388.1179	1	5500.03		
389.1208	1	1245.74		
396.1463	1	1429.2		
459.326	1	5815.12		
460.3292	1	2116.26		

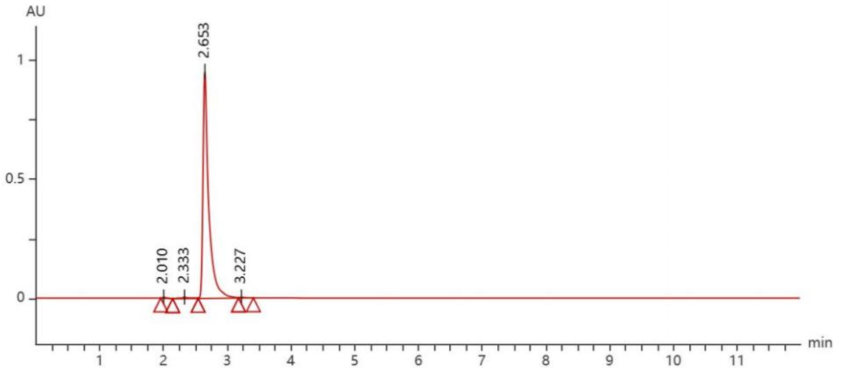

Object List

#	RT (min)	Area (µV*s)	% Area	Height (µV)	% Height
1	2.010	20877.367	0.343	4104.654	0.425
2	2.333	64616.477	1.061	5610.263	0.581
3	2.653	5984621.308	98.284	953948.900	98.714
4	3.227	19016.981	0.312	2712.497	0.281

^{1}H NMR, ^{13}C NMR, HRMS and HPLC spectra of Compound MS3

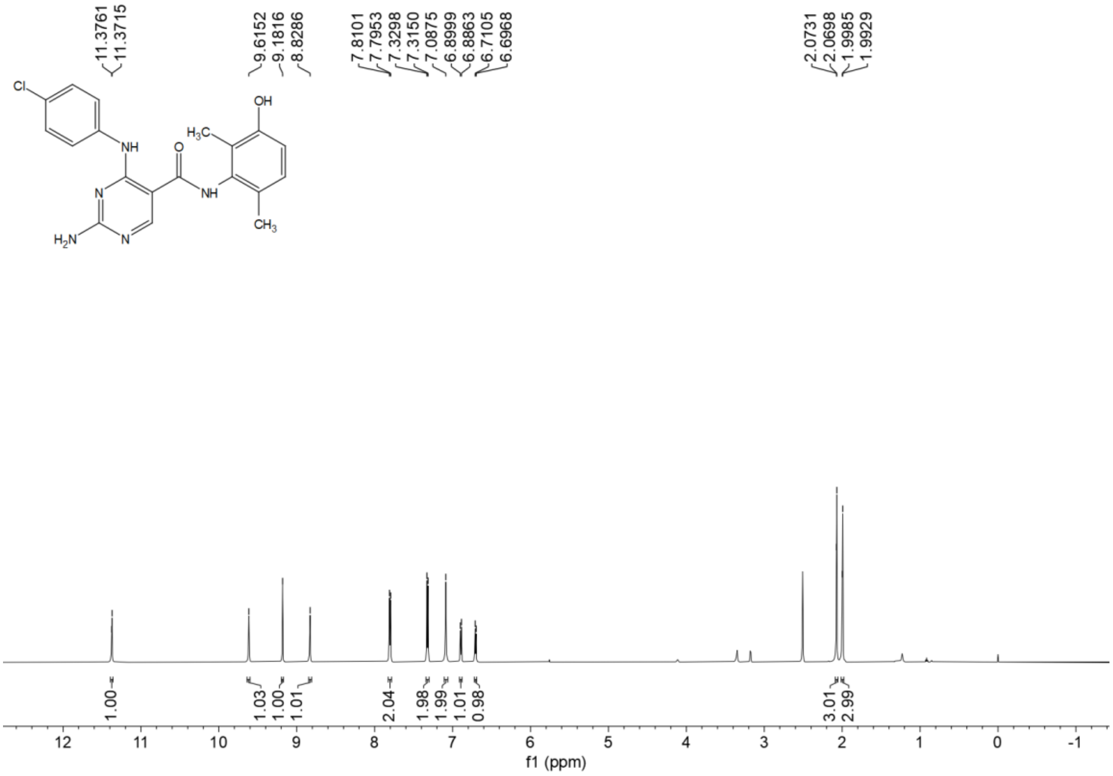

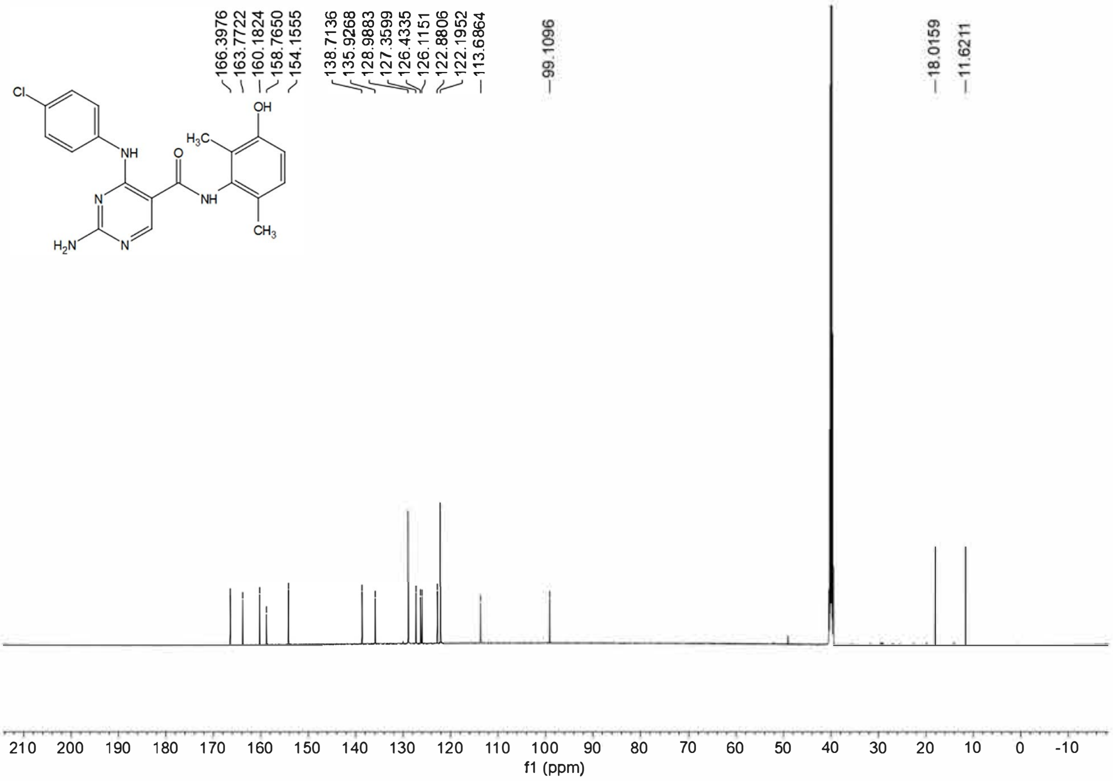

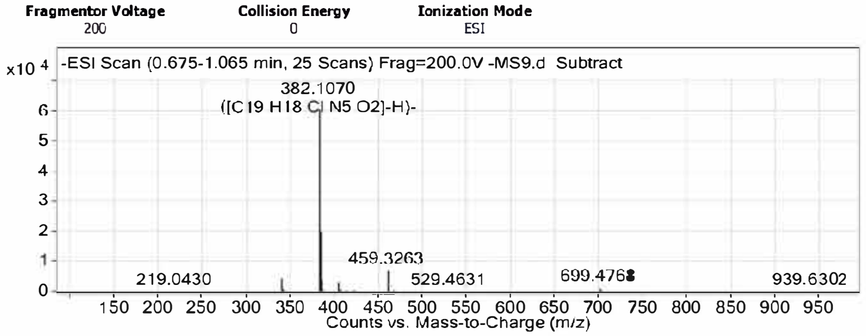

Peak List

m/z	z	Abund	Formula	Ion
339.233	1	4806.08		
340.2358	1	1264.22		
382.107	1	60729.21	C19 H18 Cl N5 O2	(M-H)-
383.1099	1	13396.27	C19 H18 Cl N5 O2	(M-H)-
384.1046	1	20290.89	C19 H18 Cl N5 O2	(M-H)-
385.1074	1	4276.01	C19 H18 Cl N5 O2	(M-H)-
404.0885	1	3421.89		
459.3263	1	7296.9		
460.3295	1	2707.55		
699.4768	1	1672.85		

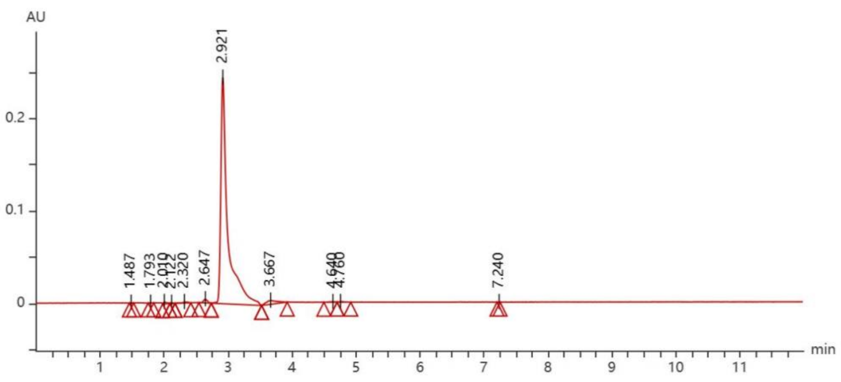

Object List

#	RT (min)	Area (µV*s)	% Area	Height (µV)	% Height
1	1.487	512.667	0.026	216.750	0.084
2	1.793	593.467	0.031	178.750	0.069
3	2.010	7707.445	0.398	1958.212	0.757
4	2.122	2926.724	0.151	989.813	0.383
5	2.320	8620.998	0.445	1185.716	0.459
6	2.647	17654.258	0.912	3864.574	1.495
7	2.921	1841322.200	95.082	244608.910	94.618
8	3.667	48269.033	2.493	4025.387	1.557
9	4.640	3972.200	0.205	569.000	0.220
10	4.760	4753.400	0.245	725.500	0.281
11	7.240	232.500	0.012	201.071	0.078

^{1}H NMR, ^{13}C NMR, HRMS and HPLC spectra of Compound MS4

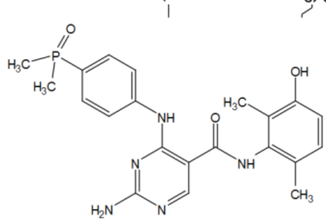

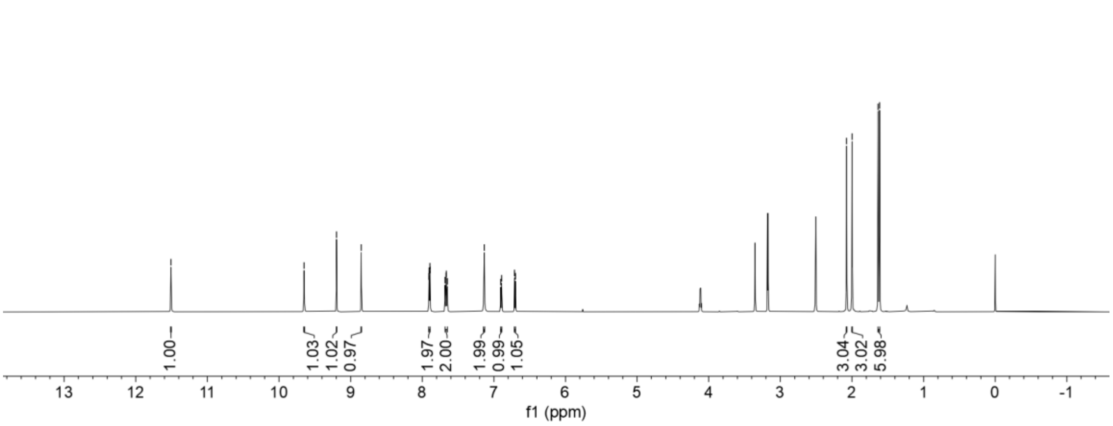

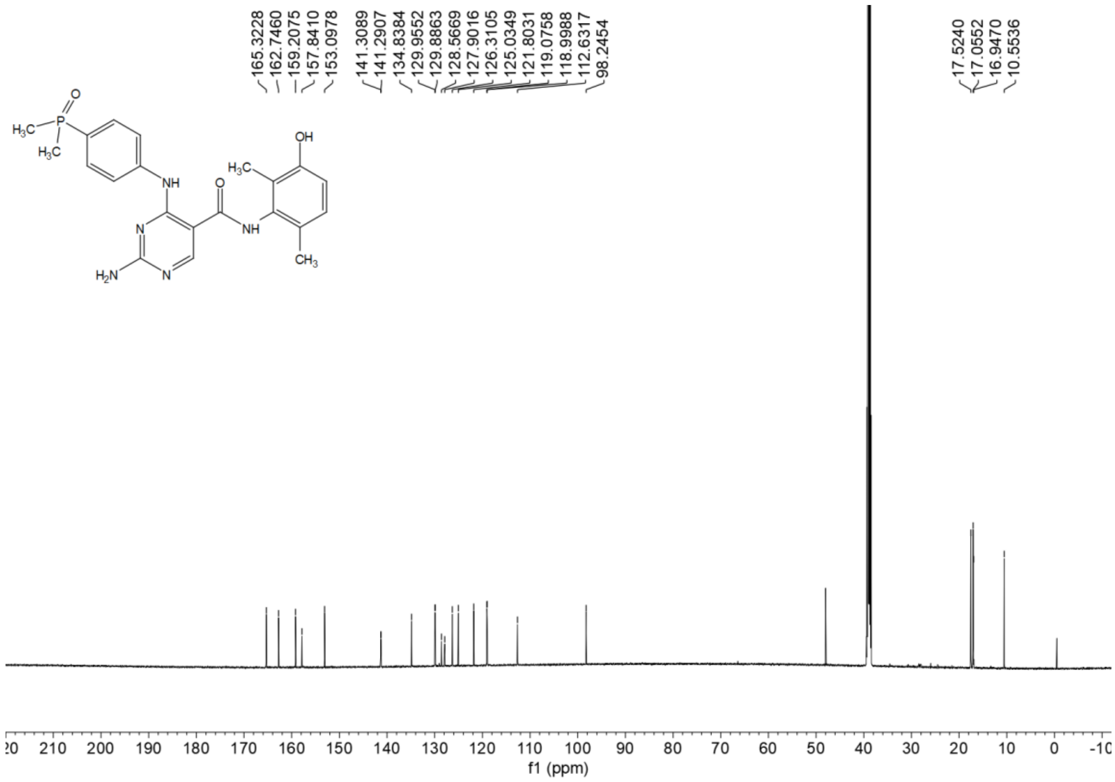

Fragmentor Voltage	Collision Energy	Ionization Mode
200	0	ESI

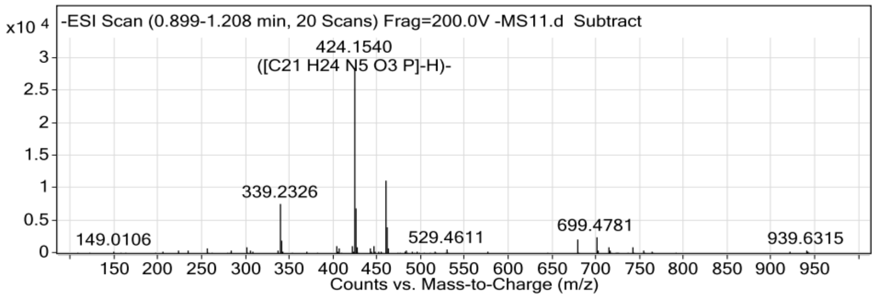

Peak List

m/z	z	Abund	Formula	Ion
339.2326	1	7624.17		
340.2367	1	2041.9		
403.2639	1	1217.24		
424.154	1	28487.9	C21 H24 N5 O3 P	(M-H)-
425.1568	1	6993.06	C21 H24 N5 O3 P	(M-H)-
459.3263	1	11163.77		
460.3297	1	4025.32		
677.4966	1	2273.99		
699.4781	1	2483.48		
700.4821	1	1400.33		

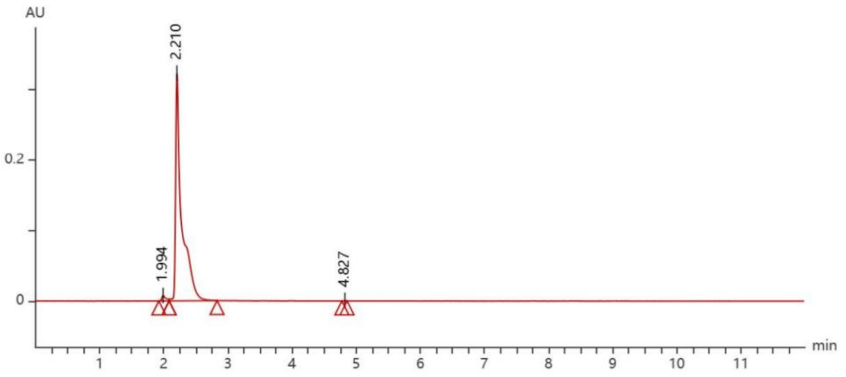

Object List

#	RT (min)	Area (µV*s)	% Area	Height (µV)	% Height
1	1.994	34225.512	1.581	7562.538	2.270
2	2.210	2130401.588	98.402	325442.878	97.671
3	4.827	367.267	0.017	197.375	0.059

^{1}H NMR, ^{13}C NMR, HRMS and HPLC spectra of Compound MS5

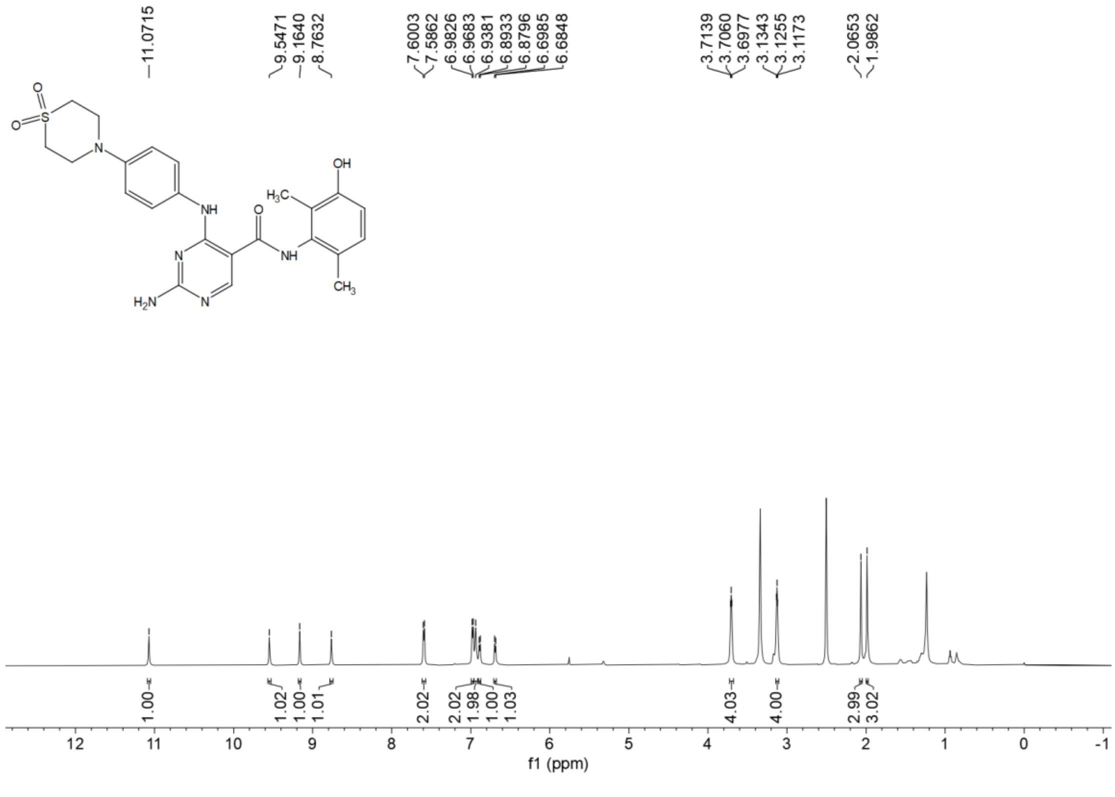

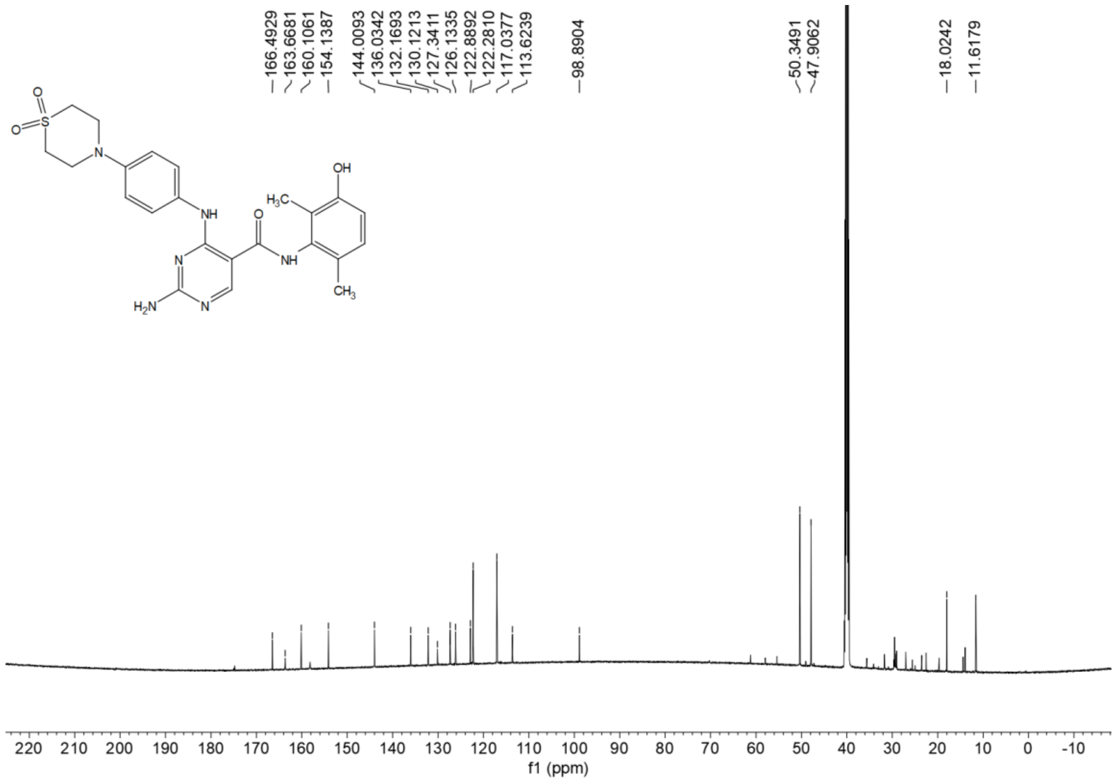

Fragmentor Voltage	Collision Energy	Ionization Mode
200	0	ESI

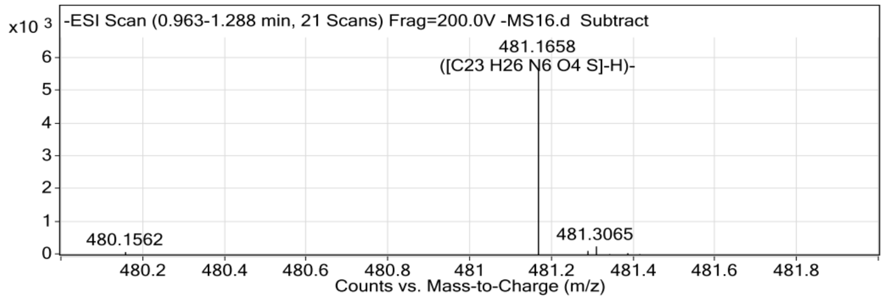

Peak List

m/z	z	Abund	Formula	Ion
339.2326	1	5186.4		
340.2364	1	1365.94		
403.2633	1	746.68		
459.3259	1	6613.19		
460.329	1	2348.54		
481.1658	1	5699.3	C23 H26 N6 O4 S	(M-H)-
482.1676	1	1774.41	C23 H26 N6 O4 S	(M-H)-
677.4955	1	1085.19		
699.4774	1	1461.86		
700.4806	1	836.06		

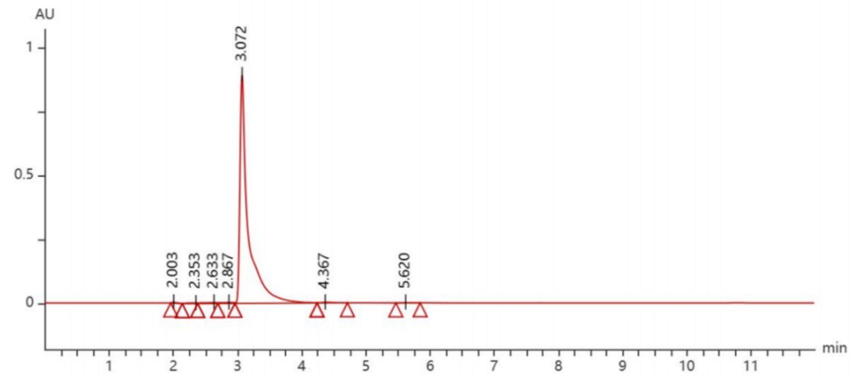

Object List

#	RT (min)	Area (µV*s)	% Area	Height (µV)	% Height
1	2.003	20336.433	0.249	3903.998	0.428
2	2.353	30793.239	0.377	2748.383	0.301
3	2.633	52375.807	0.641	3399.262	0.373
4	2.867	49747.246	0.609	3822.619	0.419
5	3.072	7971800.225	97.542	894941.593	98.084
6	4.367	37476.424	0.459	2630.877	0.288
7	5.620	10148.600	0.124	972.346	0.107

^{1}H NMR, ^{13}C NMR, HRMS and HPLC spectra of Compound MS6

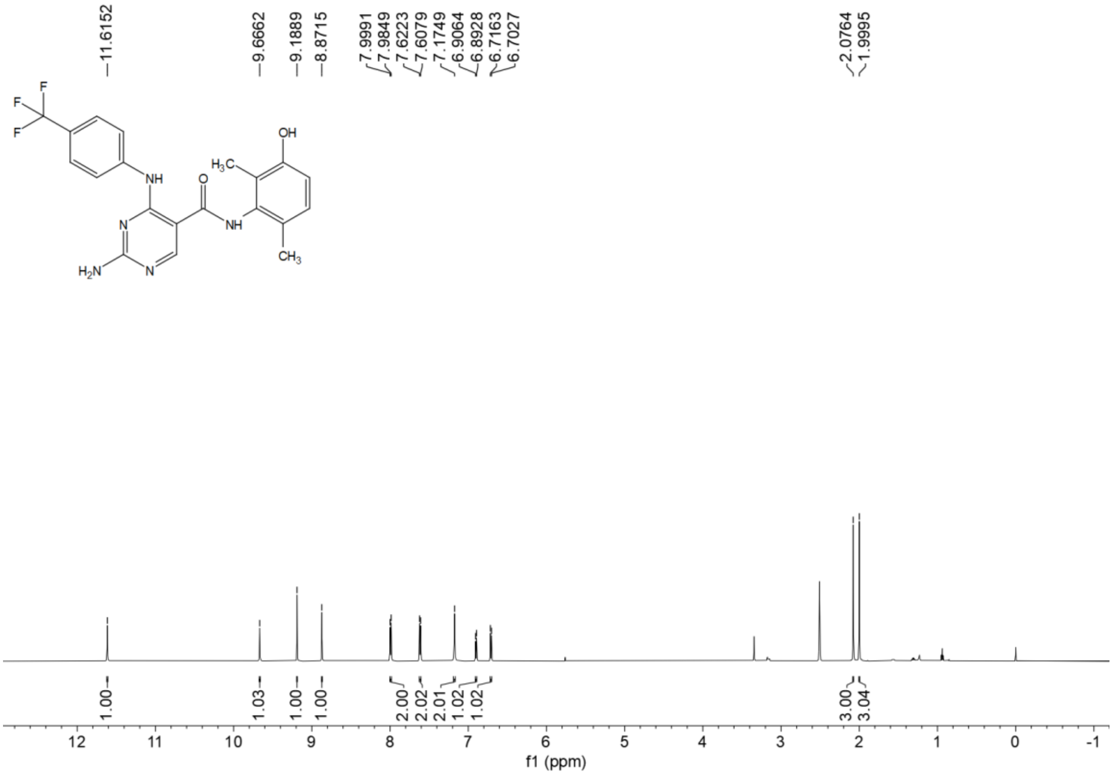

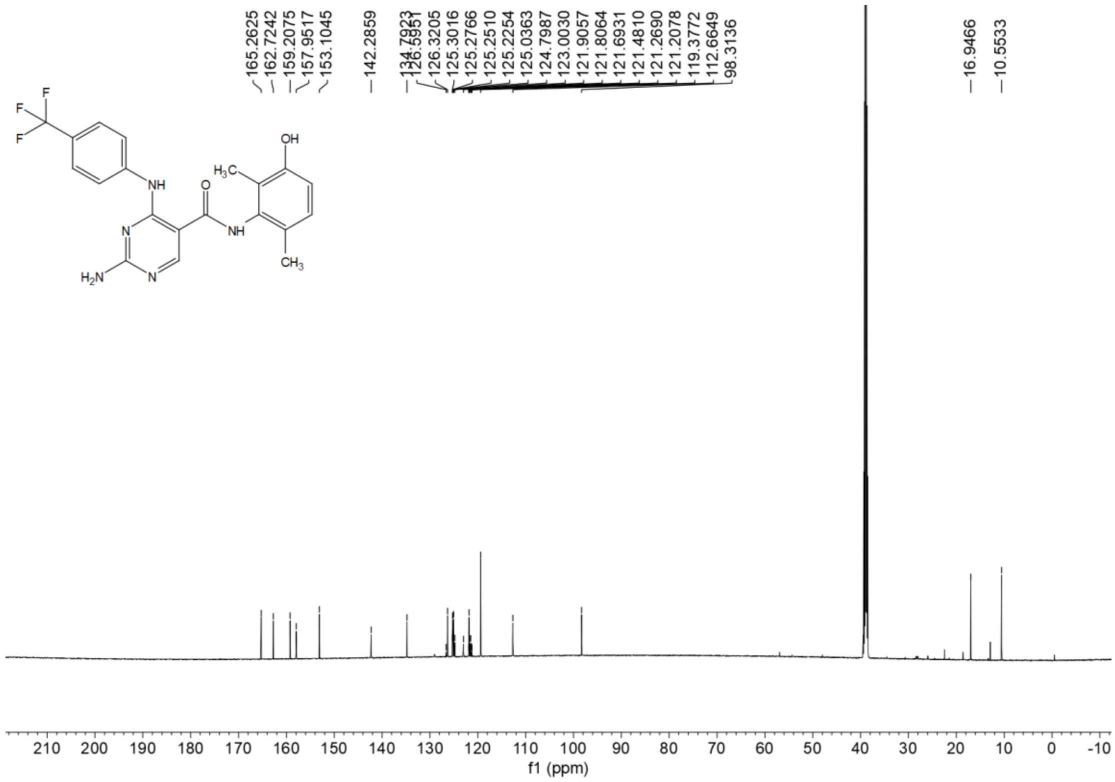

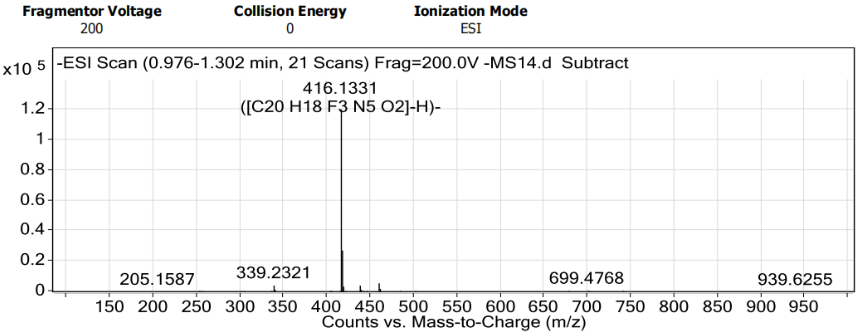

Peak List

m/z	z	Abund	Formula	Ion
339.2321	1	4616.71		
340.2357	1	1191.62		
416.1331	1	120326.87	C20 H18 F3 N5 O2	(M-H)-
417.1359	1	27328.68	C20 H18 F3 N5 O2	(M-H)-
418.1386	1	3666.74	C20 H18 F3 N5 O2	(M-H)-
438.1143	1	4518.5		
439.1185	1	1219.47		
459.3258	1	5540.13		
460.329	1	1915.89		
699.4768	1	1053.02		

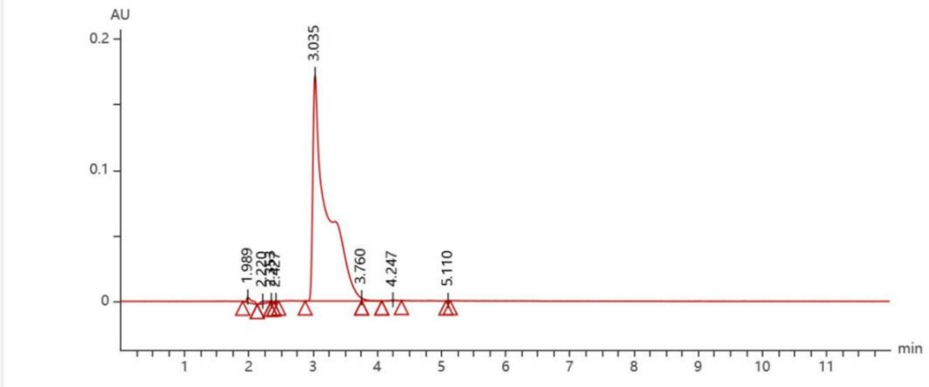

Object List

#	RT (min)	Area (µV*s)	% Area	Height (µV)	% Height
1	1.989	24101.133	0.888	3970.558	2.171
2	2.220	16457.006	0.607	1888.920	1.033
3	2.353	2834.880	0.105	1285.220	0.703
4	2.427	1707.564	0.063	550.210	0.301
5	3.035	2652277.888	97.777	172288.362	94.206
6	3.760	10805.645	0.398	2215.693	1.212
7	4.247	4011.200	0.148	521.152	0.285
8	5.110	393.400	0.015	164.860	0.090

^{1}H NMR, ^{13}C NMR, HRMS and HPLC spectra of Compound MS7

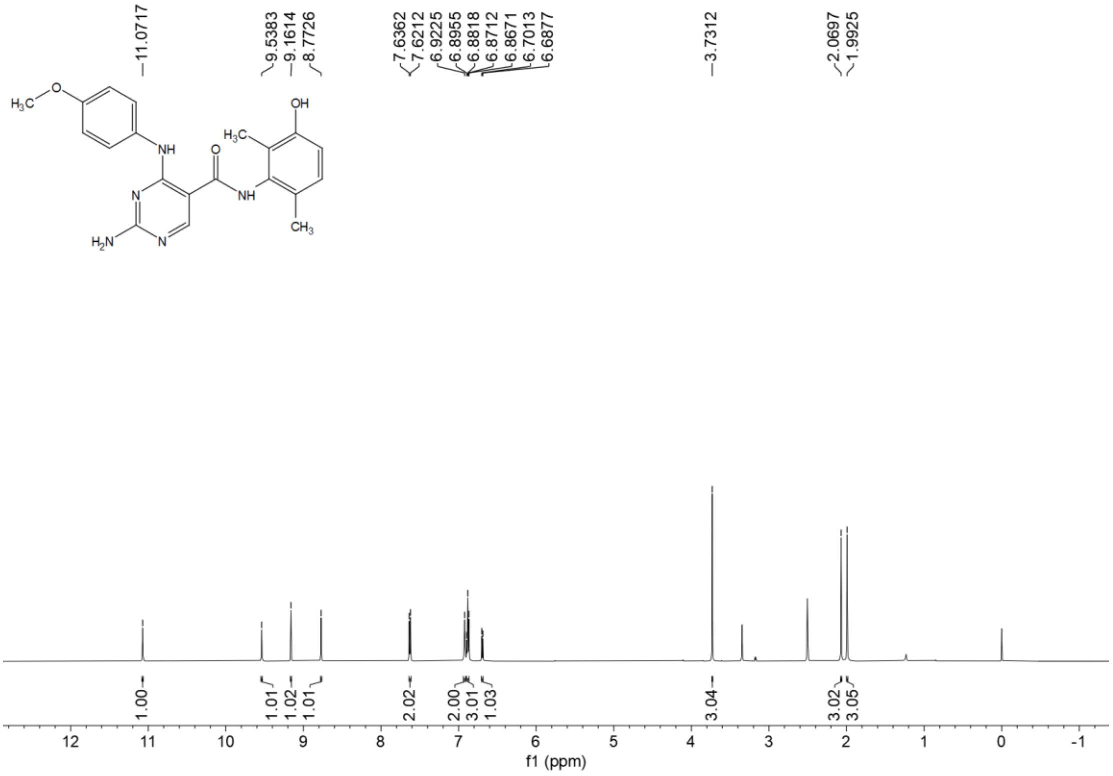

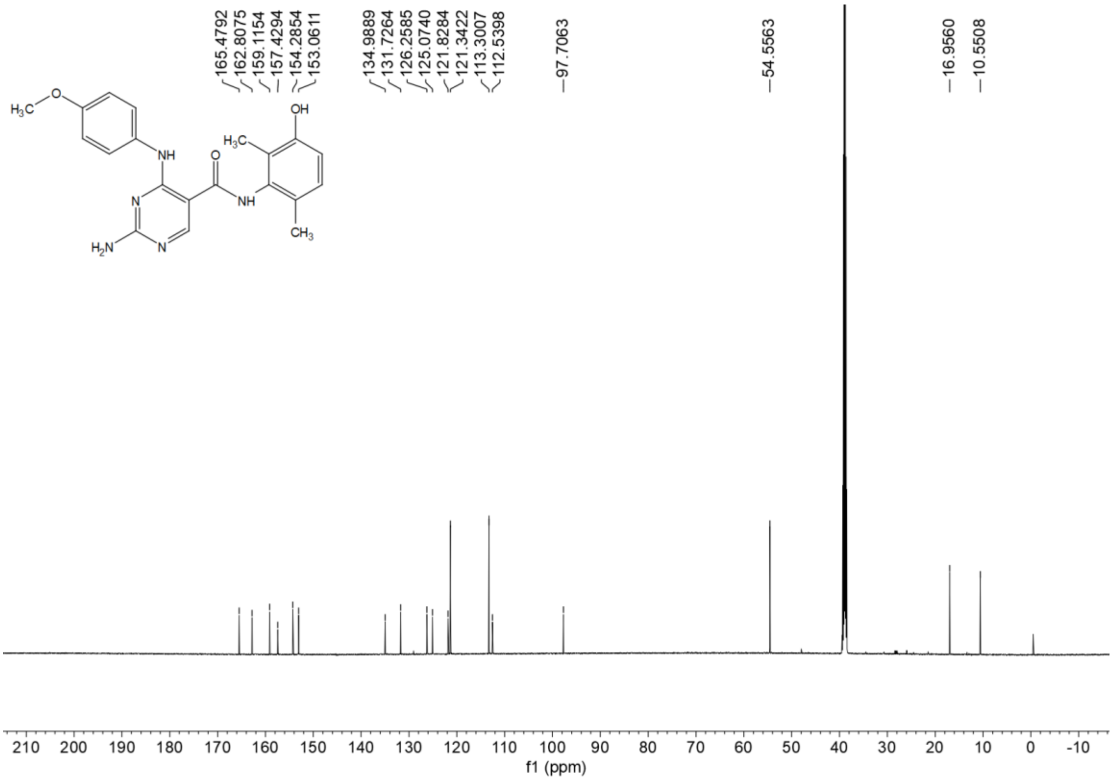

Fragmentor Voltage	Collision Energy	Ionization Mode
200	0	ESI

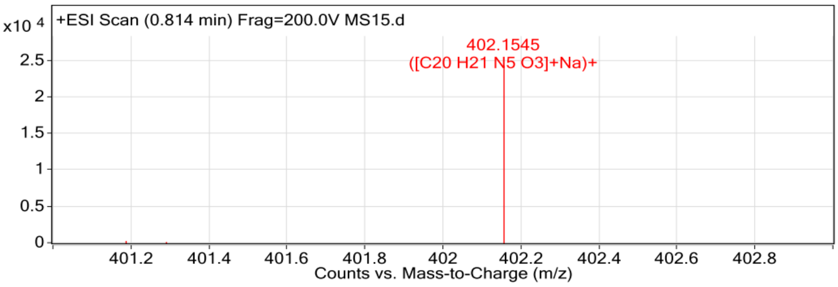

Peak List

m/z	z	Abund	Formula	Ion
301.1415	1	33743.83		
302.1452	1	5813.23		
306.2765	1	9202.16		
334.3092	1	7403.99		
360.3245	1	14621.36		
363.1567	1	5439.9		
402.1545	1	24404.25	C20 H21 N5 O3	(M+Na)+
403.1573	1	5820.21	C20 H21 N5 O3	(M+Na)+
695.4865	1	4855.02		
781.3173	1	10726.2		

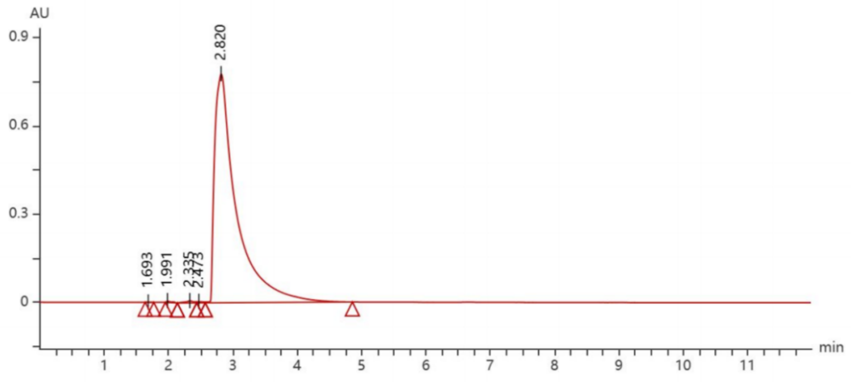

Object List

#	RT (min)	Area (µV*s)	% Area	Height (µV)	% Height
1	1.693	1121.900	0.006	320.042	0.040
2	1.991	25294.471	0.137	4327.518	0.546
3	2.335	64853.604	0.352	6893.767	0.870
4	2.473	27502.347	0.149	3935.824	0.497
5	2.820	18281536.191	99.355	776818.250	98.047

^{1}H NMR, ^{13}C NMR, HRMS and HPLC spectra of Compound MS8

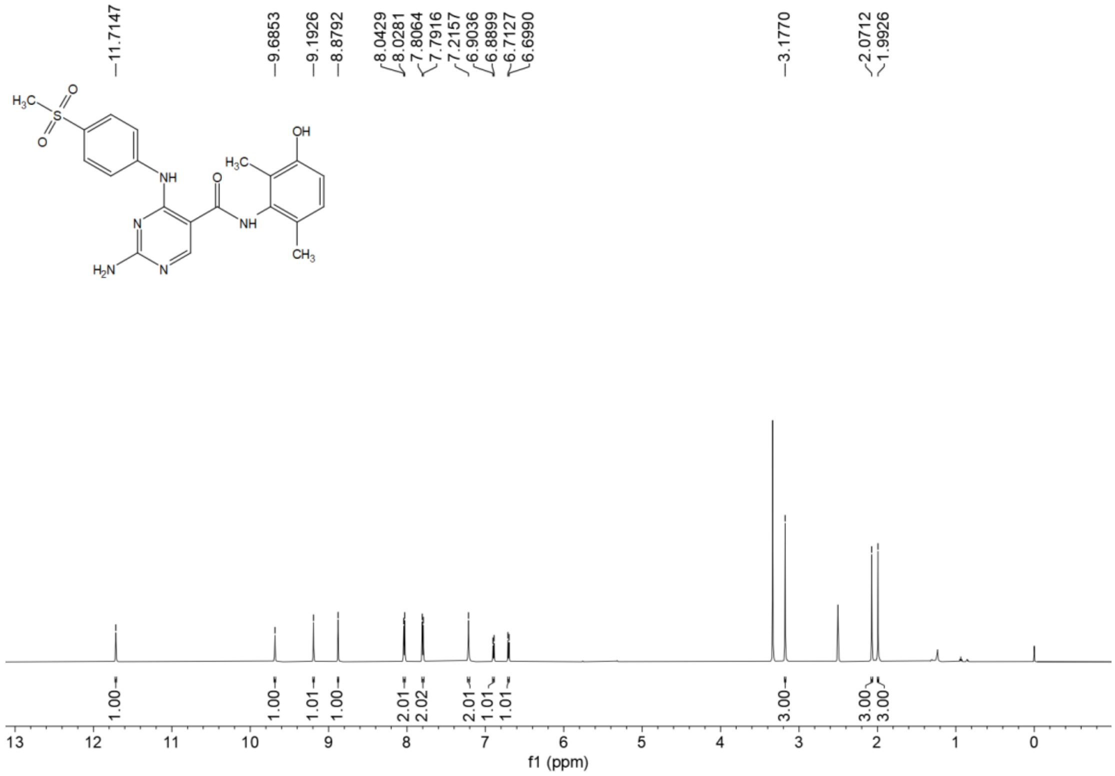

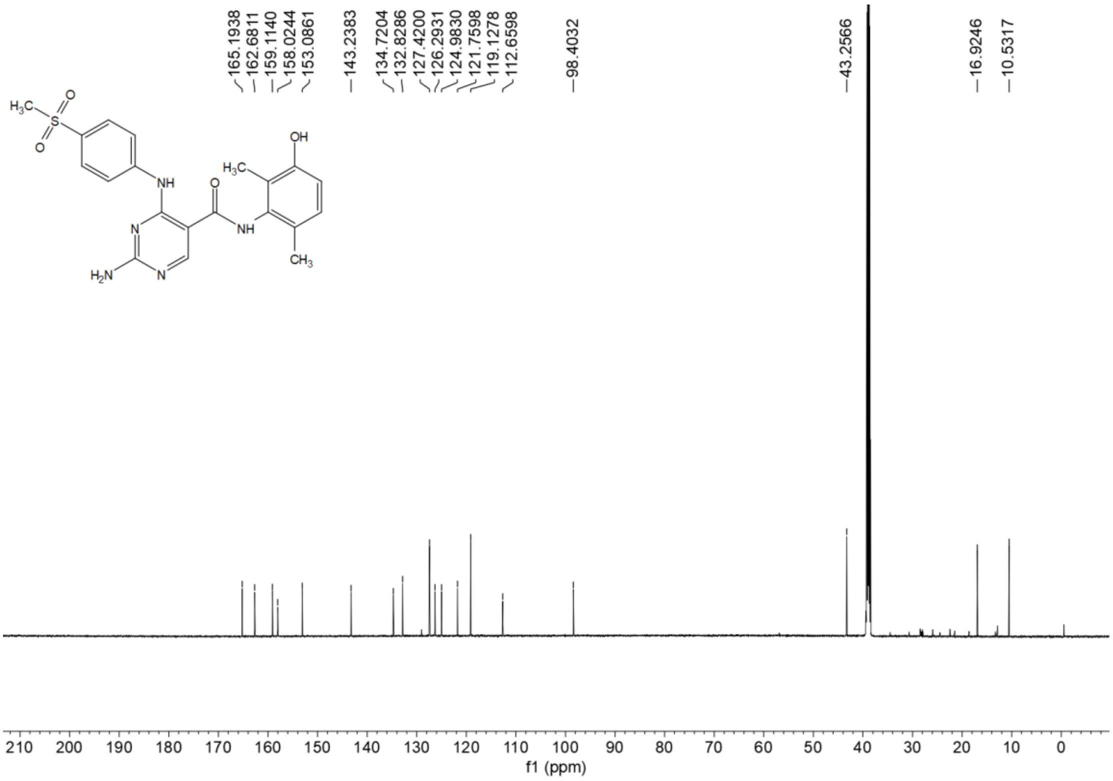

Fragmentor Voltage	Collision Energy	Ionization Mode
200	0	ESI

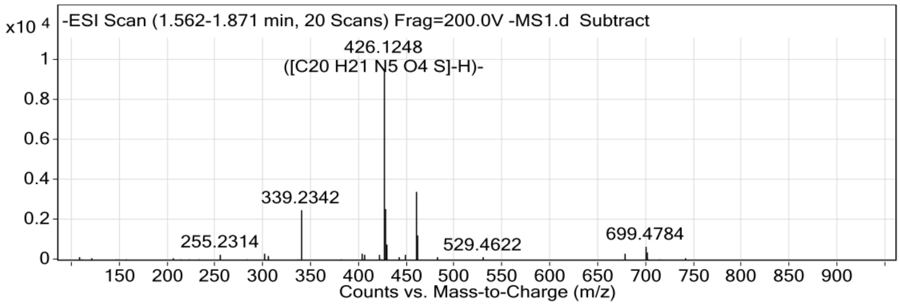

Peak List

m/z	z	Abund	Formula	Ion
301.1597	1	367.5		
339.2342	1	2506.3		
340.2368	1	674.95		
426.1248	1	9543.69	C20 H21 N5 O4 S	(M-H)-
427.1275	1	2574.32	C20 H21 N5 O4 S	(M-H)-
428.1266	1	816.25	C20 H21 N5 O4 S	(M-H)-
459.3281	1	3410.14		
460.3309	1	1240.11		
699.4784	1	687.42		
700.4825	1	400.78		

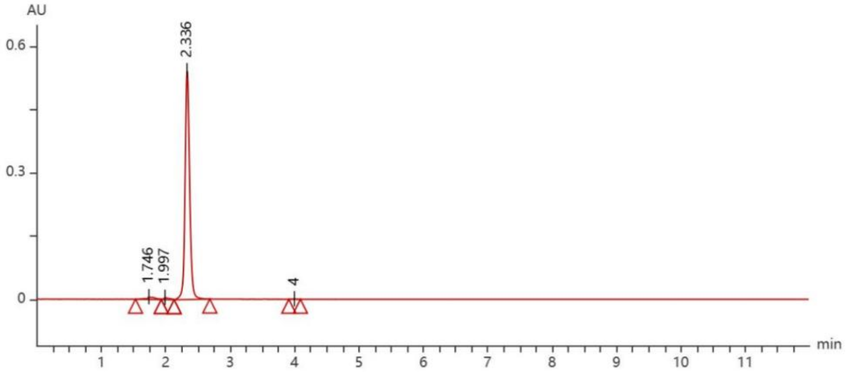

Object List

#	RT (min)	Area (µV*s)	% Area	Height (µV)	% Height
1	1.746	54222.267	1.967	5590.284	1.009
2	1.997	27875.133	1.011	4527.138	0.817
3	2.336	2672344.275	96.953	543721.156	98.113
4	4.000	1884.167	0.068	342.630	0.062

^{1}H NMR, ^{13}C NMR, HRMS and HPLC spectra of Compound MS9

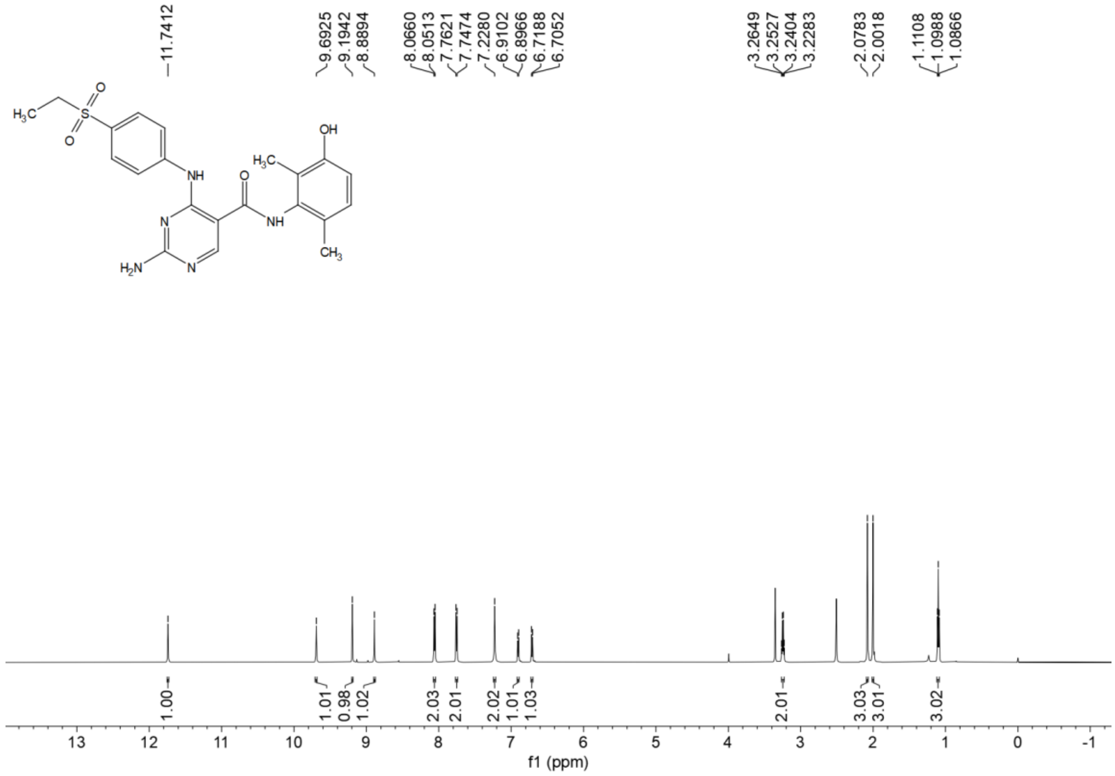

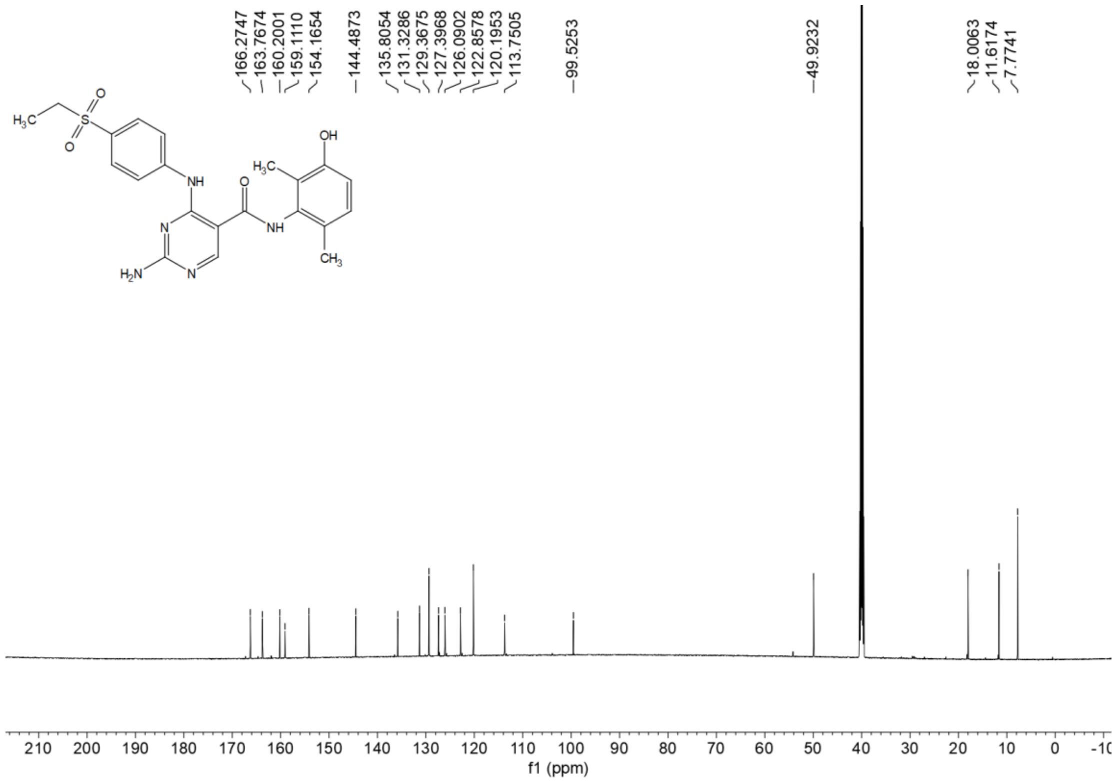

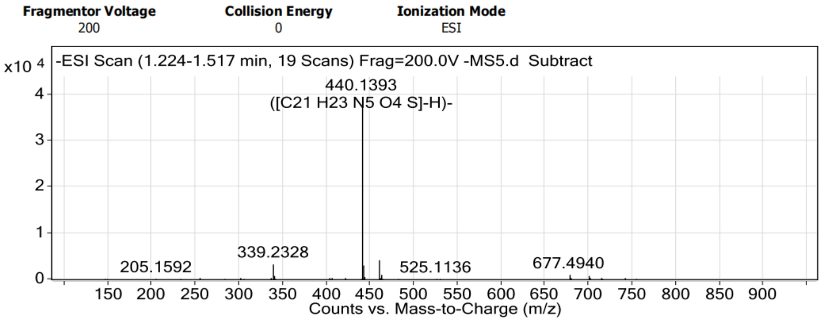

Peak List

m/z	z	Abund	Formula	Ion
339.2328	1	3492.75		
340.2358	1	948.86		
440.1393	1	37742.25	C21 H23 N5 O4 S	(M-H)-
441.1419	1	9544.24	C21 H23 N5 O4 S	(M-H)-
442.1395	1	3133.32	C21 H23 N5 O4 S	(M-H)-
459.326	1	4368.77		
460.33	1	1646.49		
462.1205	1	1068.93		
677.494	1	1132.48		
699.4779	1	876.03		

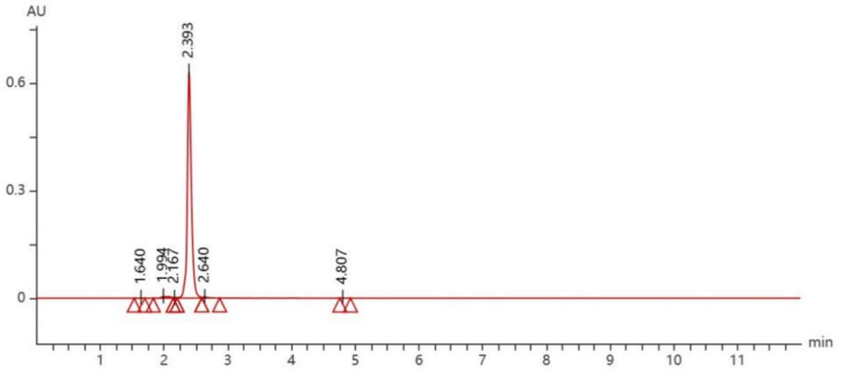

Object List

#	RT (min)	Area (µV*s)	% Area	Height (µV)	% Height
1	1.640	1745.817	0.061	408.380	0.064
2	1.994	44531.137	1.549	5056.857	0.787
3	2.167	6119.954	0.213	1759.699	0.274
4	2.393	2799734.397	97.362	631791.404	98.295
5	2.640	21807.287	0.758	3406.061	0.530
6	4.807	1663.433	0.058	330.650	0.051

^{1}H NMR, ^{13}C NMR, HRMS and HPLC spectra of Compound MS10

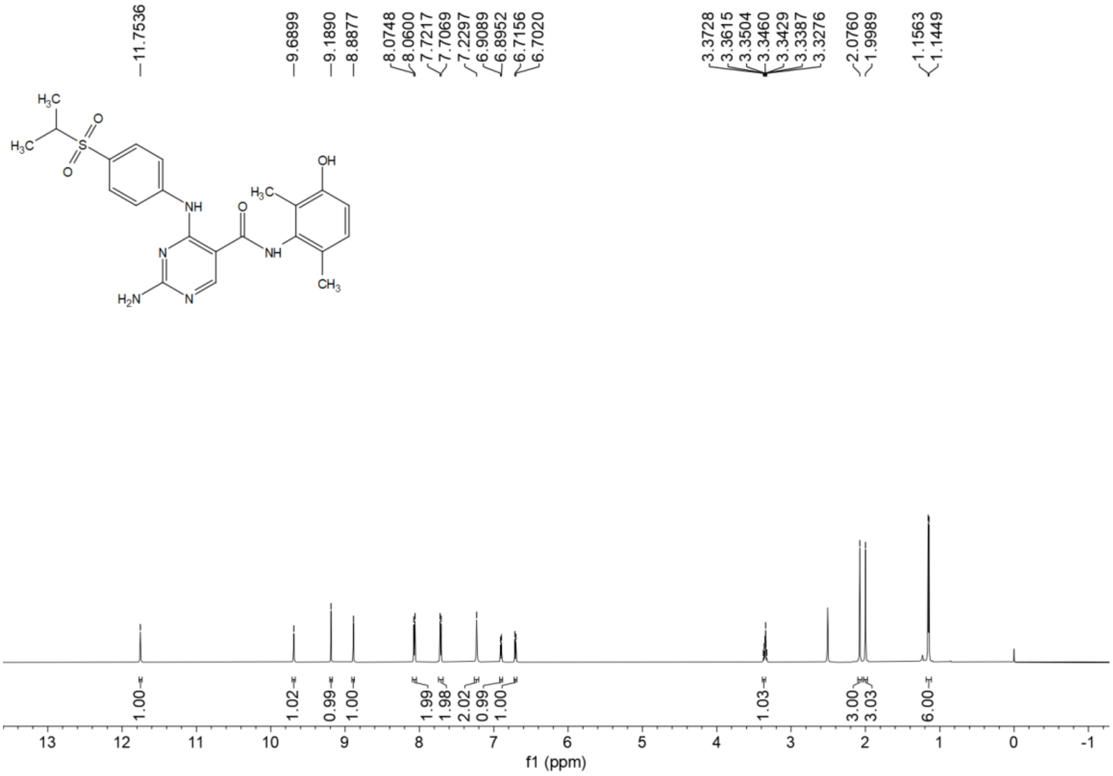

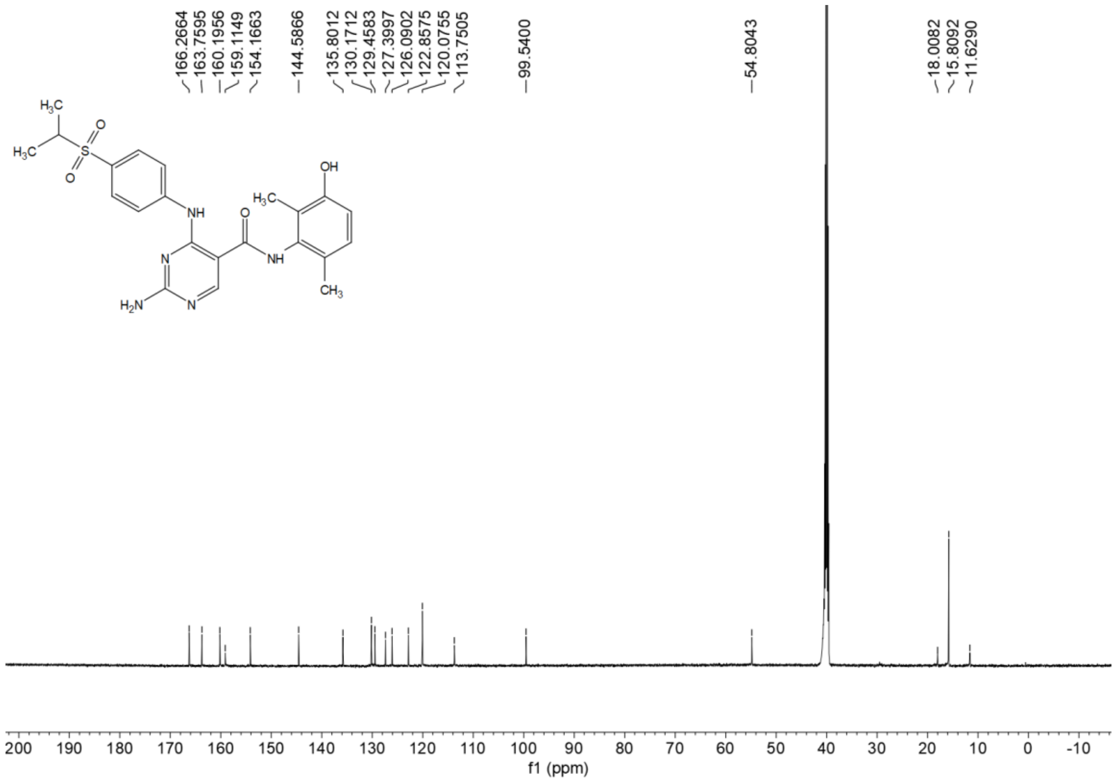

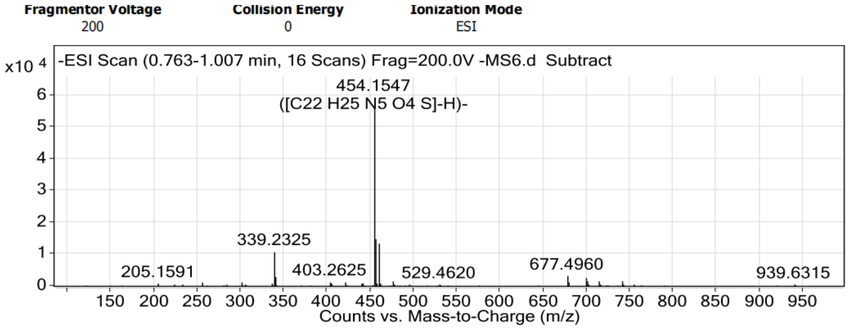

Peak List

m/z	z	Abund	Formula	Ion
339.2325	1	10976.71		
340.2361	1	2896.88		
454.1547	1	56535.53	C22 H25 N5 O4 S	(M-H)-
455.1574	1	14835.99	C22 H25 N5 O4 S	(M-H)-
456.1552	1	4883.54	C22 H25 N5 O4 S	(M-H)-
459.3263	1	13388.5		
460.3295	1	4775.05		
476.1363	1	1840.48		
677.496	1	3265.03		
699.4766	1	2730.19		

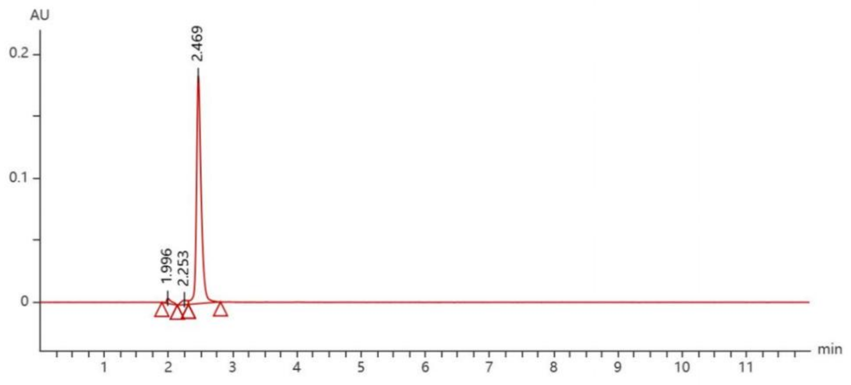

Object List

#	RT (min)	Area (µV*s)	% Area	Height (µV)	% Height
1	1.996	23113.578	2.364	4106.275	2.142
2	2.253	24906.925	2.547	3418.300	1.783
3	2.469	929700.642	95.089	184220.848	96.076

^{1}H NMR, ^{13}C NMR, HRMS and HPLC spectra of Compound MS11

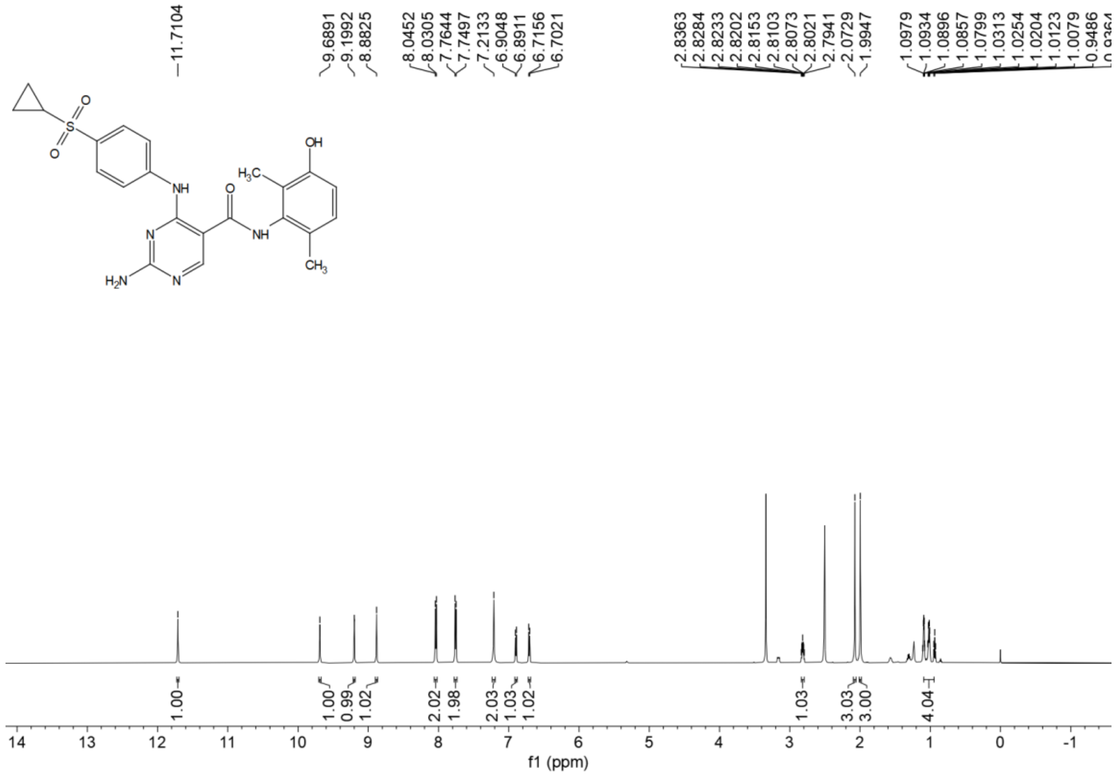

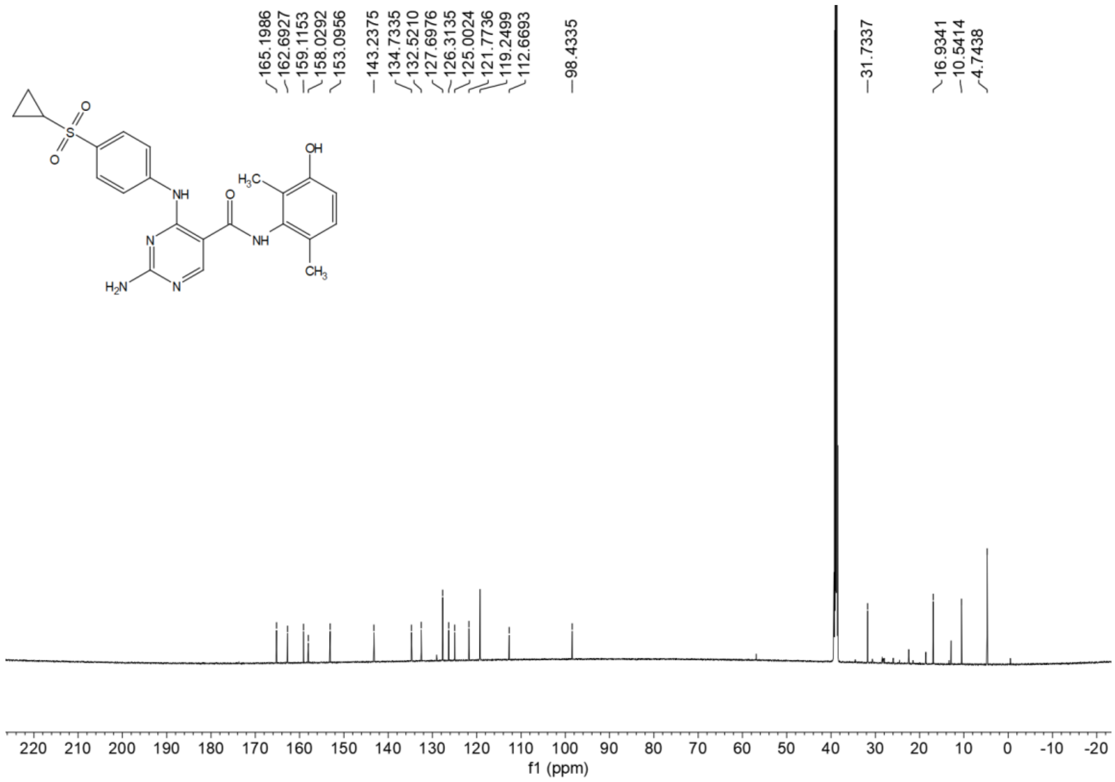

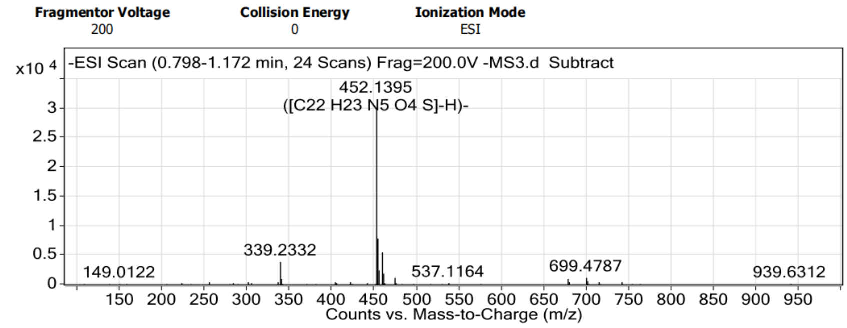

Peak List

m/z	z	Abund	Formula	Ion
339.2332	1	3961.25		
340.236	1	1037.32		
452.1395	1	30164	C22 H23 N5 O4 S	(M-H)-
453.1422	1	7999.14	C22 H23 N5 O4 S	(M-H)-
454.1406	1	2515.75	C22 H23 N5 O4 S	(M-H)-
459.327	1	5576.79		
460.3308	1	1995.3		
474.1205	1	1252.14		
677.4951	1	1108.42		
699.4787	1	1249.13		

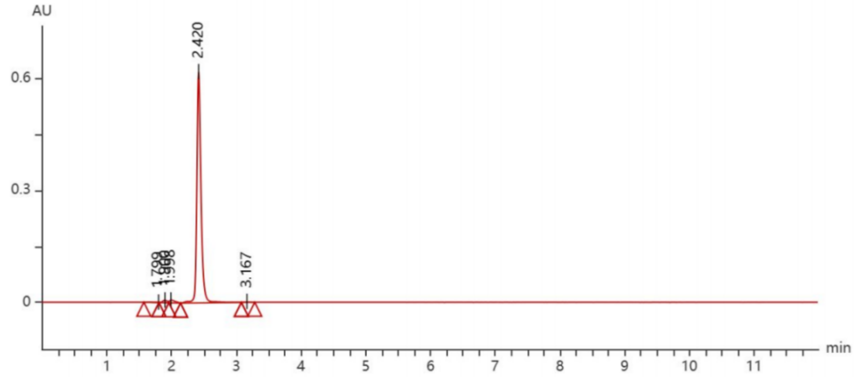

Object List

#	RT (min)	Area (µV*s)	% Area	Height (µV)	% Height
1	1.799	10112.028	0.354	995.961	0.157
2	1.900	34960.611	1.223	6286.821	0.992
3	1.998	44597.803	1.560	7402.484	1.167
4	2.420	2761624.213	96.615	618375.375	97.528
5	3.167	7097.921	0.248	987.625	0.156

^{1}H NMR, ^{13}C NMR, HRMS and HPLC spectra of Compound MS12

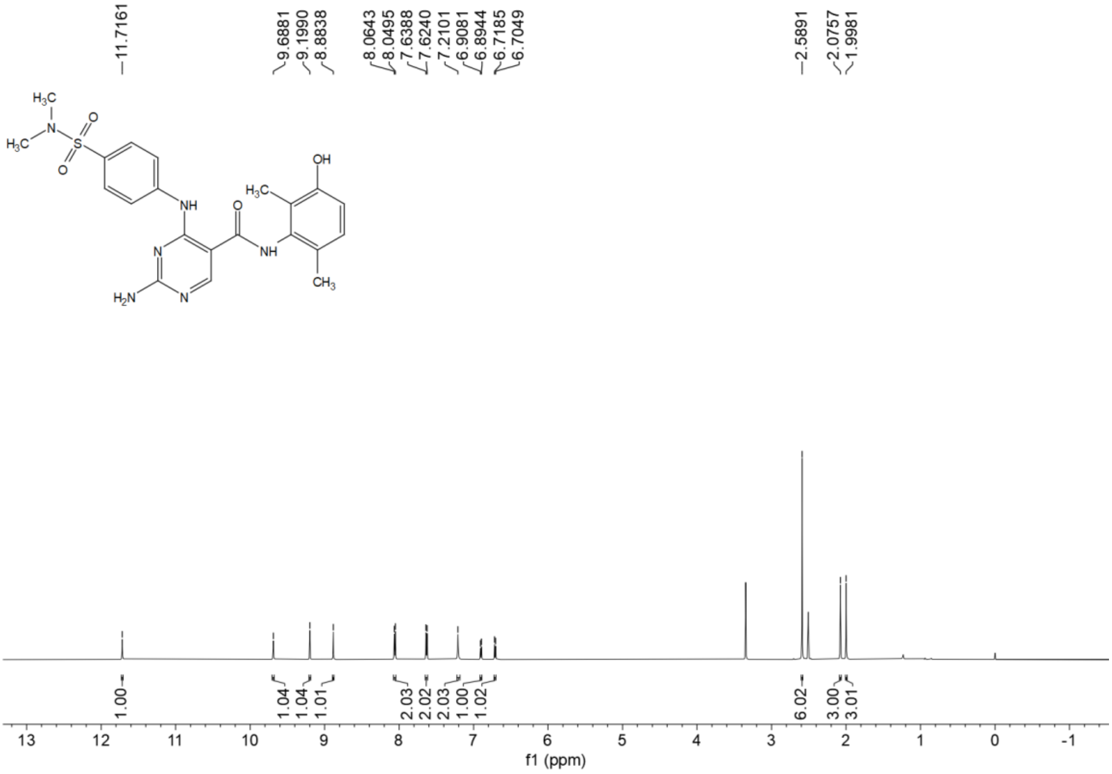

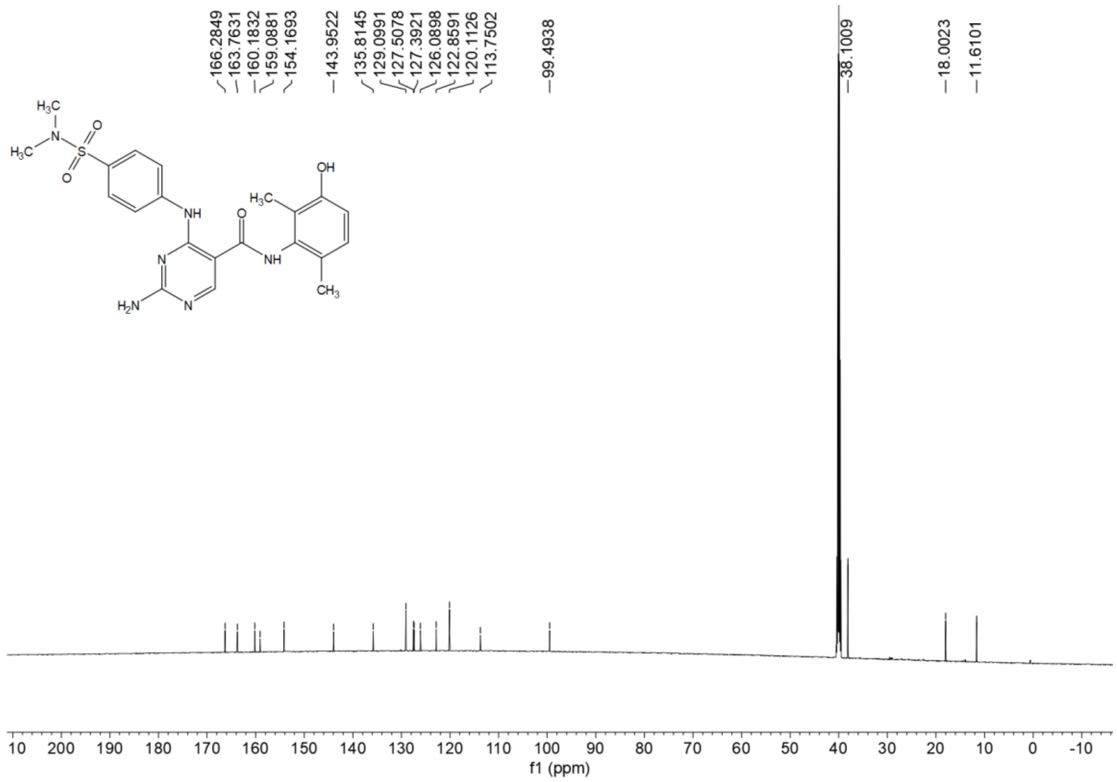

Fragmentor Voltage	Collision Energy	Ionization Mode
200	0	ESI

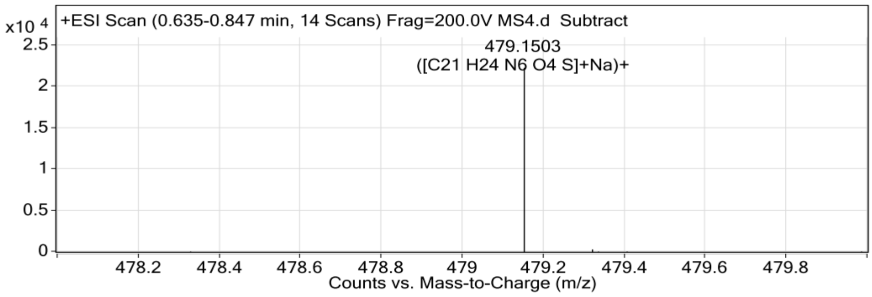

Peak List

m/z	z	Abund	Formula	Ion
306.2789	1	5472.76		
334.3098	1	5040.11		
360.3261	1	9301.6		
363.1586	1	3432.32		
363.2298		3326.23		
472.1224	1	23912.63		
473.1251	1	6344.99		
479.1503	1	22339.67	C21 H24 N6 O4 S	(M+Na)+
480.154	1	5850.25	C21 H24 N6 O4 S	(M+Na)+
495.1247	1	4297.25		

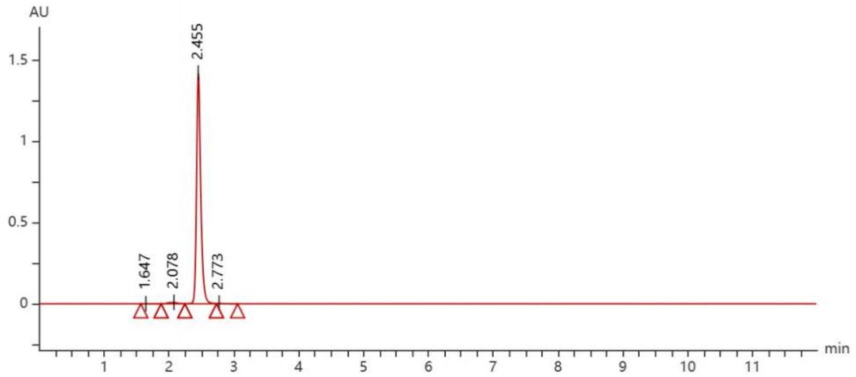

Object List

#	RT (min)	Area (µV*s)	% Area	Height (µV)	% Height
1	1.647	5055.606	0.077	724.973	0.051
2	2.078	88858.500	1.358	7547.754	0.527
3	2.455	6427571.339	98.265	1421083.442	99.230
4	2.773	19604.764	0.300	2749.721	0.192

^{1}H NMR, ^{13}C NMR, HRMS and HPLC spectra of Compound MS13

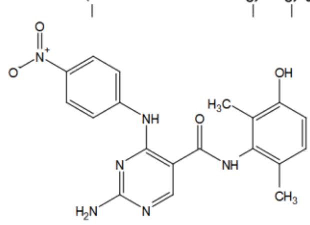

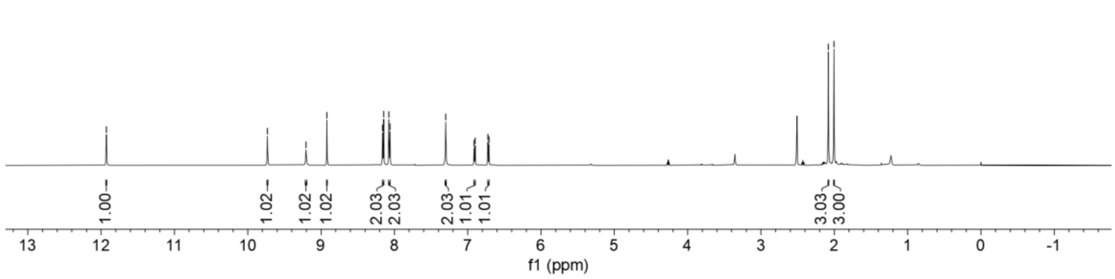

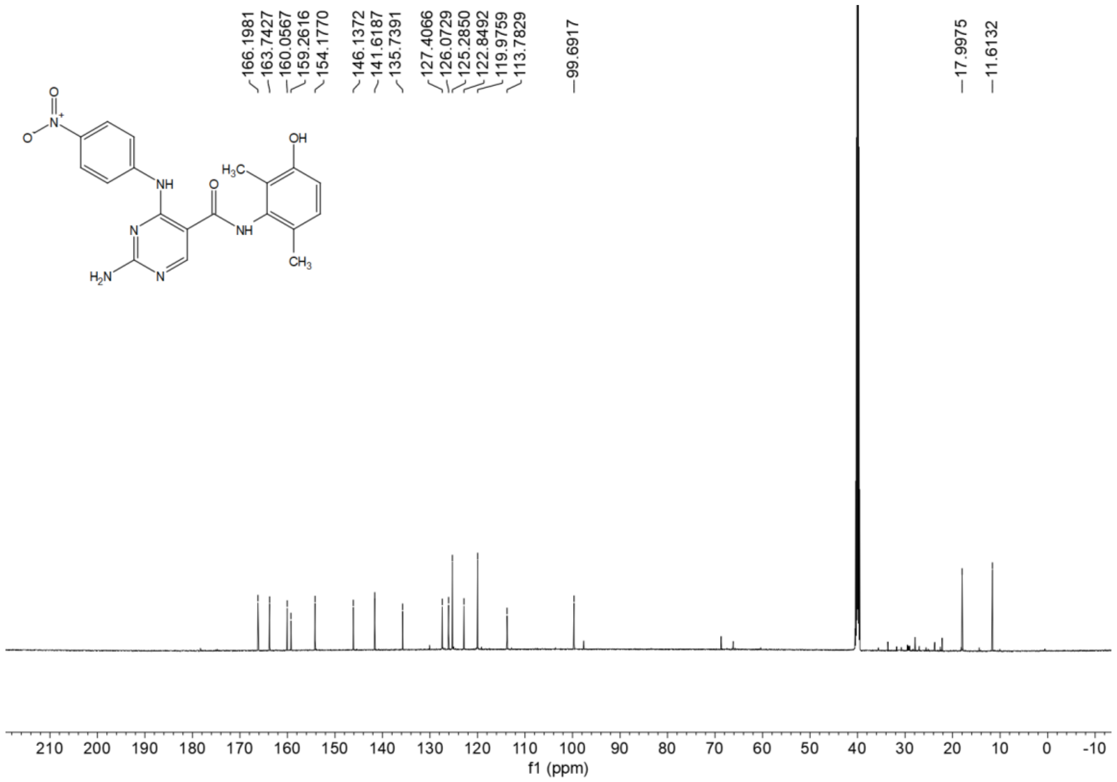

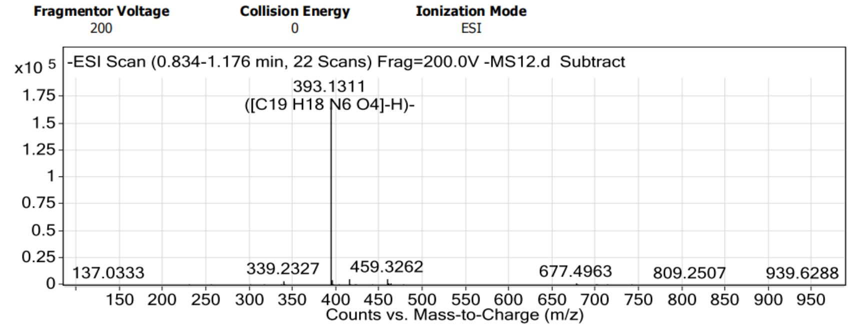

Peak List

m/z	z	Abund	Formula	Ion
339.2327	1	4390.58		
393.1311	1	165721.59	C19 H18 N6 O4	(M-H)-
394.1337	1	35818.5	C19 H18 N6 O4	(M-H)-
395.1362	1	5446.33	C19 H18 N6 O4	(M-H)-
415.1127	1	5768.79		
416.1159	1	1359.2		
459.3262	1	6191.59		
460.3297	1	2139.96		
463.1726	1	1487.17		
677.4963	1	1925.97		

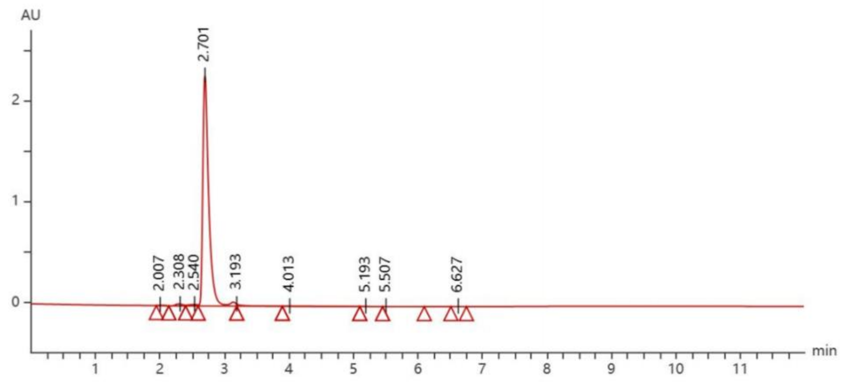

Object List

#	RT (min)	Area (µV*s)	% Area	Height (µV)	% Height
1	2.007	37373.925	0.247	5384.397	0.229
2	2.308	158684.916	1.049	19019.023	0.810
3	2.540	136555.378	0.903	14224.938	0.606
4	2.701	14599531.289	96.510	2286499.148	97.342
5	3.193	158870.486	1.050	21228.721	0.904
6	4.013	29021.340	0.192	1775.086	0.076
7	5.193	2705.842	0.018	252.486	0.011
8	5.507	2250.016	0.015	182.825	0.008
9	6.627	2500.967	0.017	374.382	0.016

^{1}H NMR, ^{13}C NMR, HRMS and HPLC spectra of Compound MS14

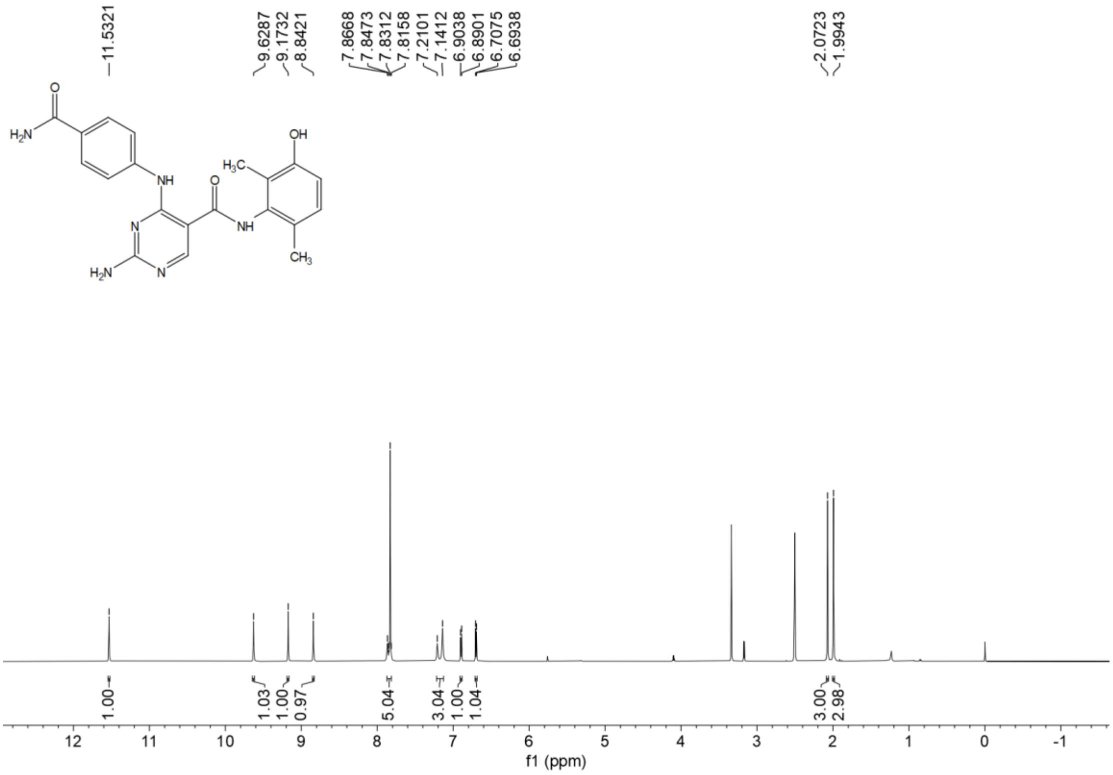

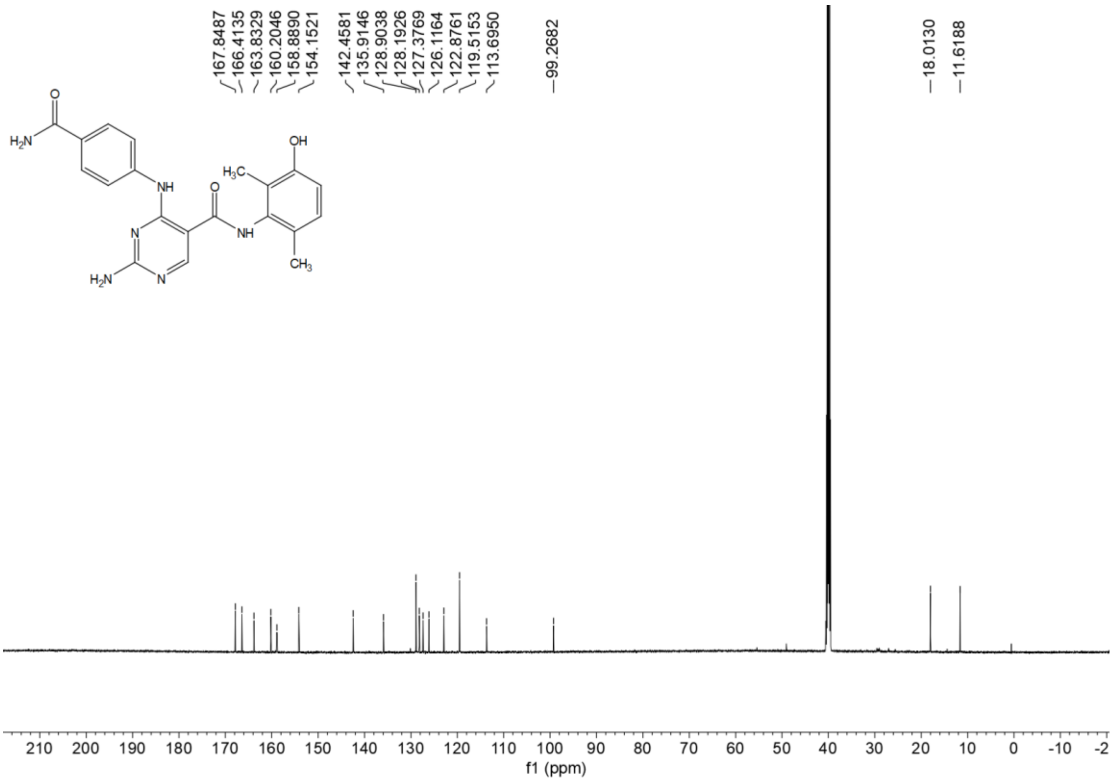

Fragmentor Voltage	Collision Energy	Ionization Mode
200	0	ESI

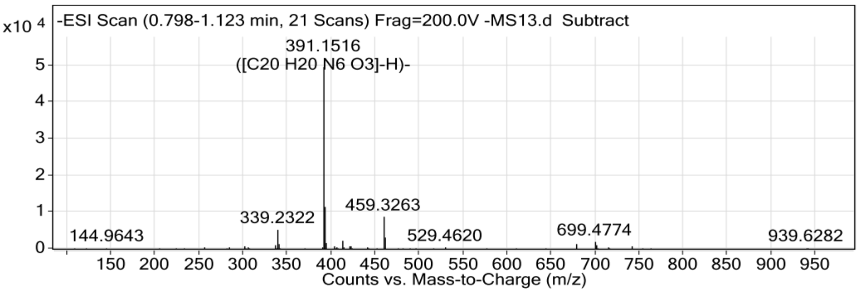

Peak List

m/z	z	Abund	Formula	Ion
339.2322	1	5359.01		
340.2354	1	1448.42		
391.1516	1	49697.08	C20 H20 N6 O3	(M-H)-
392.1544	1	11602.12	C20 H20 N6 O3	(M-H)-
393.1569	1	1779.61	C20 H20 N6 O3	(M-H)-
413.1332	1	2311.48		
459.3263	1	8818.26		
460.3298	1	3139.12		
677.4944	1	1476.13		
699.4774	1	2013.12		

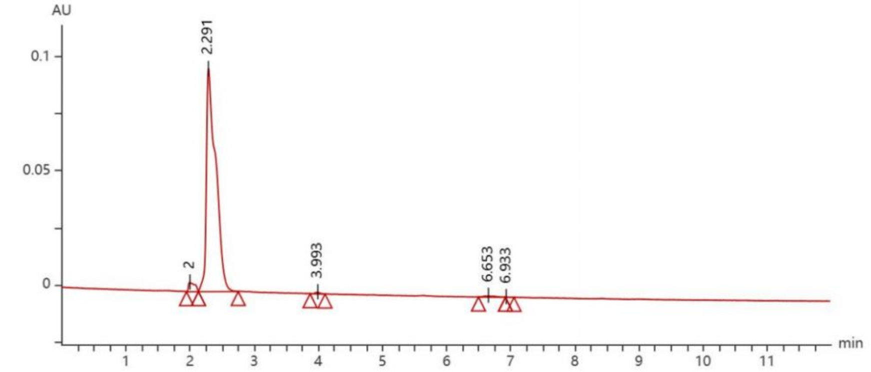

Object List

#	RT (min)	Area (µV*s)	% Area	Height (µV)	% Height
1	2.000	28889.566	2.777	4085.721	3.925
2	2.291	998734.817	96.011	98530.596	94.644
3	3.993	4253.625	0.409	801.314	0.770
4	6.653	7792.903	0.749	566.723	0.544
5	6.933	562.556	0.054	122.717	0.118

^{1}H NMR, ^{13}C NMR, HRMS and HPLC spectra of Compound MS15

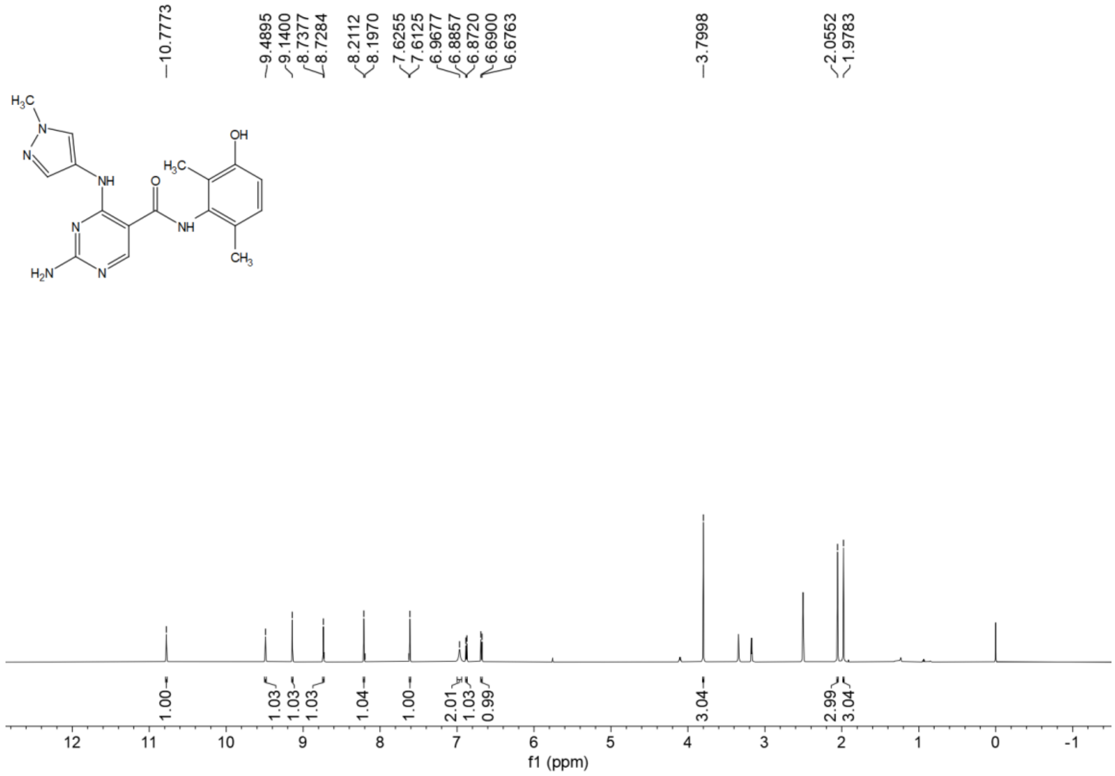

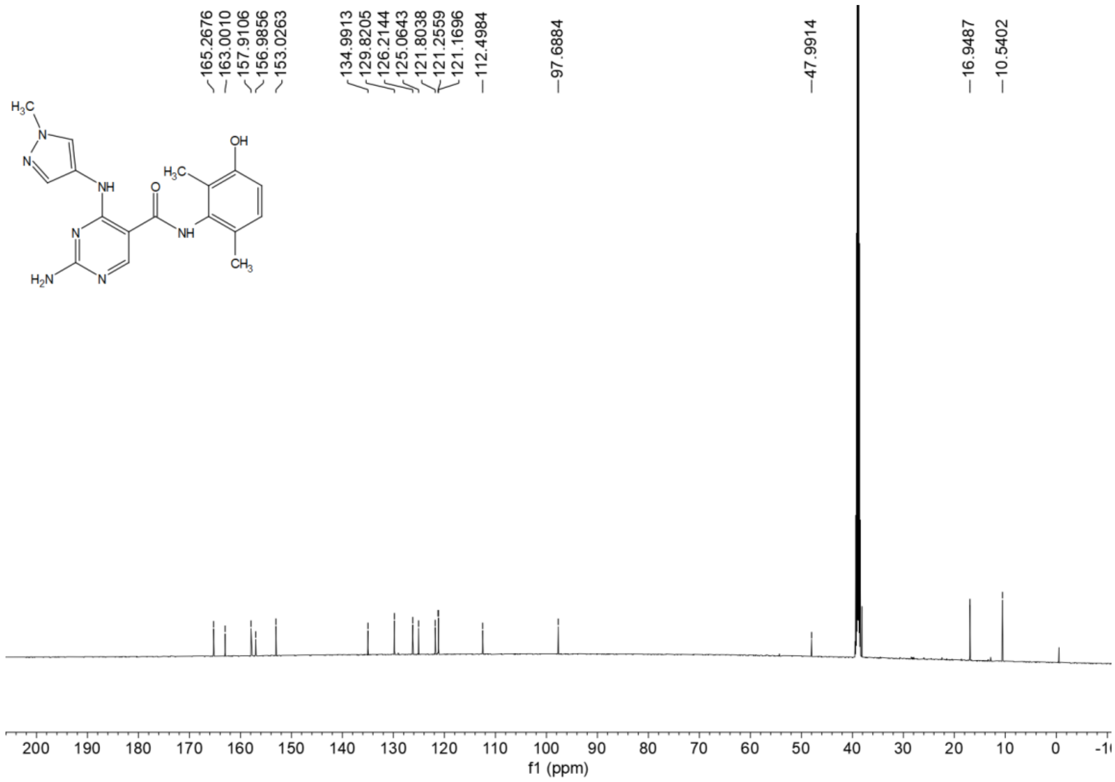

Fragmentor Voltage	Collision Energy	Ionization Mode
200	0	ESI

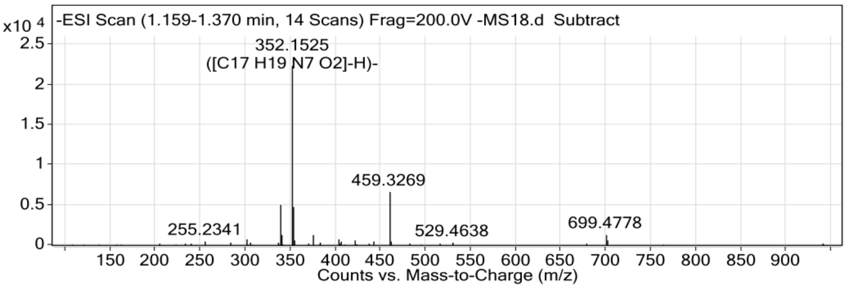

Peak List

m/z	z	Abund	Formula	Ion
301.1601	1	768.05		
339.2329	1	5041.79		
340.2352	1	1287.7		
352.1525	1	22397.38	C17 H19 N7 O2	(M-H)-
353.1555	1	4795.95	C17 H19 N7 O2	(M-H)-
374.1337	1	1304.92		
403.264	1	805.49		
459.3269	1	6681.71		
460.3291	1	2424.68		
699.4778	1	1362.93		

[illegible]

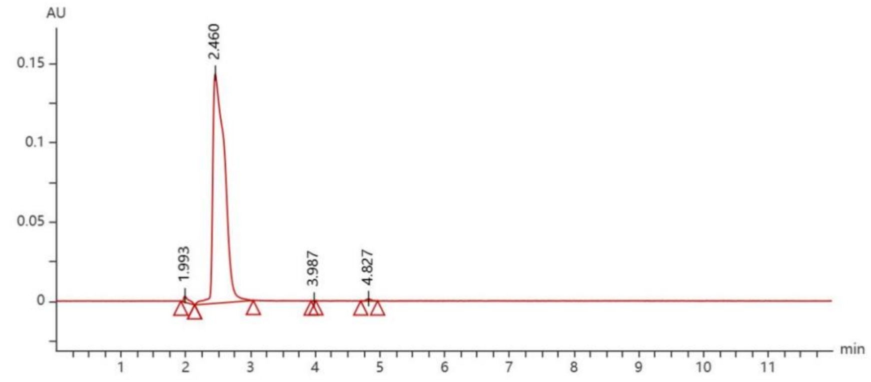

Object List

#	RT (min)	Area (µV*s)	% Area	Height (µV)	% Height
1	1.993	21995.600	1.182	3723.500	2.482
2	2.460	1831583.200	98.390	144902.397	96.575
3	3.987	474.467	0.025	211.909	0.141
4	4.827	7505.317	0.403	1204.077	0.802

^{1}H NMR, ^{13}C NMR, HRMS and HPLC spectra of Compound MS16

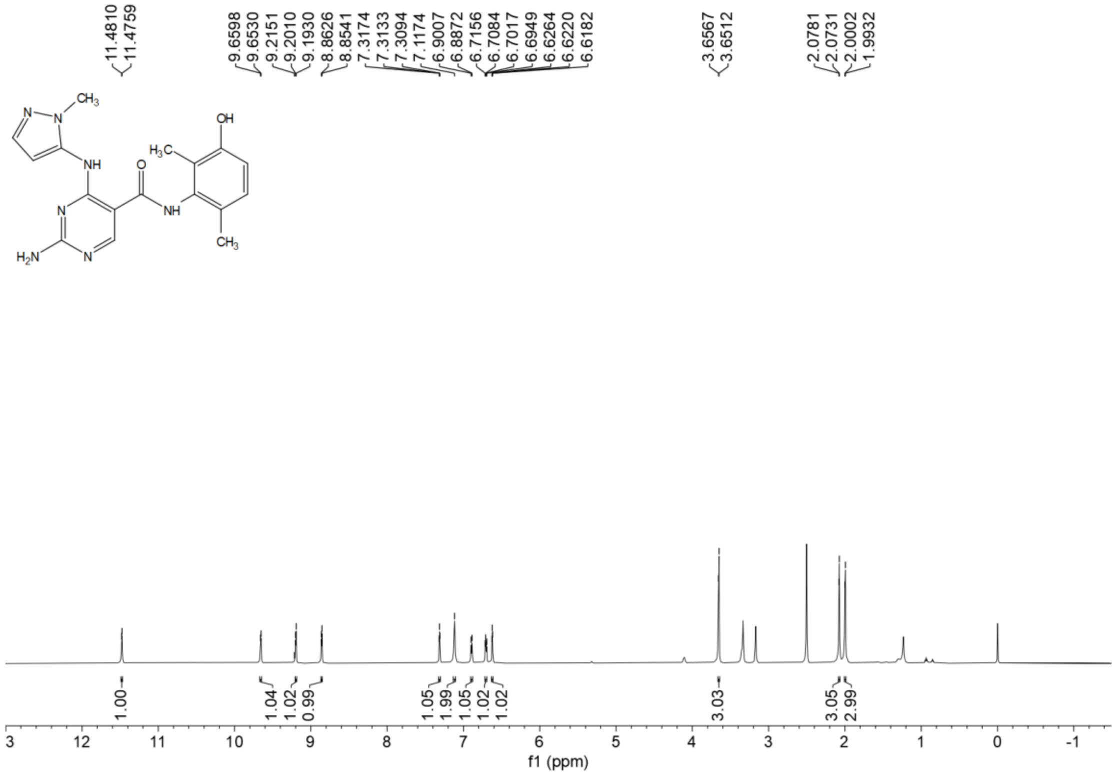

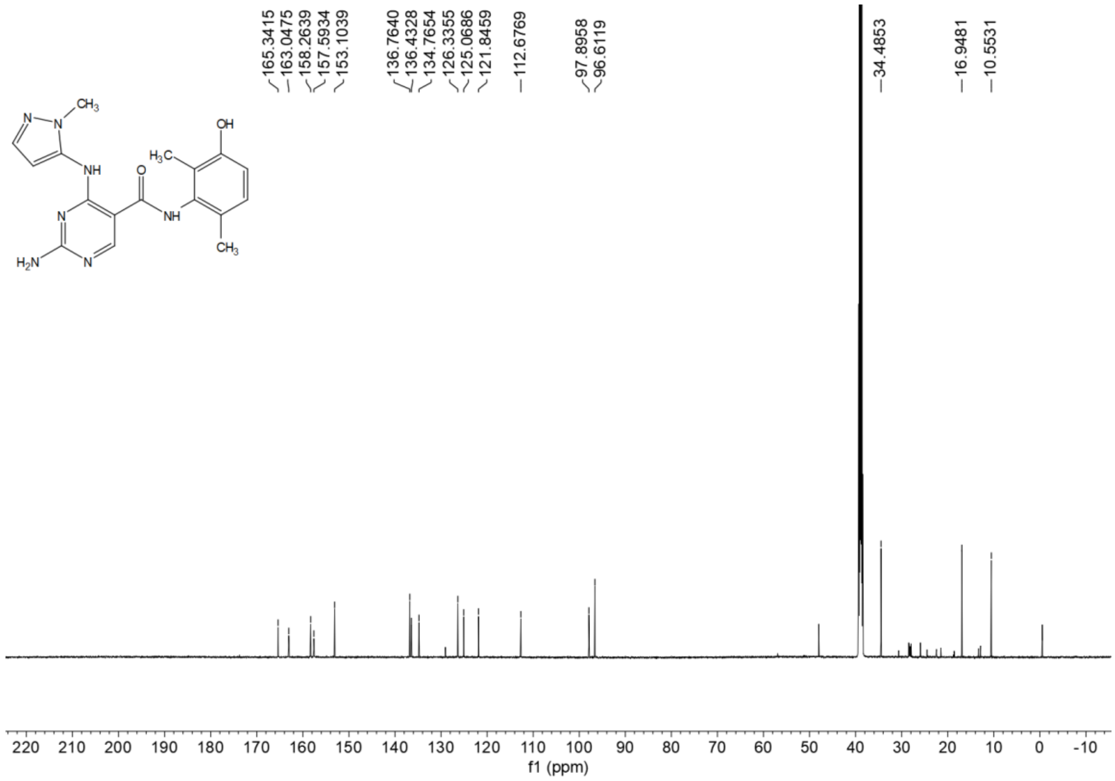

Fragmentor Voltage	Collision Energy	Ionization Mode
200	0	ESI

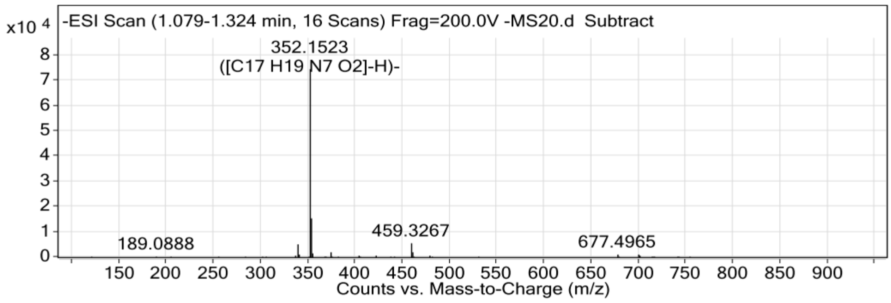

Peak List

m/z	z	Abund	Formula	Ion
339.2321	1	5178.46		
340.2359	1	1383.96		
352.1523	1	74634.66	C17 H19 N7 O2	(M-H)-
353.1546	1	15518.61	C17 H19 N7 O2	(M-H)-
354.1572	1	1902.09	C17 H19 N7 O2	(M-H)-
374.134	1	2121.98		
459.3267	1	5766.59		
460.3304	1	2101.75		
677.4965	1	1360.81		
699.477	1	1239.66		

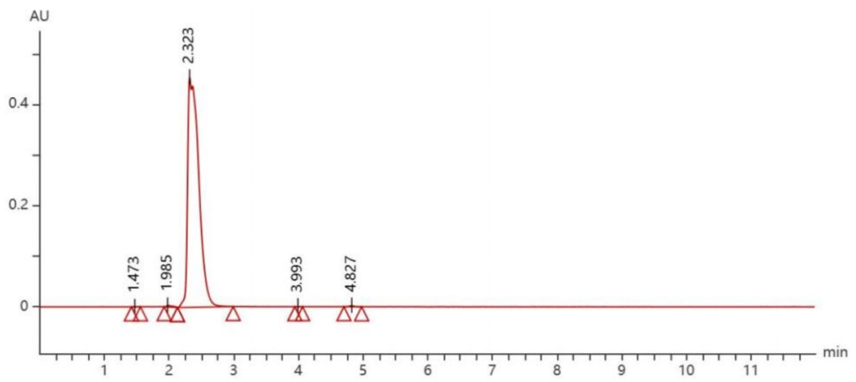

Object List

#	RT (min)	Area (µV*s)	% Area	Height (µV)	% Height
1	1.473	1181.575	0.022	216.762	0.047
2	1.985	21870.867	0.399	3410.984	0.740
3	2.323	5448164.783	99.329	455200.524	98.717
4	3.993	1233.667	0.022	304.222	0.066
5	4.827	12505.133	0.228	1981.872	0.430

^{1}H NMR, ^{13}C NMR, HRMS and HPLC spectra of Compound MS17

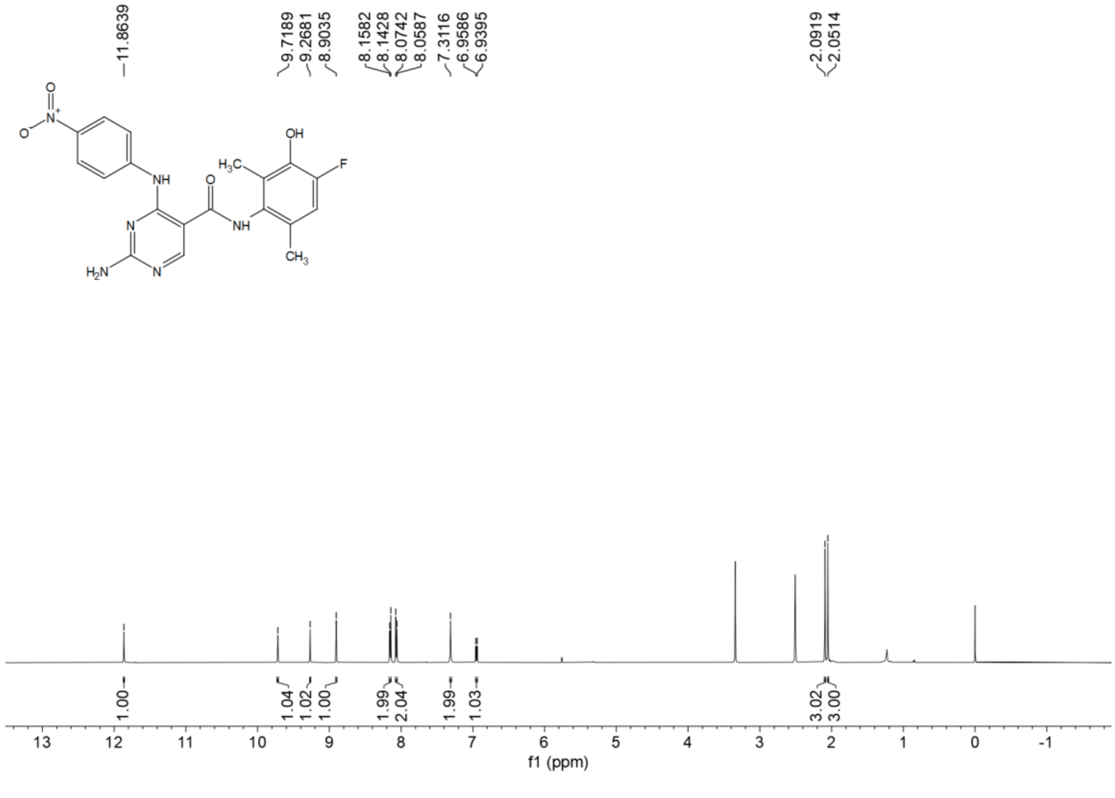

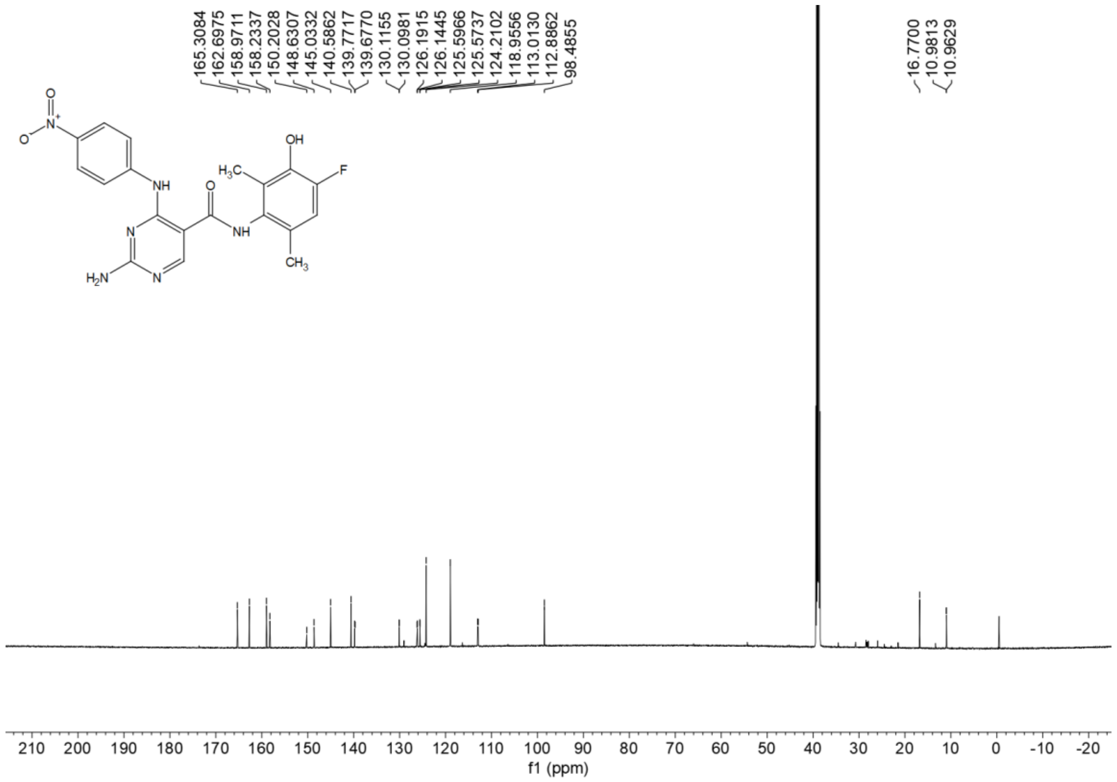

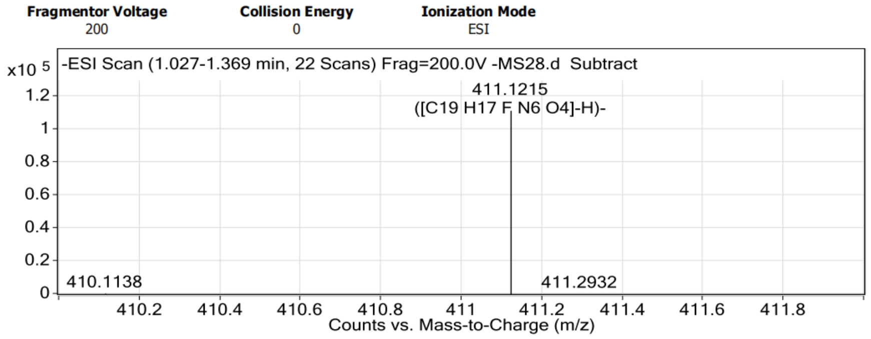

Peak List

m/z	z	Abund	Formula	Ion
137.0335	1	1142.12		
336.3264	1	1080.49		
339.2324	1	3134.89		
340.2356	1	803.48		
411.1215	1	111141.74	C19 H17 F N6 O4	(M-H)-
412.1243	1	24047.52	C19 H17 F N6 O4	(M-H)-
413.1276	1	3575.54	C19 H17 F N6 O4	(M-H)-
433.1031	1	2447.44		
459.3253	1	1144.78		
496.1007	1	699.99		

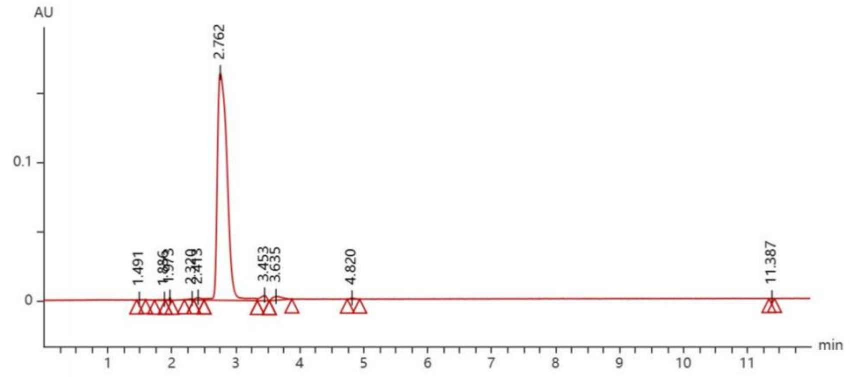

Object List

#	RT (min)	Area (µV*s)	% Area	Height (µV)	% Height
1	1.491	1151.233	0.063	261.794	0.148
2	1.886	3138.408	0.173	493.333	0.279
3	1.973	4387.776	0.242	2032.439	1.149
4	2.320	4412.261	0.243	905.511	0.512
5	2.413	11751.505	0.648	1682.020	0.951
6	2.762	1727698.124	95.239	163994.811	92.751
7	3.453	25770.568	1.421	3724.118	2.106
8	3.635	30397.042	1.676	2711.489	1.534
9	4.820	4800.017	0.265	791.172	0.447
10	11.387	550.125	0.030	215.308	0.122

^{1}H NMR, ^{13}C NMR, HRMS and HPLC spectra of Compound MS18

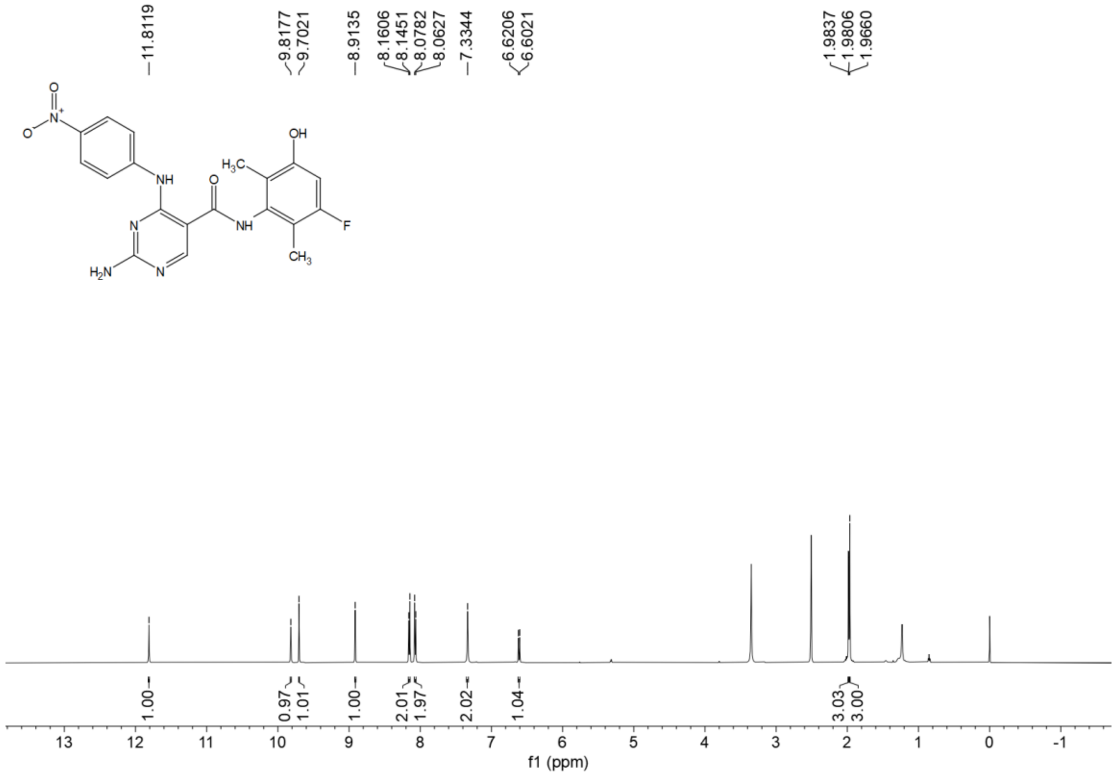

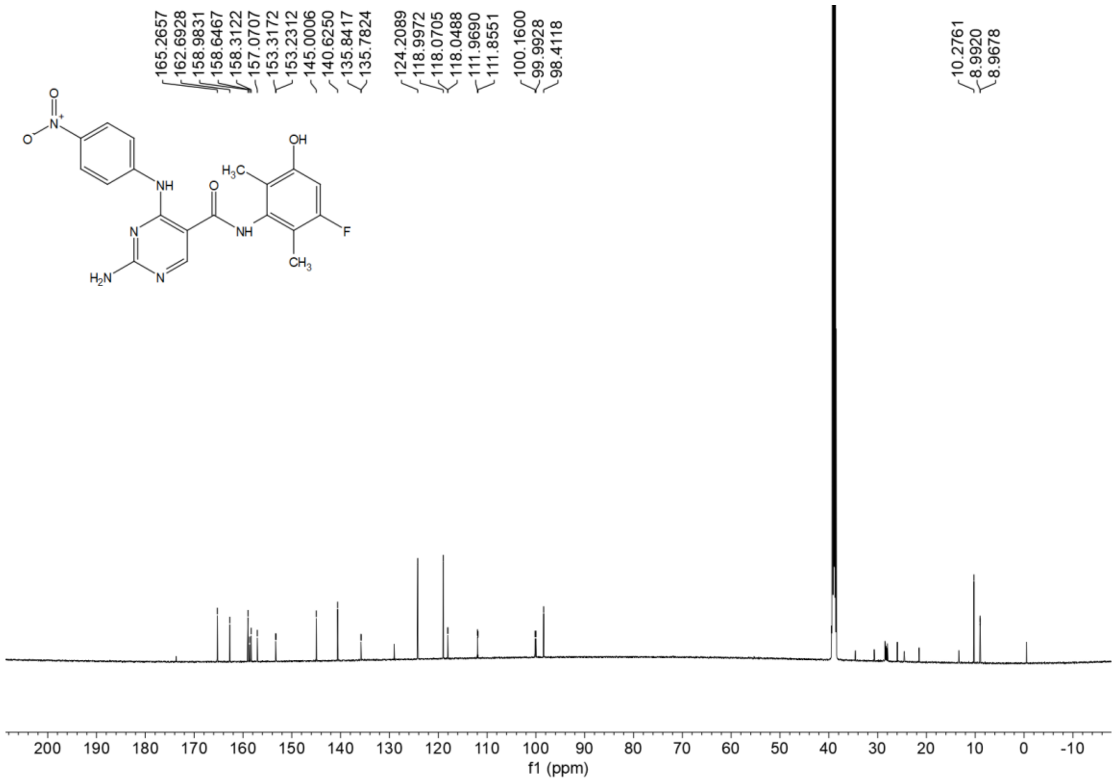

Fragmentor Voltage	Collision Energy	Ionization Mode
200	0	ESI

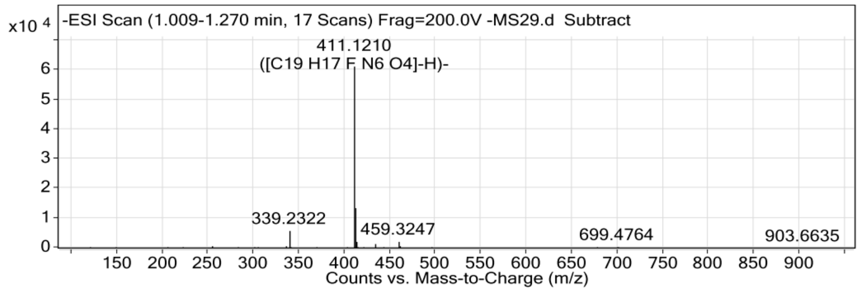

Peak List

m/z	z	Abund	Formula	Ion
255.2314	1	644.29		
336.3263	1	681.2		
339.2322	1	5966.46		
340.235	1	1591.29		
411.121	1	61326.11	C19 H17 F N6 O4	(M-H)-
412.1237	1	13622.68	C19 H17 F N6 O4	(M-H)-
413.1262	1	2102.58	C19 H17 F N6 O4	(M-H)-
433.103	1	1524.83		
459.3247	1	2373.8		
460.3287	1	890.39		

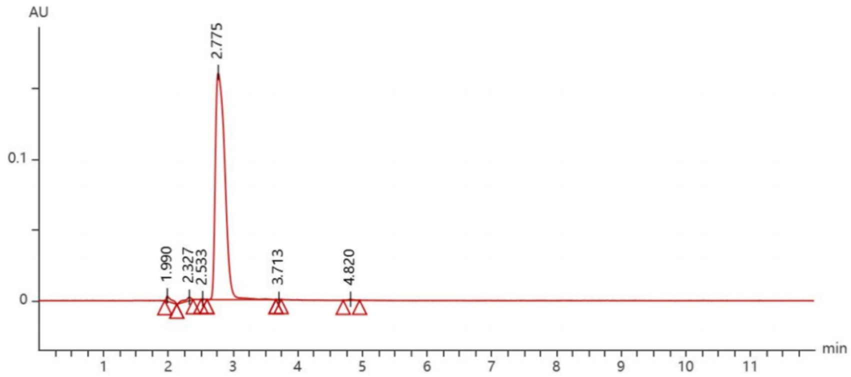

Object List

#	RT (min)	Area (µV*s)	% Area	Height (µV)	% Height
1	1.990	21466.733	1.276	3577.290	2.137
2	2.327	20072.260	1.193	2469.191	1.475
3	2.533	839.733	0.050	296.179	0.177
4	2.775	1634264.767	97.171	160286.542	95.738
5	3.713	364.233	0.022	131.188	0.078
6	4.820	4829.267	0.287	661.697	0.395

^{1}H NMR, ^{13}C NMR, HRMS and HPLC spectra of Compound MS19

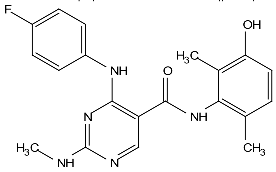

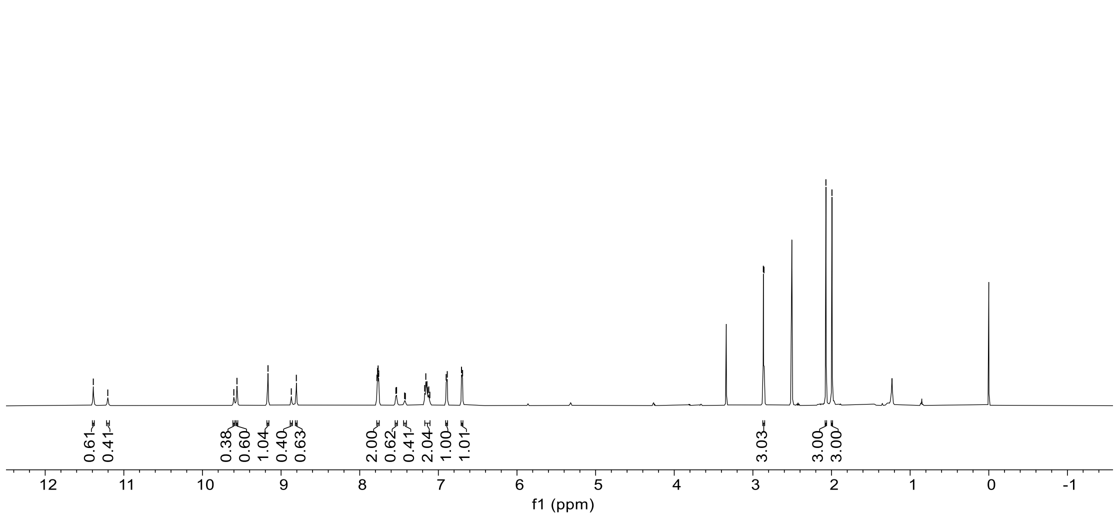

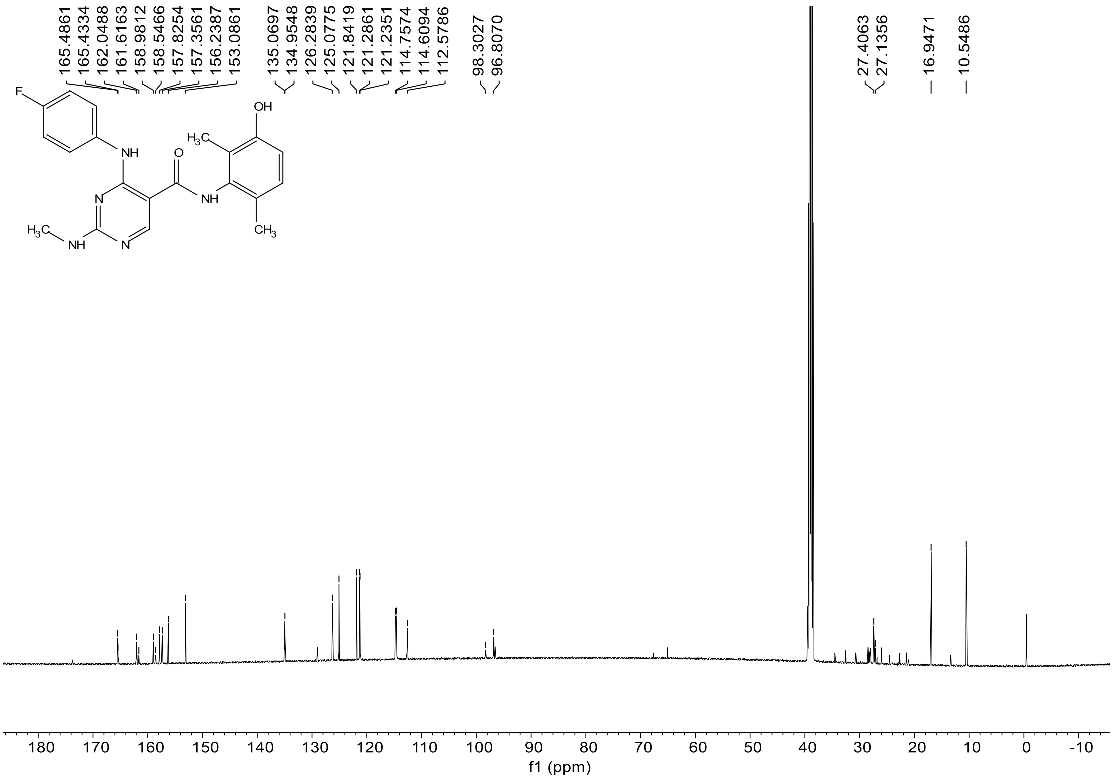

Fragmentor Voltage	Collision Energy	Ionization Mode
200	0	ESI

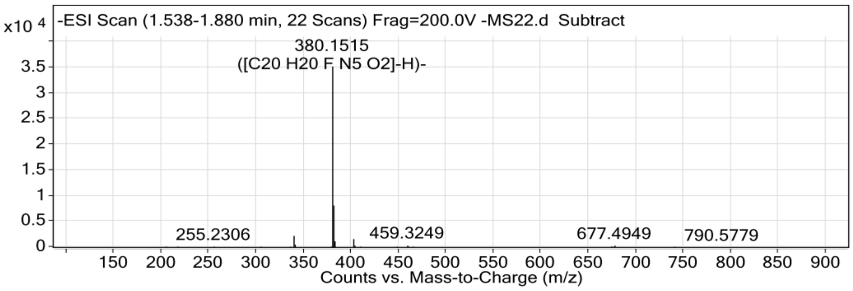

Peak List

m/z	z	Abund	Formula	Ion
339.2316	1	2304.55		
340.2355	1	573.36		
380.1515	1	35232.73	C20 H20 F N5 O2	(M-H)-
381.1543	1	8196.08	C20 H20 F N5 O2	(M-H)-
382.1562	1	1171.65	C20 H20 F N5 O2	(M-H)-
402.1342	1	1728.5		
403.1375	1	448.52		
459.3249	1	500.77		
465.132		249.45		
677.4949	1	385.76		

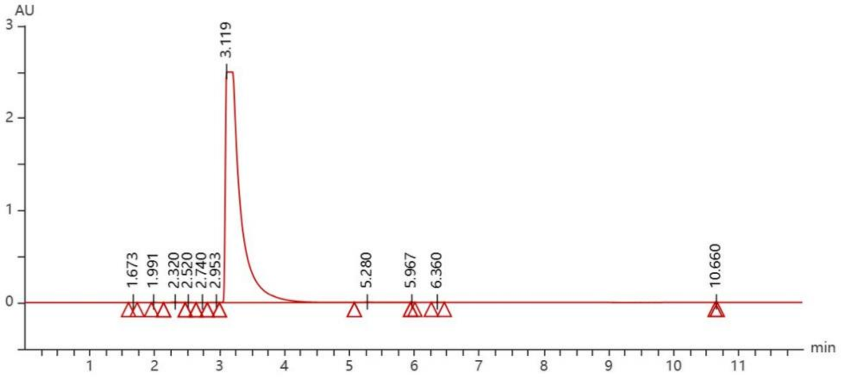

Object List

#	RT (min)	Area (µV*s)	% Area	Height (µV)	% Height
1	1.673	1345.367	0.003	388.238	0.015
2	1.991	23716.386	0.059	3830.761	0.148
3	2.320	78675.201	0.195	6137.455	0.237
4	2.520	32442.884	0.080	4182.933	0.162
5	2.740	27283.624	0.068	2812.184	0.109
6	2.953	41367.458	0.102	4332.011	0.168
7	3.119	40120875.482	99.361	2560559.729	99.058
8	5.280	51036.286	0.126	2039.330	0.079
9	5.967	454.648	0.001	202.780	0.008
10	6.360	1310.267	0.003	223.183	0.009
11	10.660	201.892	0.000	200.800	0.008

^{1}H NMR, ^{13}C NMR, HRMS and HPLC spectra of Compound MS20

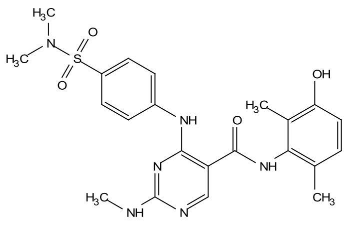

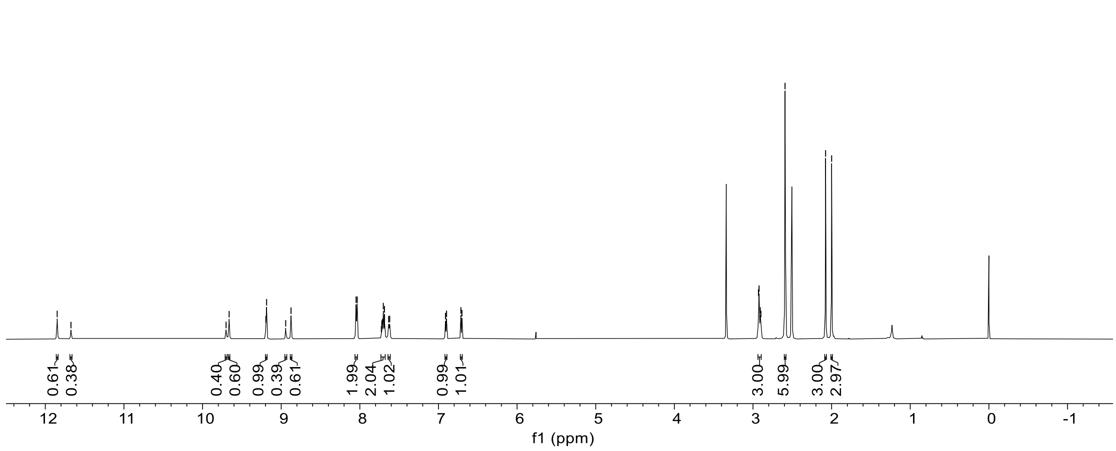

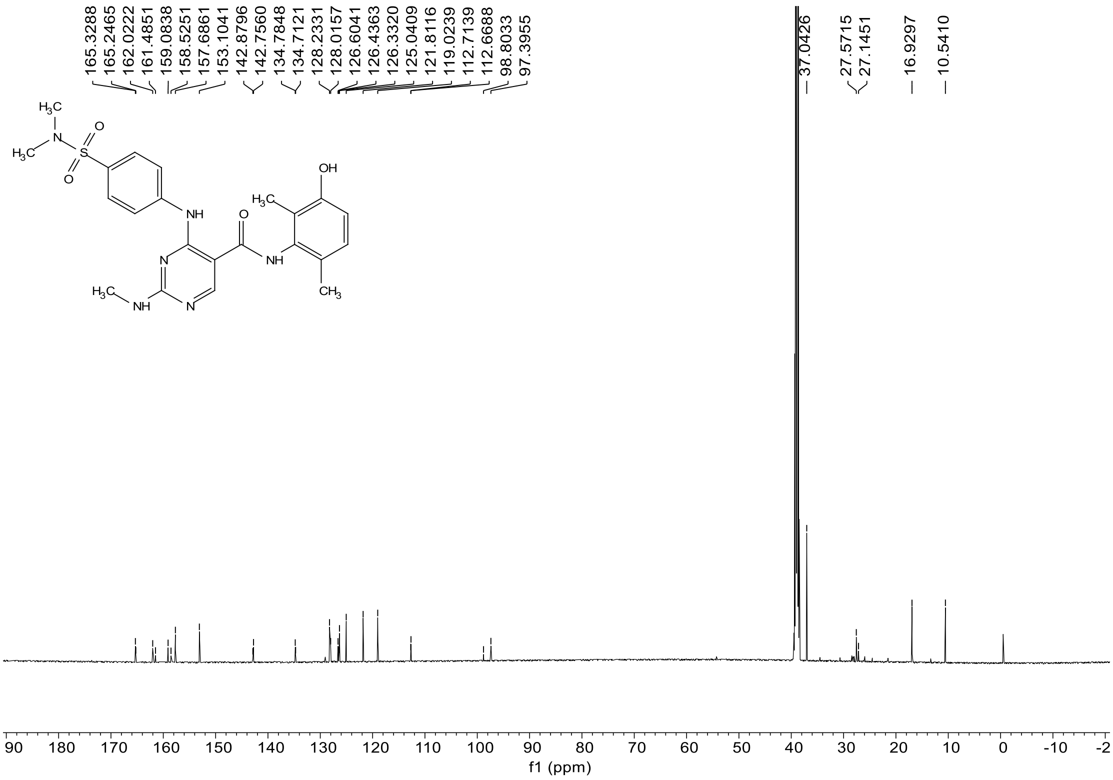

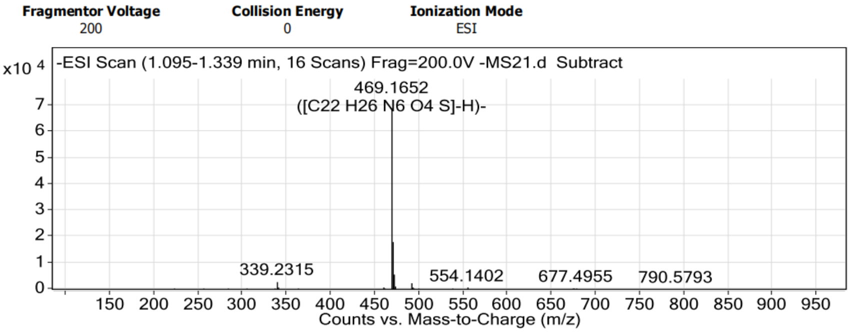

Peak List

m/z	z	Abund	Formula	Ion
339.2315	1	3028.86		
340.2347	1	767.01		
459.3276	1	664.49		
469.1652	1	68848.67	C22 H26 N6 O4 S	(M-H)-
470.1679	1	17974.42	C22 H26 N6 O4 S	(M-H)-
471.1653	1	5747.46	C22 H26 N6 O4 S	(M-H)-
472.1686	1	1166.57	C22 H26 N6 O4 S	(M-H)-
491.1473	1	2654.32		
492.1489	1	798.64		
554.1402	1	628.14		

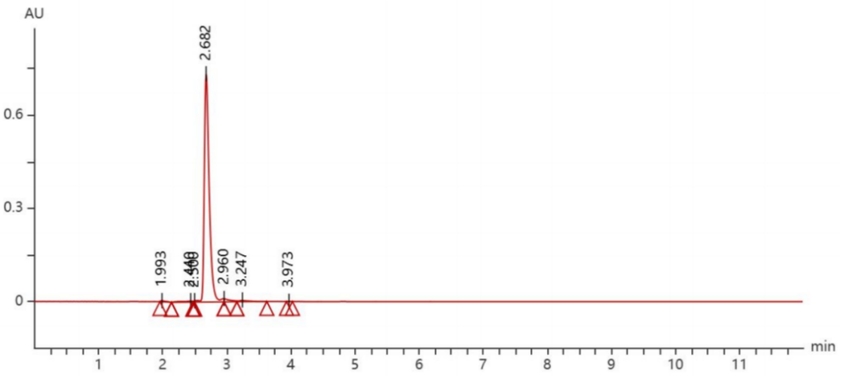

Object List

#	RT (min)	Area (µV*s)	% Area	Height (µV)	% Height
1	1.993	18688.031	0.456	3356.497	0.443
2	2.440	45925.630	1.120	3269.942	0.431
3	2.500	5151.808	0.126	3518.880	0.464
4	2.682	3921484.091	95.598	733834.359	96.780
5	2.960	64200.588	1.565	10217.074	1.347
6	3.247	45874.292	1.118	3842.307	0.507
7	3.973	738.400	0.018	214.643	0.028

^{1}H NMR, ^{13}C NMR, HRMS and HPLC spectra of Compound MS21

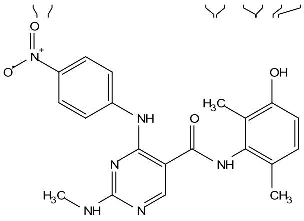

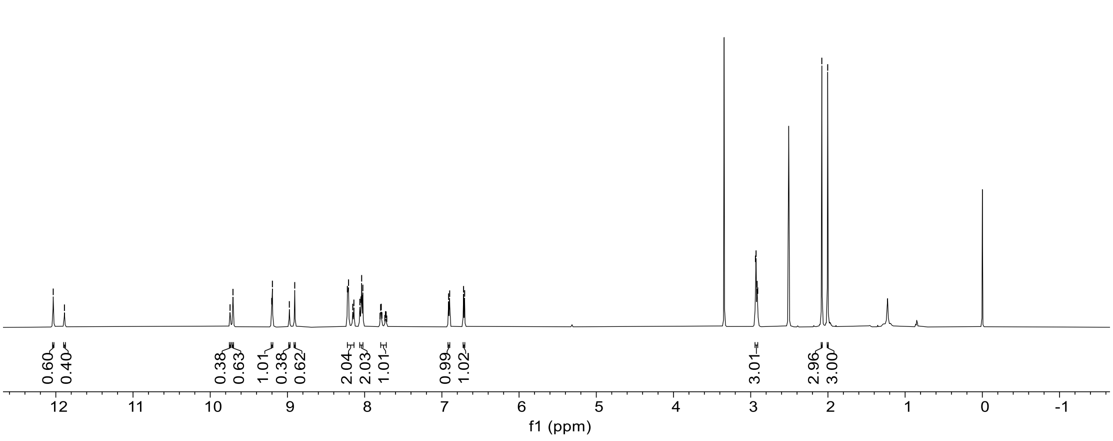

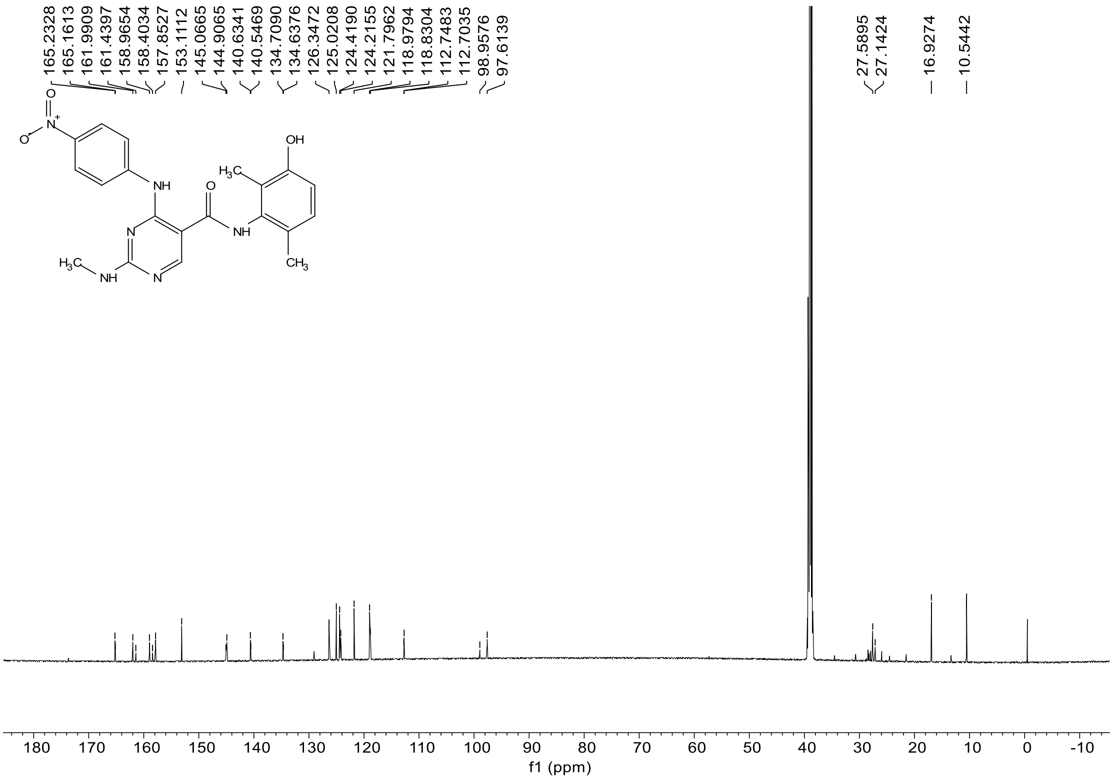

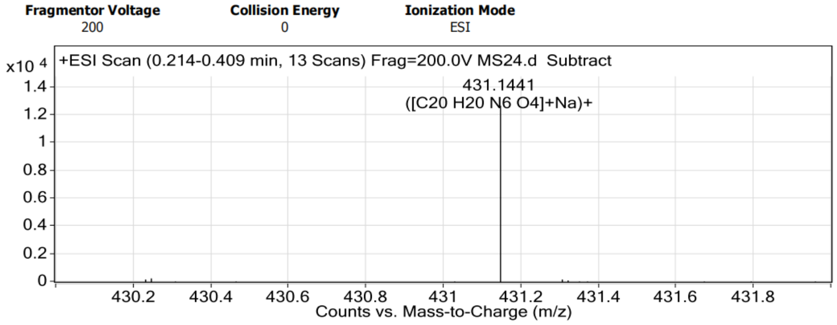

Peak List

m/z	z	Abund	Formula	Ion
249.1578	1	88458.74		
250.162	1	11858.63		
362.2423	2	42134.86		
362.7433	2	9237.4		
431.1441	1	12711.69	C20 H20 N6 O4	(M+Na)+
475.3269	1	217592.72		
476.3294	1	57482.51		
477.3307	1	9096.37		
701.4945	1	47427.39		
702.4972	1	20137.84		

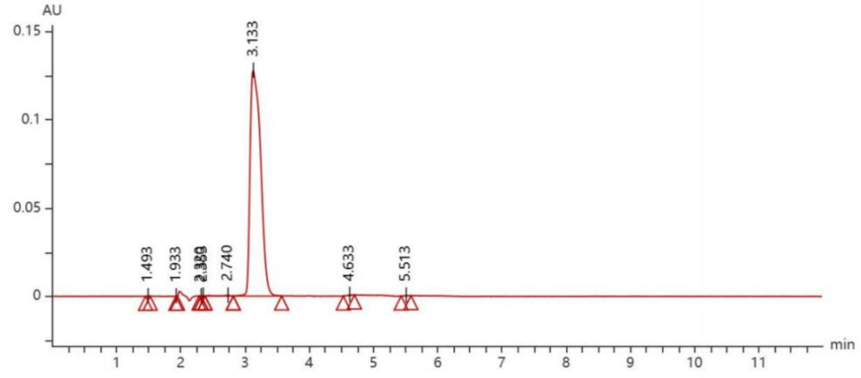

Object List

#	RT (min)	Area (µV*s)	% Area	Height (µV)	% Height
1	1.493	373.900	0.026	163.341	0.126
2	1.933	94.767	0.006	157.188	0.122
3	2.320	23.882	0.002	-95.828	0.074
4	2.353	405.031	0.028	220.345	0.171
5	2.740	5384.786	0.368	464.693	0.360
6	3.133	1456329.030	99.404	127627.276	98.797
7	4.633	1407.467	0.096	251.875	0.195
8	5.513	1048.625	0.072	200.967	0.156

^{1}H NMR, ^{13}C NMR, HRMS and HPLC spectra of Compound MS22

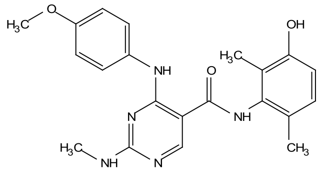

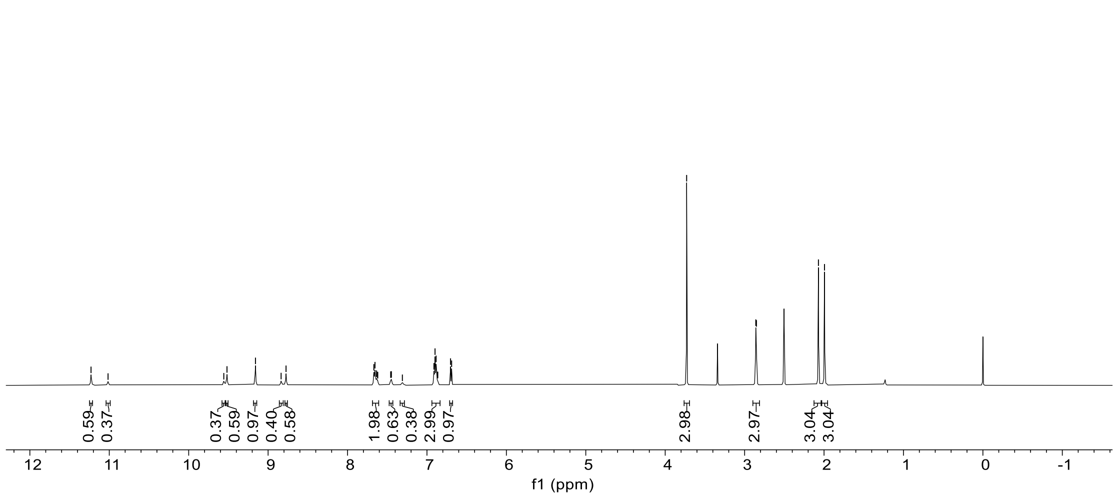

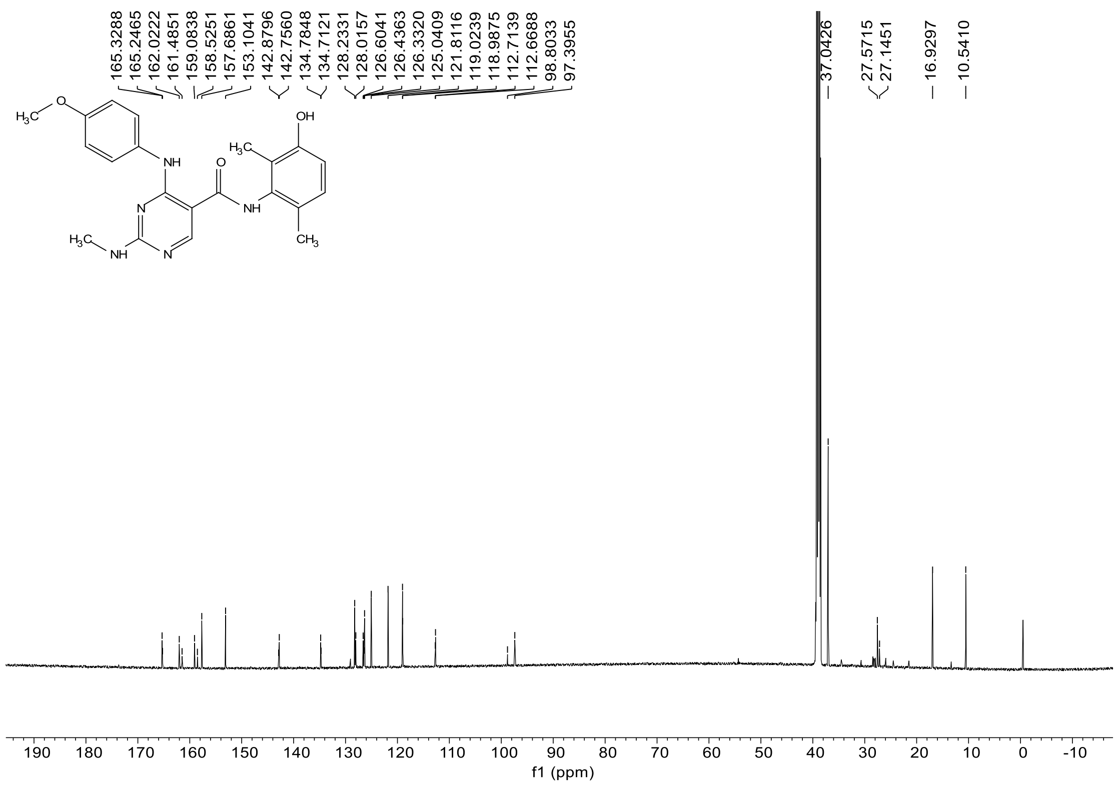

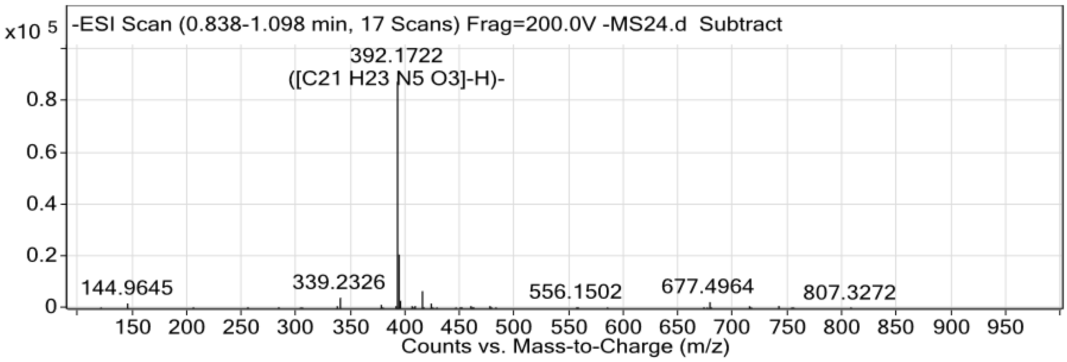

Peak List

m/z	z	Abund	Formula	Ion
144.9645		2253.76		
339.2326	1	4448.05		
377.1482	1	1708.45		
392.1722	1	87635.88	C21 H23 N5 O3	(M-H)-
393.1753	1	20714.37	C21 H23 N5 O3	(M-H)-
394.1774	1	3184.63	C21 H23 N5 O3	(M-H)-
414.1538	1	6771.91		
415.1569	1	1771.86		
422.1822	1	1921.75		
677.4964	1	2764.44		

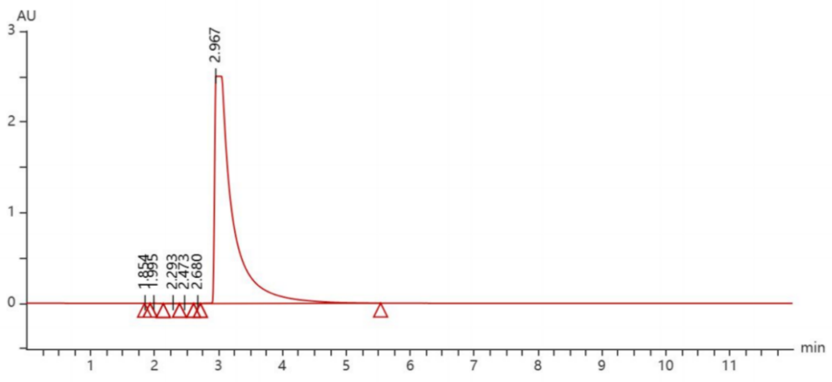

Object List

#	RT (min)	Area (µV*s)	% Area	Height (µV)	% Height
1	1.854	176.088	0.000	82.090	0.003
2	1.995	23675.356	0.048	3724.624	0.145
3	2.293	35008.506	0.071	2933.237	0.114
4	2.473	36423.239	0.074	3434.265	0.133
5	2.680	17075.171	0.035	2849.334	0.111
6	2.967	49222643.108	99.772	2562227.240	99.494

^{1}H NMR, ^{13}C NMR, HRMS and HPLC spectra of Compound MS23

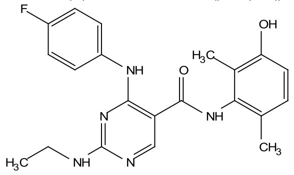

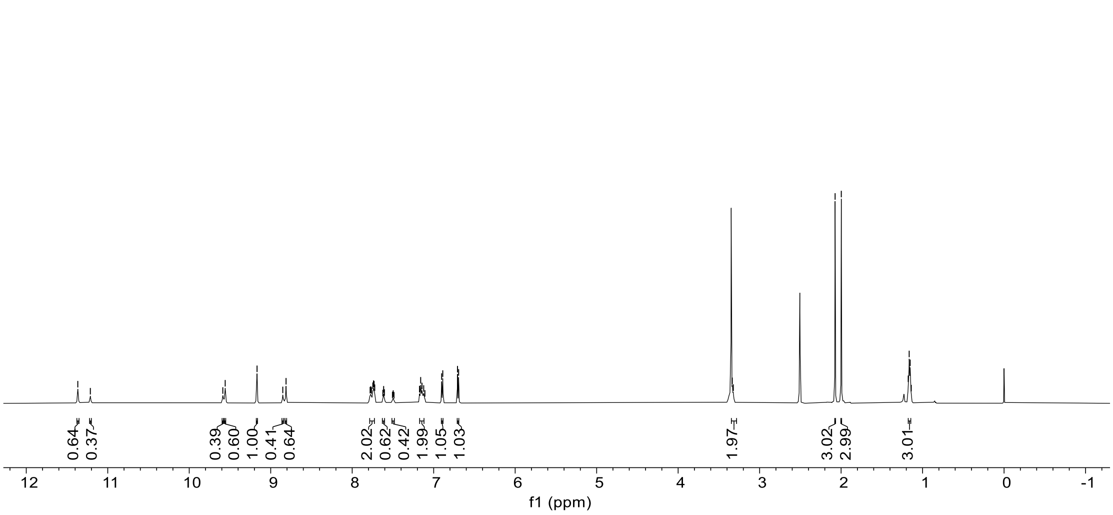

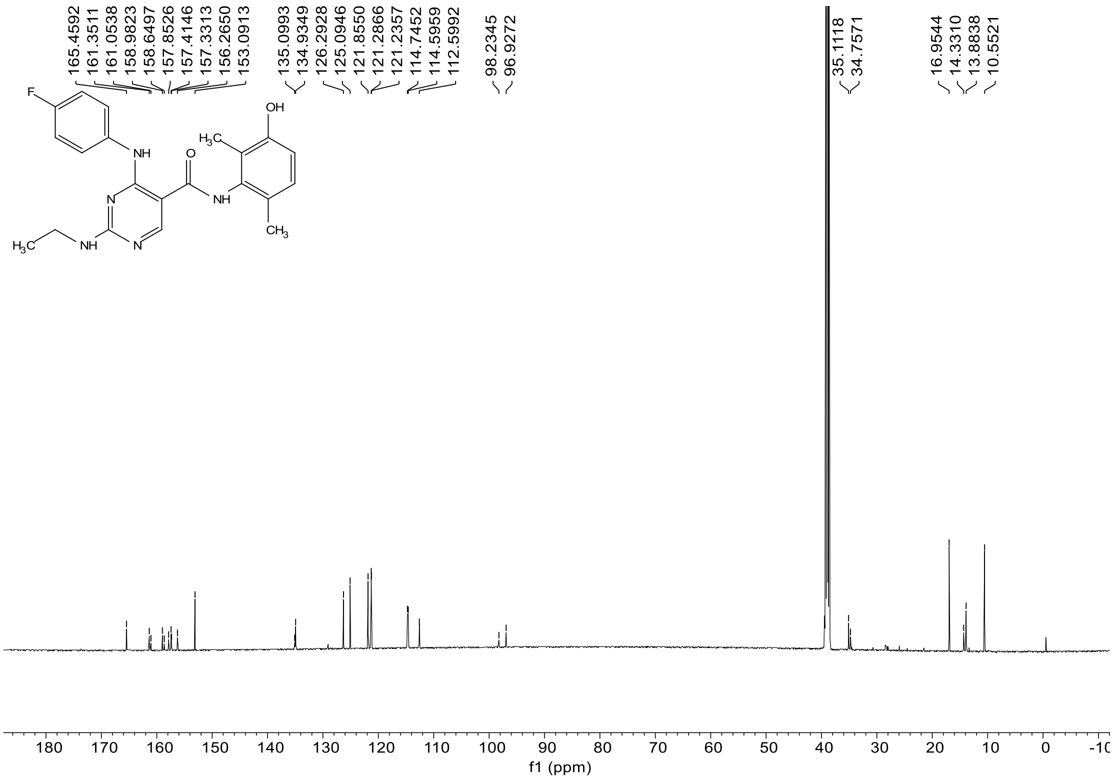

Fragmentor Voltage	Collision Energy	Ionization Mode
200	0	ESI

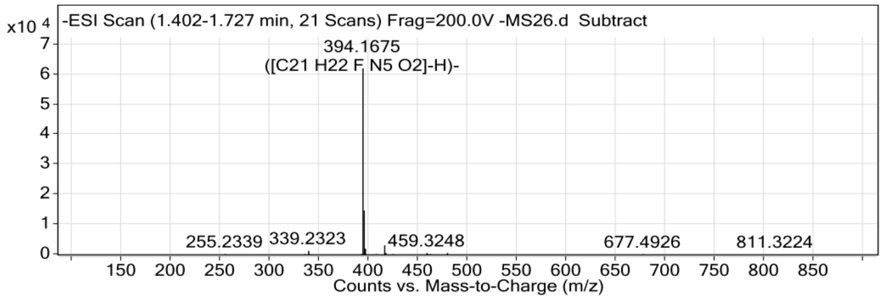

Peak List

m/z	z	Abund	Formula	Ion
339.2323	1	1374.48		
393.1589		395.81		
394.1675	1	62164.2	C21 H22 F N5 O2	(M-H)-
395.1704	1	15048.15	C21 H22 F N5 O2	(M-H)-
395.3334	1	401.6		
396.1726	1	2151.95	C21 H22 F N5 O2	(M-H)-
416.1491	1	3241.87		
417.1524	1	860.41		
459.3248	1	615.4		
479.1446	1	583.36		

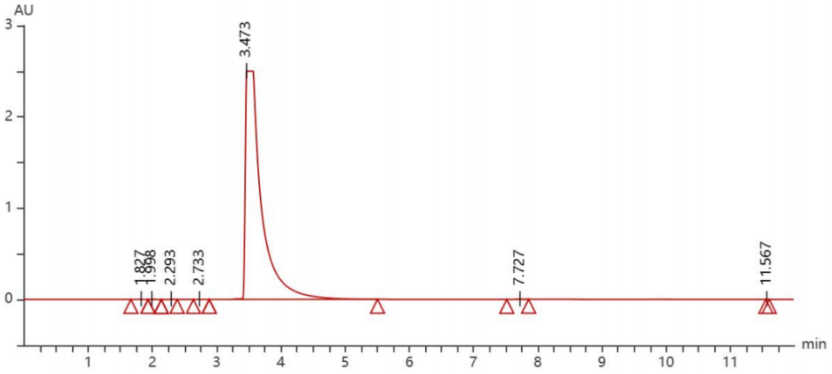

Object List

#	RT (min)	Area (µV*s)	% Area	Height (µV)	% Height
1	1.827	7142.500	0.016	1608.550	0.063
2	1.998	24330.425	0.056	3821.400	0.149
3	2.293	21699.400	0.050	2245.291	0.088
4	2.733	3586.483	0.008	607.223	0.024
5	3.473	43688024.592	99.860	2555928.305	99.657
6	7.727	4301.208	0.010	347.407	0.014
7	11.567	305.667	0.001	166.500	0.006

^{1}H NMR, ^{13}C NMR, HRMS and HPLC spectra of Compound MS24

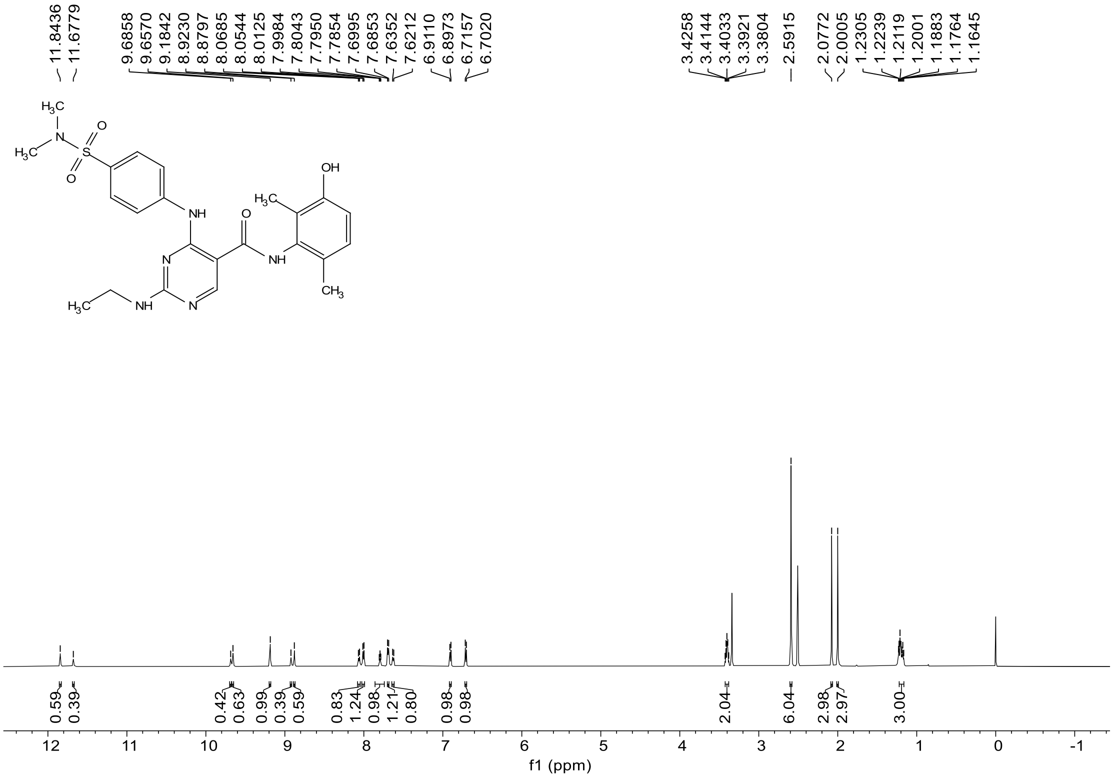

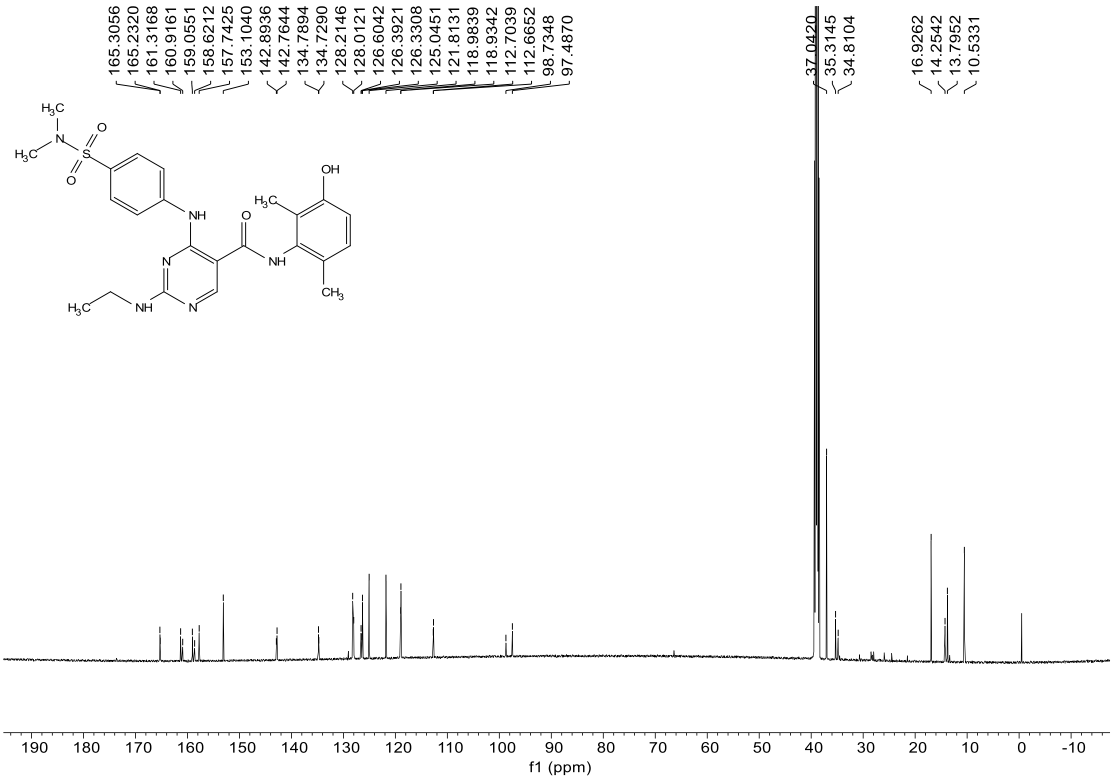

Fragmentor Voltage	Collision Energy	Ionization Mode
200	0	ESI

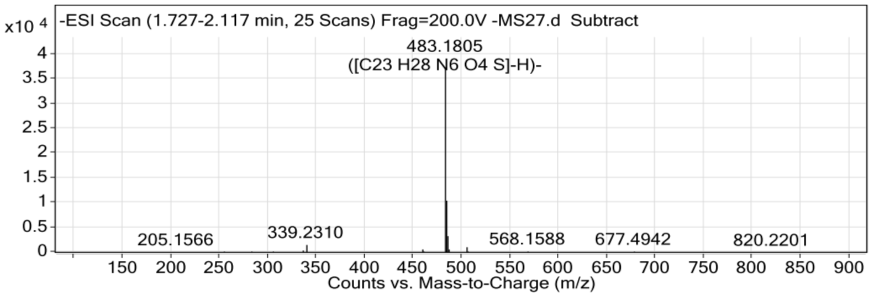

Peak List

m/z	z	Abund	Formula	Ion
336.3261	1	374.73		
339.231	1	1576.38		
340.2347	1	379.21		
459.325	1	584.26		
483.1805	1	37309.73	C23 H28 N6 O4 S	(M-H)-
484.1836	1	10416.43	C23 H28 N6 O4 S	(M-H)-
485.1818	1	3346.37	C23 H28 N6 O4 S	(M-H)-
486.1825	1	707.49	C23 H28 N6 O4 S	(M-H)-
505.1619	1	1169.71		
506.1655	1	334.18		

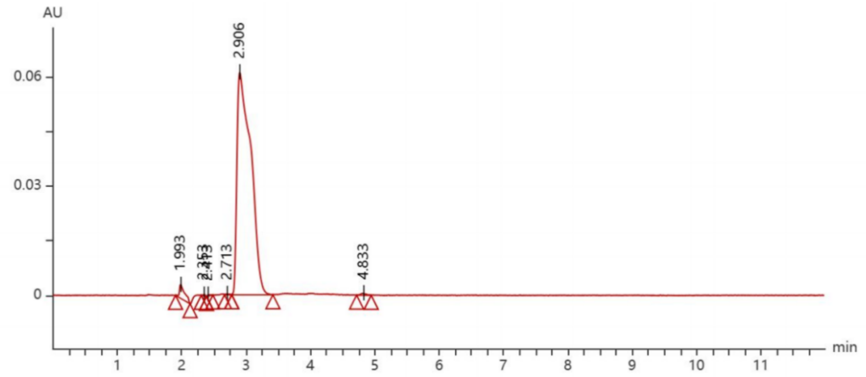

Object List

#	RT (min)	Area (µV*s)	% Area	Height (µV)	% Height
1	1.993	22487.133	2.384	3740.801	5.626
2	2.353	1004.833	0.107	453.688	0.682
3	2.413	1108.933	0.118	374.967	0.564
4	2.713	1103.642	0.117	275.792	0.415
5	2.906	914196.374	96.935	61151.117	91.967
6	4.833	3199.667	0.339	496.000	0.746
